# Computational Design, Synthesis, and Biological Evaluation of Diimidazole Analogues Endowed with Dual PCSK9/HMG-CoAR-Inhibiting Activity

**DOI:** 10.1021/acs.jmedchem.3c00279

**Published:** 2023-06-01

**Authors:** Carmen Lammi, Enrico M. A. Fassi, Marco Manenti, Marta Brambilla, Maria Conti, Jianqiang Li, Gabriella Roda, Marina Camera, Alessandra Silvani, Giovanni Grazioso

**Affiliations:** † Dipartimento di Scienze Farmaceutiche, Università degli Studi di Milano, Via L. Mangiagalli 25, 20133 Milan, Italy; ‡ Dipartimento di Chimica, Università degli Studi di Milano, Via Golgi 10, 20133 Milan, Italy; § Centro Cardiologico Monzino IRCCS, via Parea 4, 20138 Milan, Italy

## Abstract

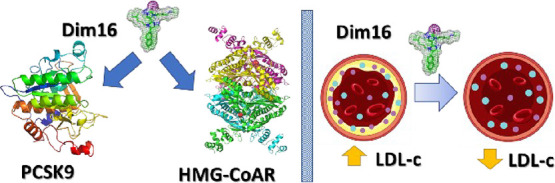

Proprotein convertase subtilisin/kexin 9 (PCSK9) is responsible for the degradation of the hepatic low-density lipoprotein receptor (LDLR), which regulates circulating cholesterol levels. Consequently, the PCSK9 inhibition is a valuable therapeutic approach for the treatment of hypercholesterolemia and cardiovascular diseases. In our studies, we discovered **Rim13**, a polyimidazole derivative reducing the protein–protein interaction between PCSK9 and LDLR with an IC_50_ of 1.6 μM. The computational design led to the optimization of the shape of the PCSK9/ligand complementarity, enabling the discovery of potent diimidazole derivatives. In fact, carrying out biological assays to fully characterize the cholesterol-lowering activity of the new analogues and using both biochemical and cellular techniques, compound **Dim16** displayed improved PCSK9 inhibitory activity (IC_50_ 0.9 nM). Interestingly, similar to other lupin-derived peptides and their synthetic analogues, some compounds in this series showed dual hypocholesterolemic activity since some of them complementarily inhibited the 3-hydroxy-3-methylglutaryl coenzyme A reductase.

## Introduction

Proprotein convertase subtilisin/kexin 9 (PCSK9) is a blood-circulating enzyme responsible for the regulation of the low-density lipoprotein receptor (LDLR) population on the liver cell surface. Formation of the PCSK9/LDLR complex leads to cell internalization and degradation of LDLR, diminishing the liver cells’ capacity to capture blood-circulating LDL cholesterol (LDL-c). Accordingly, inhibition of the PCSK9/LDLR interaction leads to an increased LDLR population on the cell membrane, resulting in an augmented LDL-c uptake capacity of the liver cells. Besides playing a key role in the regulation of LDL metabolism, PCSK9 has been reported to be involved in several processes relevant for cardiovascular homeostasis.^1^ Indeed, levels of PCSK9 predict recurrent cardiovascular events in patients with coronary artery disease, even in those with well-controlled LDL-c levels.^2^ In this regard, compelling evidence highlights the emerging role of PCSK9 as a player in platelet reactivity and thrombus formation,^3,4^ thus suggesting the clinical relevance of its pharmacological inhibition.

**Figure 1:**
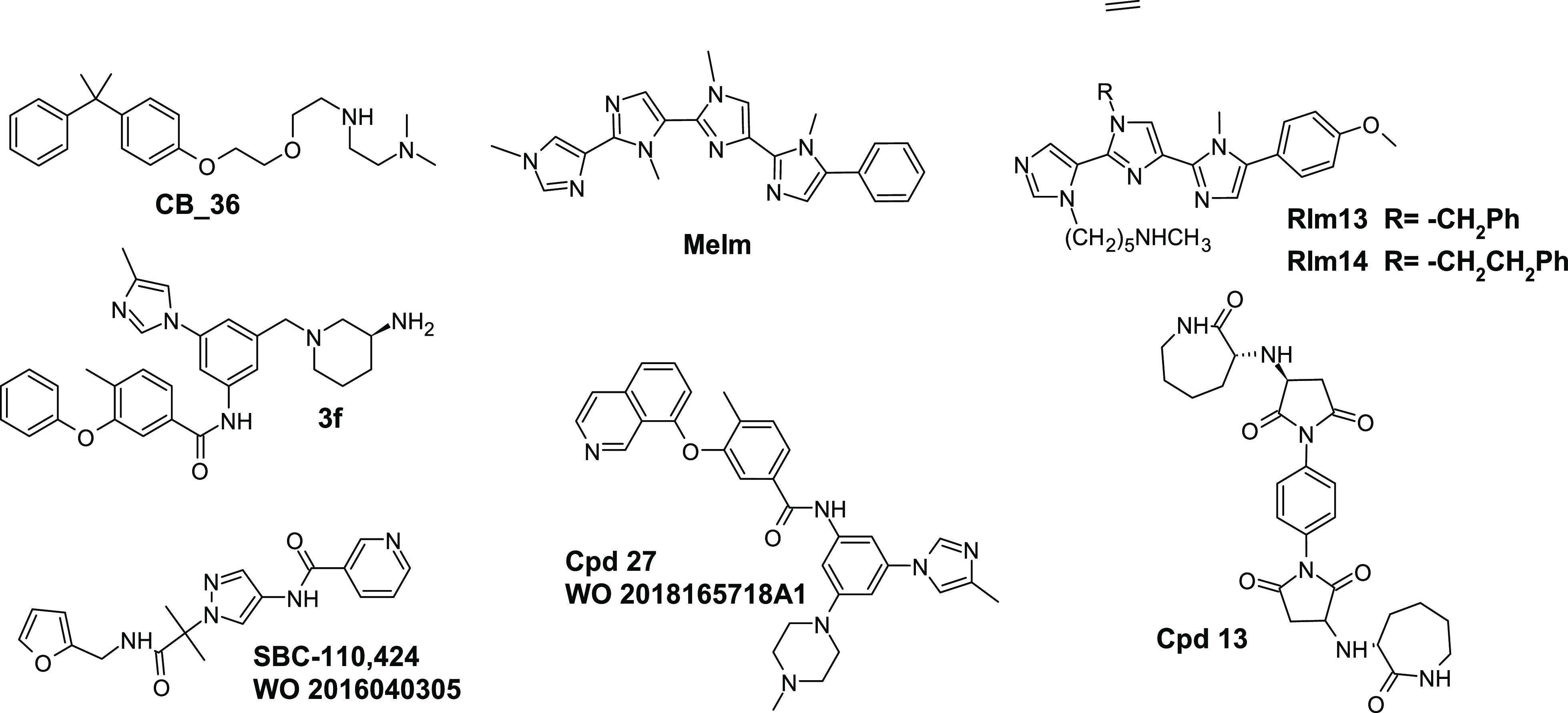
Structure of some known PCSK9 inhibitors reported in the literature and patents.

In recent years, considerable resources have been dedicated by academia and pharmaceutical companies to the identification of compounds capable of inhibiting PCSK9. A few years ago, the release on the market of two monoclonal antibodies—alirocumab (Praulent, Sanofi) and evolocumab (Repatha, Amgen)—proved that PCSK9 inhibition is a successful therapeutic approach for the treatment of statin-resistant hypercholesterolemia. Additionally, Novartis developed the first siRNA drug (Inclisiran, Leqvio)^5^ capable of interrupting the liver transcription of PCSK9, leading to persistent hypocholesterolemic effects in treated patients. Nevertheless, these drugs are expensive and do not elicit good patient compliance since they are subcutaneously administered. For these reasons, pharmaceutical companies and academia are greatly interested in the clinical development of orally bioavailable small molecules, as demonstrated by the high number of patent applications in this field.^6^ Among the best known PCSK9–LDLR interaction inhibitors, peptides have received attention since numerous research studies have been reported in the literature.^6−15^ In fact, peptides, or peptidomimetics, constitute a useful starting point for the identification of new drugs.^16−22^ In this regard, numerous small molecules have been reported in the literature, for example, Cpd13,^23^ CB36,^24^ 3f^19^, and **RIm13**,^25^ and in patents (Figure 1). Notably, some are in advanced clinical stages.

In our studies, taking inspiration from the β-strand of the LDLR EGF-A domain in complex with PCSK9 in the X-ray structure,^26^ we supposed that minimalist peptidomimetic polyimidazoles could represent promising PCSK9–LDLR interaction inhibitors.^22^ As proof of this, the simplest tetraimidazole MeIm displayed an IC_50_ value in the low-micromolar range.^22^ Then, optimizing the imidazole substitution pattern by computational studies, a triimidazole derivative (**Rim13**, Figure 1), displayed a PCSK9 IC_50_ value close to 1 μM.^25^ Similarly, literature evidence also highlights that synthetic^27,28^ and natural compounds, mainly food-derived peptides, exhibit the ability to impair PCSK9/LDLR interaction,^12,29^ and some display activity in the low micromolar range.^15^ Interestingly, some lupin-derived peptides demonstrated a peculiar feature of being able to decrease the activity of 3-hydroxy-3-methylglutaryl coenzyme A reductase (HMG-CoAR), which is the main target in the treatment of hypercholesterolemia.^30^ Therefore, through a dual mechanism, lupin peptides modulate cholesterol synthesis, which leads to improvement in LDLR protein levels and receptor stability due to the inhibition of PCSK9/LDLR interaction.

In this paper, the MeIm polyimidazole structure (Figure 1) has been further refined by designing novel diimidazole derivatives considering the high synthetic feasibility and higher affinity expected of PCSK9. By applying computational techniques, new PCSK9 inhibitors were designed, and a selected library of compounds was synthesized. Then, their biological activity was fully investigated by performing assays ranging from cell viability tests to the study of the modulation of the cholesterol pathway on the HepG2 cells, which were highly influenced by the dual inhibitory activity of some compounds. Finally, the pharmacokinetic properties of the most promising compounds were determined, and their antiplatelet activity was investigated.

## Results

### Computational Design Strategy

Our studies on PCSK9-inhibiting compounds started with the design of the polyimidazole called MeIm (Figure 1),^22^ for which the β-strand conformation of the EGF-A moiety in complex with PCSK9 served as a source of inspiration. Then, the structure of MeIm was progressively optimized to better fit the PCSK9 surface by adopting a computational procedure in which the SuMD,^31^ classical MD, cluster analysis, and molecular mechanics-generalized Born surface area (MM-GBSA) calculations were accomplished to design compounds **RIm13** and **RIm14** (Figure 1), displaying low micromolar affinity with PCSK9.^25^ Here, considering the structures of **RIm13**, new compounds were designed aiming to further improve the activity on the PCSK9 biochemical pathway. In the computational procedure adopted here, starting from the PCSK9 computational model we had previously developed,^32^ new polyimidazole analogues were designed (Table 1), estimating their binding free energy after docking calculations, pose selection by metadynamics simulations (to improve the accuracy of the binding pose selection), and molecular dynamics (MD) simulations.

**Table 1:**
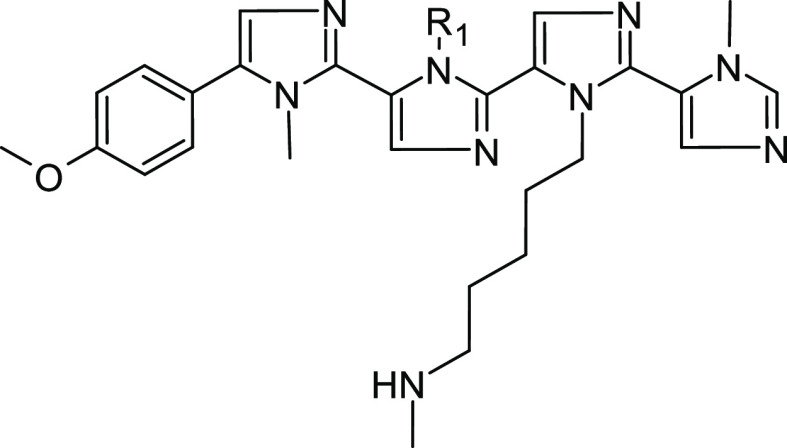
Chemical Structure and Estimated ΔG* Values of the New Series of Compound Deriving from **RIm13**

compound	R_1_	ΔG* ± SE^a^ (kcal/mol)
Tetra1	-CH_2_(*c*-C_5_H_9_)	–30.4 ± 0.4
Tetra2	-(CH_2_)_4_Me	–24.4 ± 0.4
Tetra3	-CH_2_(*c*-C_4_H_7_)	–25.5 ± 0.4
Tetra4	-CH_2_ (*c*-C_3_H_5_)	–26.0 ± 0.3
Tetra5	-(CH_2_)_3_Me	–26.1 ± 0.4
Tetra6	-CH_2_-CH(Me)_2_	–22.7 ± 0.5
Tetra7	-CH_2_-CH(Et)(Me)	–23.2 ± 0.4
Tetra8	-CH_2_(*t-*Bu)	–24.1 ± 0.5
Tetra9	-(CH_2_)_2_*t-*Bu	–26.5 ± 0.4
Tetra10	-(CH_2_)_3_*t*-Bu	–36.6 ± 0.6
Tetra11	-(CH_2_)_3_CH(Me)_2_	–27.4 ± 0.5
Tetra12	-(CH_2_)_2_Cy	–30.2 ± 0.4
Tetra13	-CH_2_Cy	–30.8 ± 0.5

a Standard error of mean value.

In particular, all compounds reported in Table 1 were docked in the PCSK9 area depicted by the presence of EGF-A in the X-ray crystal structure (PDB accession code 3GCX(26)). Then, the most probable docking poses, obtained by the GLIDE tool of Maestro software, were additionally investigated by “binding pose metadynamics” (BPMD) simulations,^33^ permitting us to choose the most accurate binding pose (see the Experimental Section for details). Here, the utilization of the BPMD technique enabled us to distinguish the most probable pose (with the lowest PoseScore) from the two best-scored docking solutions for each molecule, allowing for a substantial reduction in the number of MD simulations required for the study (Figure S1, Supporting Information). Consequently, only the best ligand binding pose resulting from the BPMD simulations for each molecule designed was chosen to build the final PCSK9/ligand complexes and then optimized by MD simulations. The obtained trajectory frames were thoroughly examined by visual inspection and by plotting the ligand not-hydrogen atom RMSD vs the simulation time. Subsequently, the frames corresponding to 50 ns of MD simulation length, in which the ligands displayed the lowest conformational freedom in the binding site, were exploited for estimating the ligand binding free-energy values (ΔG*; see the Experimental Section for details) by applying the MM-GBSA approach. Finally, a selected list of compounds endowed with the lowest ΔG* values, together with the best synthetic feasibility and the lowest cost, were rationally selected for synthesis and further biological assays.

### Design of the New Polyimidazole Analogues

In our previous paper, we scored 13 compounds, aiming to optimize the substituents capable of interacting with the negatively charged areas shaped by the PCSK9 residue Asp367. In this attempt, starting from the general tetra-imidazolyl structure of MeIm and aiming to refine the substituent capable of occupying the hydrophobic pocket close to Ile369, Pro155, Ala239, Phe379, and Ala371, 13 new polyimidazoles were designed (Table 1). Then, calculating their ΔG* values, the obtained results suggested that compound **Tetra10,** bearing the -(CH_2_)_3_*t*-Bu group as R_1_, displayed the highest estimated affinity with PCSK9 (Table 1).

Furthermore, to simplify the chemical structure of the compounds and improve the synthetic feasibility of the compounds as well, we tried to fuse the benzene and the first imidazole ring into a naphthalene ring capable of mimicking the π-electron conjugation between the rings. The resulting compounds (**Dim1**; Table 2) displayed an increased predicted binding affinity with PCSK9 since the calculated ΔG* was about 5 kcal/mol lower than **Tetra10** (Table 2). However, the ligand unbound from the enzyme surface within the initial 100 ns of MD simulations. Consequently, to improve the stability of the compound on the PCSK9 surface and to evaluate the influence of the third imidazole ring on the predicted ΔG* of the compounds, we additionally simplified the chemical structure of **Dim1** by displacing the R_1_ group by an H atom (**Dim2**) as well as by electron-rich groups among the classical or non-classical bioisosteres^34,35^ of the imidazole ring. In particular, the presence of alkynes, alkenes, trifluoromethyl, or halogens (**Dim3**–**Dim20**; Table 2) in the chemical structure of the compound was investigated in this series. Interestingly, **Dim2** was stably bound on PCSK9 over 200 ns of MD simulations and displayed a ΔG* value of −35.9 kcal/mol like that of **Tetra10** (−36.6 kcal/mol; Table 1), indicating that the structural simplification did not greatly impact the binding affinity of the compound.

**Table 2:**
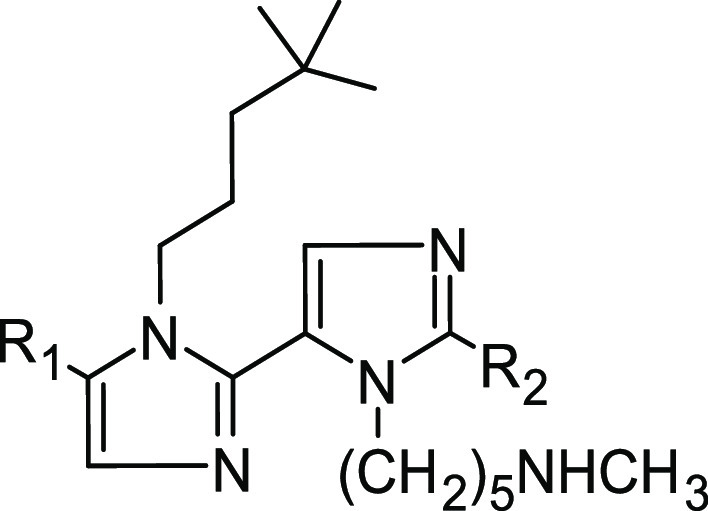
Chemical Structure and Estimated ΔG* Values of the New Series of Dim Compounds Derived from **Tetra10**

compound	R_1_	R_2_	ΔG* ± SE^a^ (kcal/mol)
Tetra10	for comparison, see Table 1		–36.6 ± 0.6
Dim1	naphth-2-yl	*N*^1^-Me-imidazol-5-yl	–41.0 ± 0.3^b^
Dim2	naphth-2-yl	-H	–35.9 ± 0.4
Dim3	naphth-2-yl	-C≡C—H	–38.0 ± 0.4
Dim4	6-Me-naphth-2-yl	-C≡C—H	–40.8 ± 0.5
Dim5	1-OH,6-Me-naphth-2-yl	-C≡C—H	–39.4 ± 0.3
Dim6	6-Me,8-OH-naphth-2-yl	-C≡C—H	–38.6 ± 0.5
Dim7	6,8-diMe-naphth-2-yl	-C≡C—H	–40.0 ± 0.4
Dim8	6-Br-naphth-2-yl	-C≡C—H	–41.9 ± 0.4
Dim9	6-Br-naphth-2-yl	-C≡C—Me	–42.6 ± 0.5
Dim10	6-Br-naphth-2-yl	-C≡C—Et	–39.2 ± 0.3^b^
Dim11	6-Br-naphth-2-yl	-C≡C—CH(Me)_2_	–40.6 ± 0.4
Dim12	6-Br-naphth-2-yl	-*trans*(CH=CH) —Me	–38.6 ± 0.5
Dim13	6-Br-naphth-2-yl	-CF_3_	–38.7 ± 0.3
Dim14	6-Br-naphth-2-yl	-Cl	–38.8 ± 0.3
Dim15	6-Br-naphth-2-yl	-I	–41.0 ± 0.4
Dim16	naphth-2-yl	-I	–39.6 ± 0.2
Dim17	-Ph	-I	–34.6 ± 0.4
Dim18	-Me	-I	–36.5 ± 0.4
Dim19	-Et	-I	–27.9 ± 0.5
Dim20	-*n*-Pr	-I	–31.1 ± 0.5

a Standard error of the mean value.

b Unbound within 100 ns.

In the case of the alkynyl series of compounds (**Dim3**–**Dim8**), the calculated ΔG* values suggested that the applied change was nearly fruitful since a gain in the ΔG* close to 2 kcal/mol was obtained for **Dim3** with respect to **Dim2**. Compounds **Dim4**–**Dim8** were designed to gain an additional advantage in the predicted affinity by decorating the naphthalene ring. Among them, **Dim8**, bearing the 6-Br-napht-2-yl substituent as R_1_, displayed the lowest predicted ΔG* value.

Compounds **Dim9**–**Dim12** were then designed to prove the effect of the R_2_ moiety on **Dim8**, but the obtained results suggested that the R_2_ substituent cannot be greater than the ethynyl. In fact, although **Dim9** showed the lowest predicted ΔG* value, it also showed a high ligand RMSD fluctuation along the MD simulation time (Figure S2A, Supporting Information). Similarly, **Dim10,** bearing a −C_2_Et as R_2_, unbound from the PCSK9 surface within the initial 150 ns of the MD simulations. Conversely, **Dim11** and **Dim12** displayed high stability on the PCSK9 binding site, although their ΔG* values were not lower than those of **Dim8**.

Compounds **Dim13**–**Dim15** were designed to test the effect of the presence of halogens as an R_2_ group on **Dim8**. Remarkably, **Dim15** displayed a ΔG* value very close to that of **Dim8** along with great stability on the PCSK9 surface. Finally, compounds **Dim16**–**Dim20** were designed to investigate the effect of the presence of the 6-Br-napht-2yl substituent on **Dim15**. The obtained results suggested that removal of the Br substituent, as in compound **Dim16**, was not extremely detrimental since a ΔG* value similar to that of **Dim15** was obtained. For **Dim16**, MD simulations were extended to 1300 ns in order to better sample the conformational space of the complex (a plot of RMSD vs simulation time is shown in Figure S2B, Supporting Information), and the obtained results confirmed the high stability and theoretical affinity of the compound on the PCSK9 surface (average RMSD = 1.84 Å, standard deviation = 0.62). Conversely, the ΔG* values calculated for compounds **Dim17**–**Dim20** suggested that a benzene ring or linear alkyl chains as R_1_ in this series of compounds did not lead to compounds more promising than **Dim15** or **Dim16** even if they retained a residual affinity with PCSK9 (Table 2).

### Compound Selection for Synthesis and Biological Evaluation

Considering the results in Table 2, compounds **Dim8** and **Dim15** were considered the most promising since they displayed the lowest predicted ΔG* values. However, early attempts to synthesize them proved unsuccessful due to the incompatibility of the Br substituent with the synthetic sequence. For this reason, given also the small difference in the estimated ΔG* values, **Dim3** and **Dim16** (not containing Br) were chosen for synthesis and biological evaluation. In addition, to experimentally prove the effect of the R_2_ substituent on the biological activity of the compounds, **Dim2**, the simplest derivative containing H as R_2_, was also selected for synthesis. Moreover, since the -CH_2_-Cy resulted second in ranking as an R_1_ moiety in the tetra series (Table 1), we designed and modeled the diimidazole compounds **Dim21**–**Dim23** (Table 3), in which the -CH_2_-Cy replaces the -(CH_2_)_3_-*t*-Bu moiety of compounds **Dim2**, **Dim3**, and **Dim16**. According to their predicted ΔG* values, these compounds were not more promising than **Dim15**, but they were synthesized and biologically evaluated as negative controls for the validation of the applied computational design protocol.

**Table 3:**
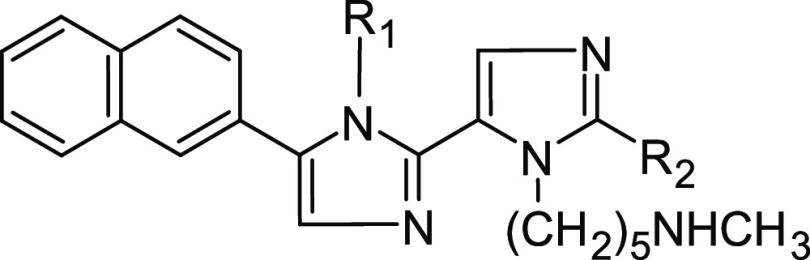
Chemical Structure, Predicted ΔG* Values, PCSK9 Binding Affinity, and HMG-CoAR Inhibitory Activity Obtained for the Compounds Selected for the Synthesis

compound	R_1_	R_2_	ΔG* ± SE^a^ (kcal/mol)	PCSK9/LDLR binding IC_50_ (μM)	HMG-CoAR activity IC_50_ (μM)
Dim2	-(CH_2_)_3_*t*-Bu	-H	–35.9 ± 0.4	1.99 ± 1.65	40.48 ± 15.24
Dim3	- (CH_2_)_3_*t*-Bu	-C≡C—H	–38.0 ± 0.4	0.009 ± 0.01	not active
Dim16	-(CH_2_)_3_*t*-Bu	-I	–39.6 ± 0.2	0.0008 ± 0.001	146.8 ± 75.09
Dim21	-CH_2_Cy	-H	–29.8 ± 0.4	4.50 ± 0.50	38.4 ± 12.71
Dim22	-CH_2_Cy	-I	–32.6 ± 0.5	1.99 ± 2.86	36.21 ± 5.98
Dim23	-CH_2_Cy	-C≡C—H	–33.8 ± 0.5	1.18 ± 1.06	not active

a Standard error of the mean value.

### Chemistry

**Scheme 1:**
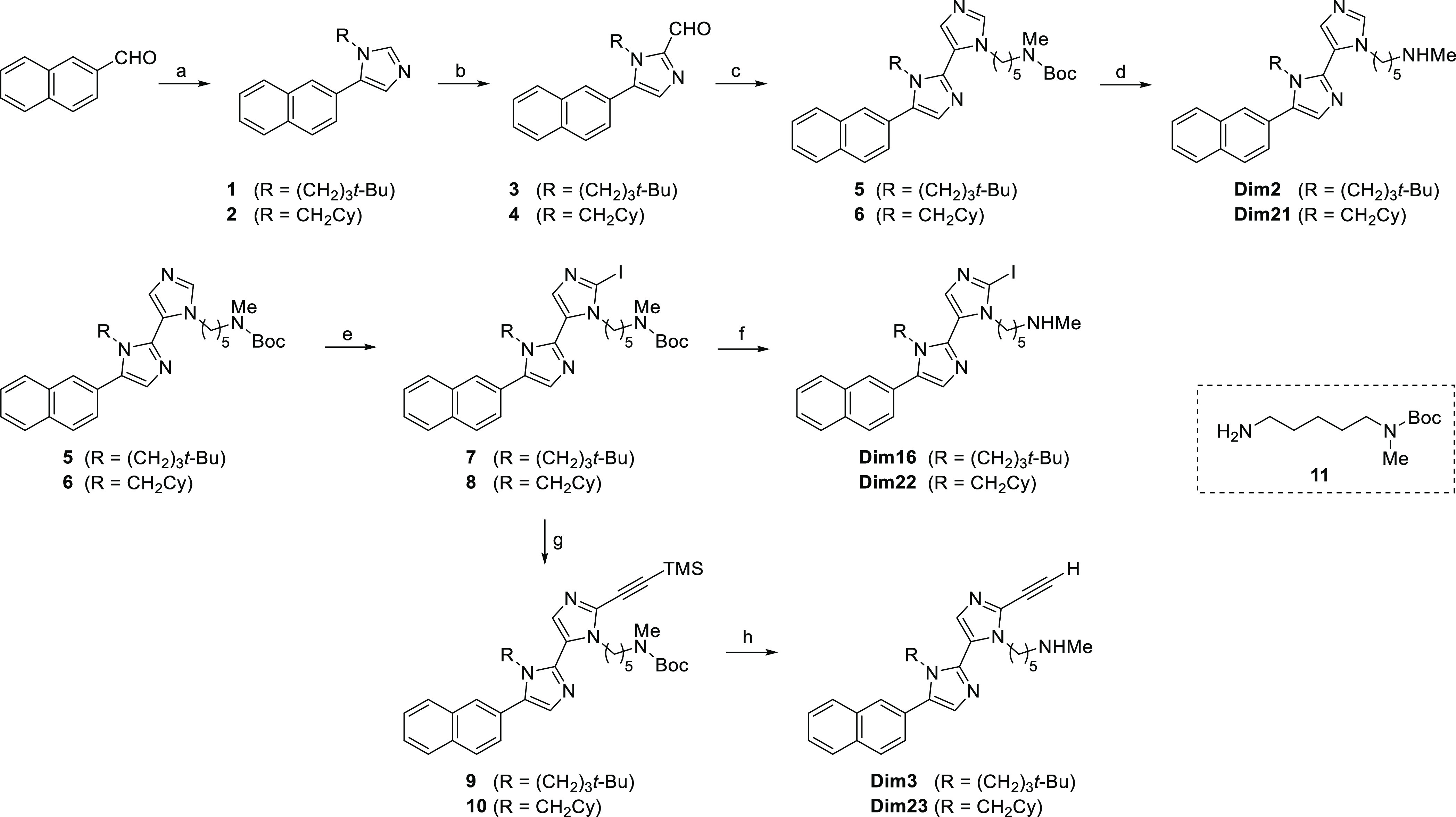
Synthesis of the Target Compounds Reagents and conditions: (a) amine, DMF, 70 °C, 2 h; then TosMIC, K_2_CO_3_, overnight (95% for **1**, 84% for **2**). (b) *n*-BuLi, THF, from −78 to −30 °C, 2 h, and then DMF, rt, overnight (76% for **3**, 77% for **4**). (c) Amine **11**, DMF, 70 °C, 2 h, and then TosMIC, K_2_CO_3_, overnight (66% for **5**, 83% for **6**). (d) 4 N HCl in AcOEt, from 0 °C to rt, 2 h, and then NaHCO_3_/CH_2_Cl_2_ (quant. yield for both **Dim2** and **Dim21**). (e) *n*-BuLi, THF, from −78 to −30 °C, 2 h; then I_2_, rt, overnight (62% for **7**, 75% for **8**). (f) See (d) (quant. yield for both **Dim16** and **Dim22**). (g) Trimethylsilylacetylene, Pd(PPh_3_)_2_Cl_2_, CuI, THF/Et_3_N, 60 °C, 3 h (32% for **9**, 44% for **10**). (h) See (d), and then K_2_CO_3_, MeOH/THF, rt, 2 h (quant. yield for both **Dim3** and **Dim23**).

The six target compounds were synthesized by relying on the twice-repeated van Leusen three-component reaction (vL-3CRs) as the key process (Scheme 1). The vL-3CR generates disubstituted imidazoles in a single step by base-induced condensation between an aldehyde, a primary amine, and tosylmethyl isocyanide (TosMIC). The proper selection of the amine component allows the introduction of the required N-substituent on the imidazole ring. In detail, starting from 2-naphthaldehyde, imidazole derivatives **1** and **2** were obtained in high yields using 4,4-dimethylpentan-1-amine and cyclohexylmethanamine, respectively. A precondensation time of two hours at 70 °C ensured the in situ formation of the intermediate imine, after which TosMIC and K_2_CO_3_ were added. Compounds **1** and **2** were then treated with *n*-BuLi at low temperature and DMF as a formylating agent to give aldehyde derivatives **3** and **4**, respectively, still in good yields. The subsequent vL-3CR employed amine **11** and afforded *N*-Boc-protected diimidazole derivatives **5** and **6** in satisfying yields. From **5** and **6**, the target compounds **Dim2** and **Dim21** can be quantitatively achieved by acidic N-Boc deprotection. On the other hand, treatment of intermediates **5** and **6** with *n*-BuLi and iodine at low temperature afforded iodo derivatives **7** and **8**, which could be *N*-Boc-deprotected to targets **Dim16** and **Dim22**. Finally, Sonogashira coupling between iodo derivatives **7** and **8** and trimethylsilylacetylene afforded alkyne derivatives **9** and **10**, which were easily TMS- and *N*-Boc-deprotected to give the target compounds **Dim3** and **Dim23**. Identity and purity of final compounds, as well as that of all intermediates, were assessed through ^1^H NMR, ^13^C NMR, and high-resolution mass spectrometry.

### Diimidazole Analogues Impair PCSK9–LDLR PPI and HMG-CoAR Activity

**Figure 2:**
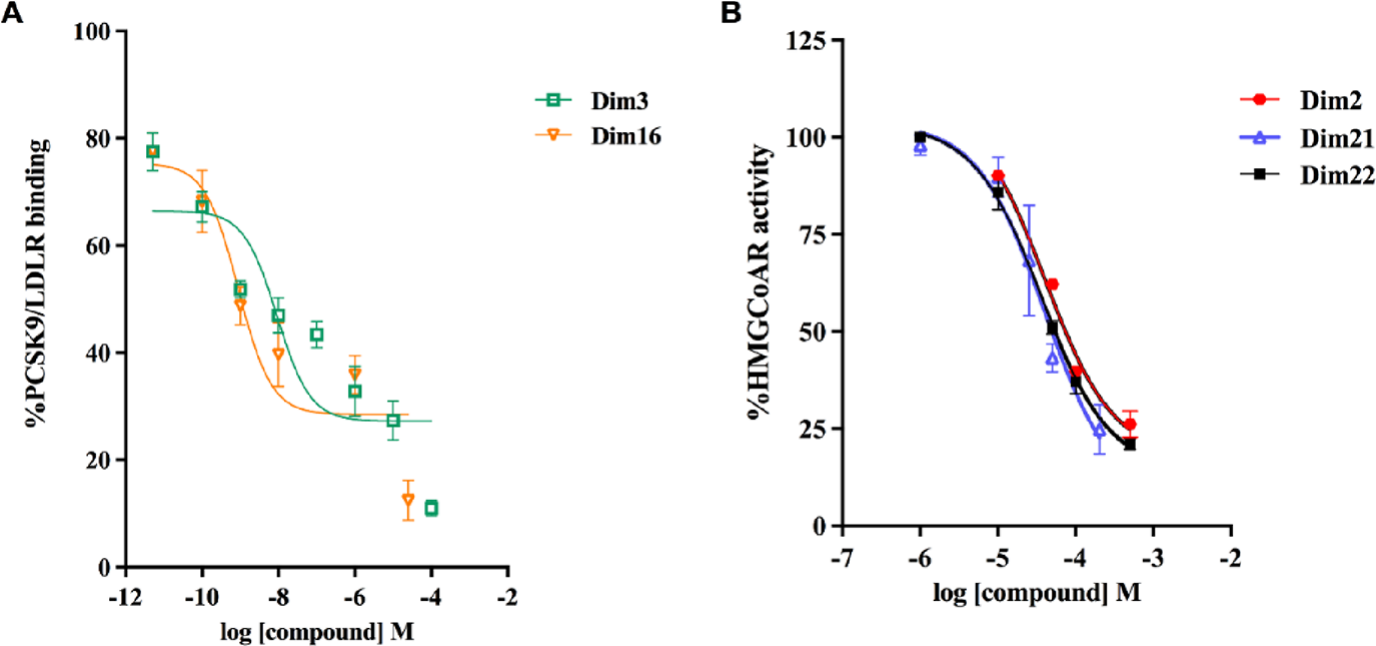
(A) Inhibition of the protein–protein interaction between PCSK9 and LDLR. (B) Inhibition of HMG-CoAR activity. The data points represent the mean ± SD of three independent experiments.

To evaluate the inhibitory ability of the Dim analogues, dedicated experiments were carried out with the aim of verifying whether they can impair the PPI between PCSK9 and LDLR and decrease HMG-CoAR activity. Results indicated that **Dim2**, **Dim3**, **Dim16**, **Dim22**, and **Dim23** reduced PCSK9–LDLR binding with a dose response trend and IC_50_ values of 1.99 ± 1.65, 0.009 ± 0.01, 0.0008 ± 0.001, 1.99 ± 2.86, and 1.18 ± 1.06 μM, respectively (Table 3). The results indicated that **Dim3** and **Dim16** were more active than the other analogues (Figure 2A). However, observing the dose–response curve obtained fitting the data points with a Hill slope equal to −1, it cannot be excluded that the experimental points may possess a biphasic behavior (Figure S3, Supporting Information). In addition, although the data points for **Dim3** and **Dim16** are virtually superimposed at compound concentrations below 10 nM, the fitted IC_50_ is 10-fold different. However, visual inspection of the data points (with no fitting) would not lead one to believe that one compound was 10-fold more potent than the other, suggesting that they may display a more similar potency. Nevertheless, as can be noted comparing the predicted ΔG* and the experimental IC_50_ values, the computational procedure does not accurately rank the compounds. In fact, while **Dim3** and **Dim16** are the most promising by both metrics, **Dim2**, **Dim21**, **Dim22**, and **Dim23** displayed comparable IC_50_ values (1.2–4.5 μM), while their ΔG* values varied by up to 6 kcal/mol. This discrepancy could be due to the missed estimation of the entropic contribution to the Δ*G* (see the Experimental Section for details).

Furthermore, a biochemical investigation was carried out to assess the ability of diimidazole analogues to modulate in vitro HMG-CoAR activity. The results suggested that **Dim2**, **Dim16**, **Dim21**, and **Dim22** inhibited the enzyme with dose–response trends and IC_50_ values of 40.48 ± 15.24, 146.8 ± 75.09, 38.4 ± 12.71, and 36.21 ± 5.98 μM, respectively. Specifically, **Dim2**, **Dim21**, and **Dim22** displayed activity in the micromolar range (Figure 2B), whereas **Dim3** and **Dim23** were not active, as reported in Table 3.

### Effect of Dim3 and Dim16 on HepG2 Cell Vitality

**Figure 3:**
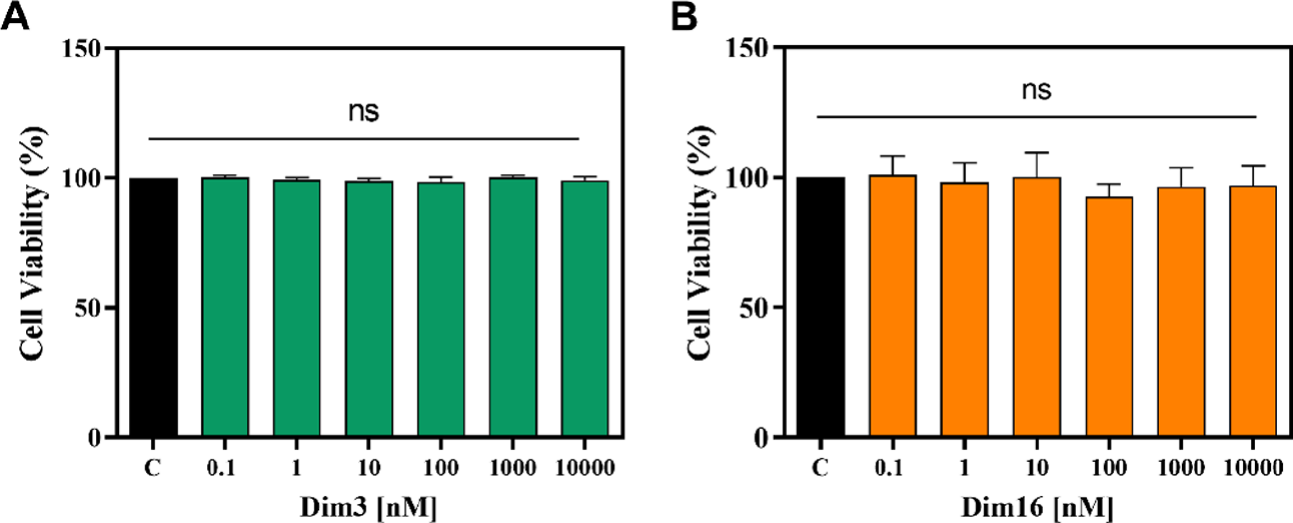
HepG2 cell viability after the **Dim3** (A) and **Dim16** (B) treatments. Bar graphs indicating the results of the cell viability assay of HepG2 cells after **Dim3** and **Dim16** (0.1–10,000 nM) treatment for 48 h. The data points represent the mean ± SD of the three experiments in triplicate. ns: not significant.

Considering that **Dim3** and **Dim16** were the most active compounds inhibiting the PCSK9 ability to bind the LDLR in the biochemical system and that **Dim16** also showed the capability to modulate HMG-CoAR activity, cell-based experiments were conducted with the aim of characterizing the molecular and functional behavior of both Dim analogues using human hepatic HepG2 cells. Hence, preliminary cellular viability experiments (MTT assays) were carried out to exclude any potential effects of treatment with **Dim3** and **Dim16** on the vitality of HepG2 cells. After a 48 h treatment, any reduction in hepatic cell vitality was observed up to 10 μM versus control cells, indicating that **Dim3** and **Dim16** were safe for HepG2 cells in this dose range (Figure 3).

### Diimidazole Analogues Increase the Expression of LDLR Localized on Cellular Membranes

**Figure 4:**
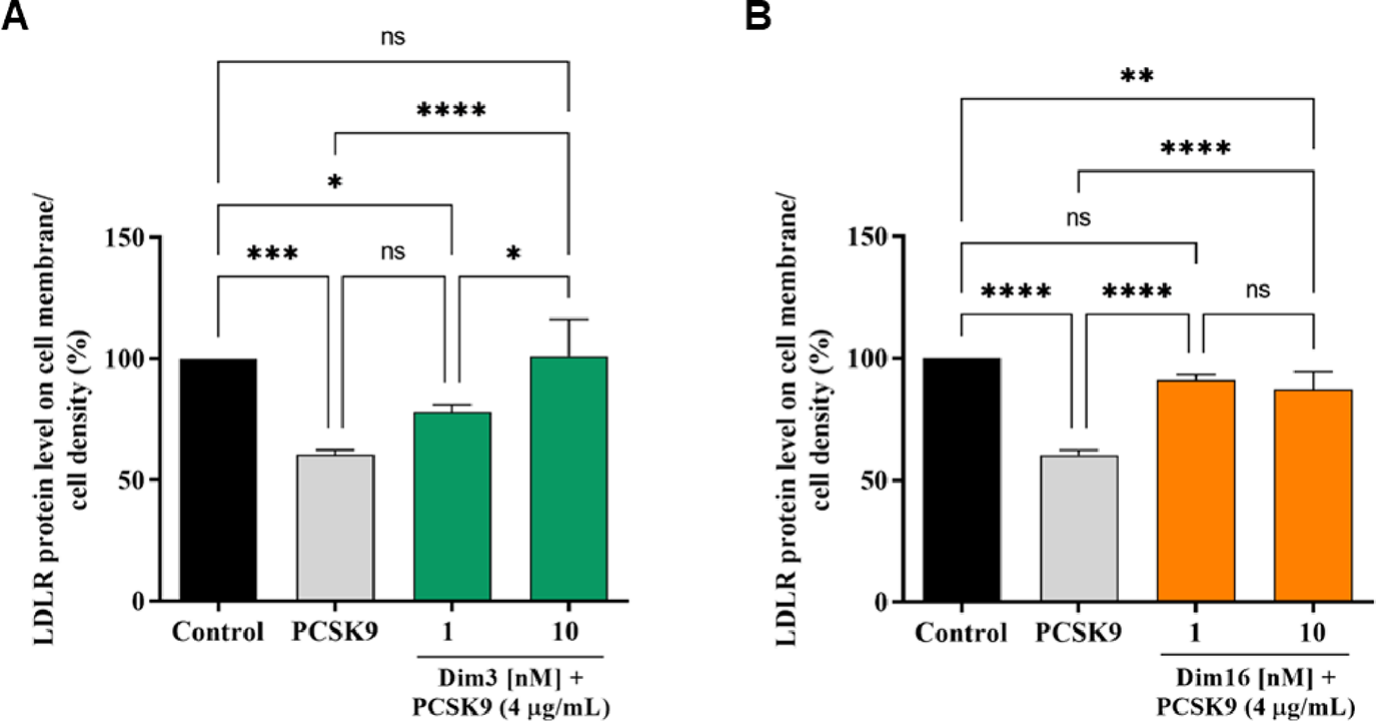
The treatment of HepG2 cells with PCSK9 (4 μg/mL) reduced active LDLR protein levels localized on the surface of cells, which were restored by **Dim3** (A) and **Dim16** (B), inducing an increase in the LDLR protein level on the HepG2 cell surface at 1 nM and 10 nM, respectively. The data points represent the mean ± SD of three independent experiments. *p < 0.05, **p < 0.01, ***p < 0.001, and ****p < 0.00001; ns: not significant.

In addition, the ability of these diimidazole analogues to modulate the levels of LDLR localized on HepG2 surfaces was investigated in the presence of PCSK9 (4 μg/mL). The results indicated that LDLR levels decreased in the presence of PCSK9 alone by 39.71 ± 2.05% compared to untreated control cells, whereas **Dim3** and **Dim16** significantly restored LDLR levels to 77.87 ± 3.04 and 101.1 ± 15.06% (Figure 4A) and 91.1 ± 2.22 and 87.17 ± 7.42% (Figure 4B) when co-incubated with PCSK9 (Figure 4A) at 1 and 10 nM, respectively.

### Diimidazole Analogues Modulate LDL Uptake in HepG2 Cells

**Figure 5:**
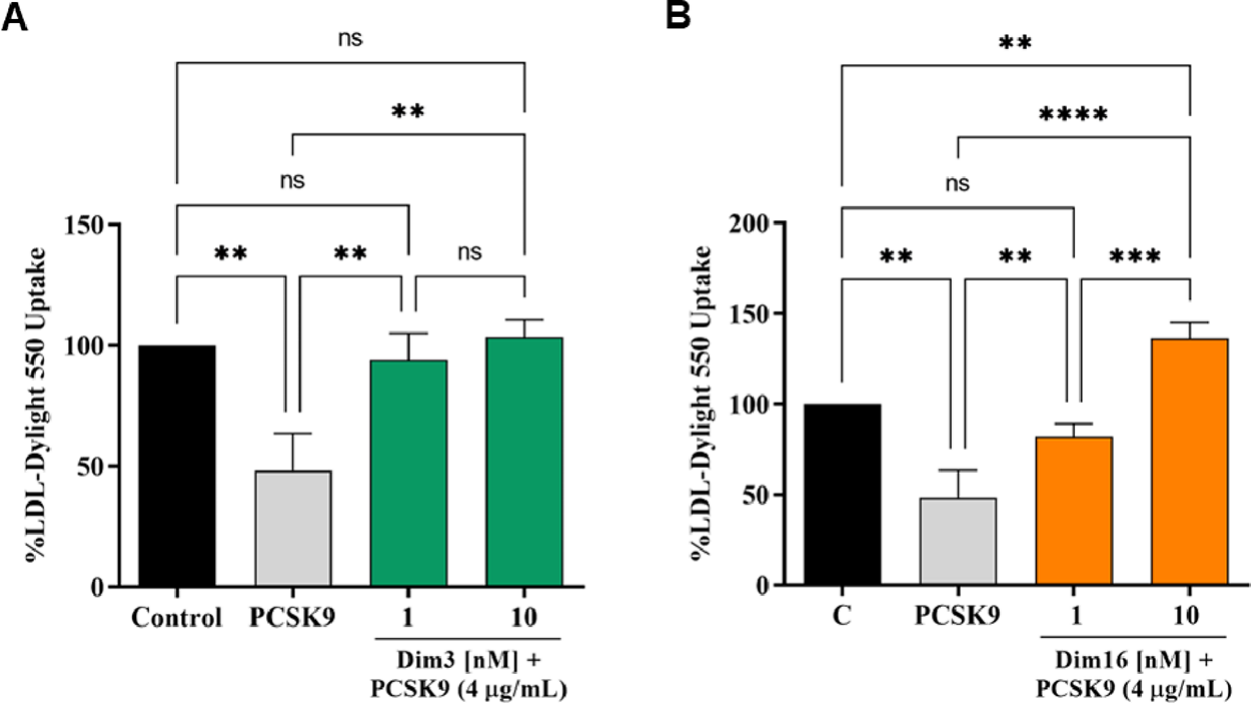
The decreased ability to uptake LDL from the extracellular space by HepG2 cells induced by PCSK9 is prevented by **Dim3** (A) and **Dim16** (B), inducing an improved ability of HepG2 cells to absorb LDL at 1 nM and 10 nM, respectively. The data points represent the mean ± SD of three independent experiments. **p < 0.01, ***p < 0.001, and **** *p* < 0.00001; ns: not significant.

Finally, functional cell assays were performed to verify the capacity of HepG2 cells to uptake extracellular LDL in the presence of PCSK9 (4 μg/mL). HepG2 cells incubated with PCSK9 alone displayed a reduction of 51.69 ± 15.30% in the uptake of fluorescent LDL compared to untreated control cells, indicating reduced LDLR function. After coincubation with PCSK9 at 1 or 10 nM, **Dim3** and **Dim16** restored LDLR function, increasing LDL uptake up to 94.12 ± 10.95 and 103.47 ± 7.34% (Figure 5A) and 81.87 ± 7.45 and 136.47 ± 8.81% (Figure 5B), respectively.

### Docking and MD Simulations on HMG-CoAR

**Figure 6:**
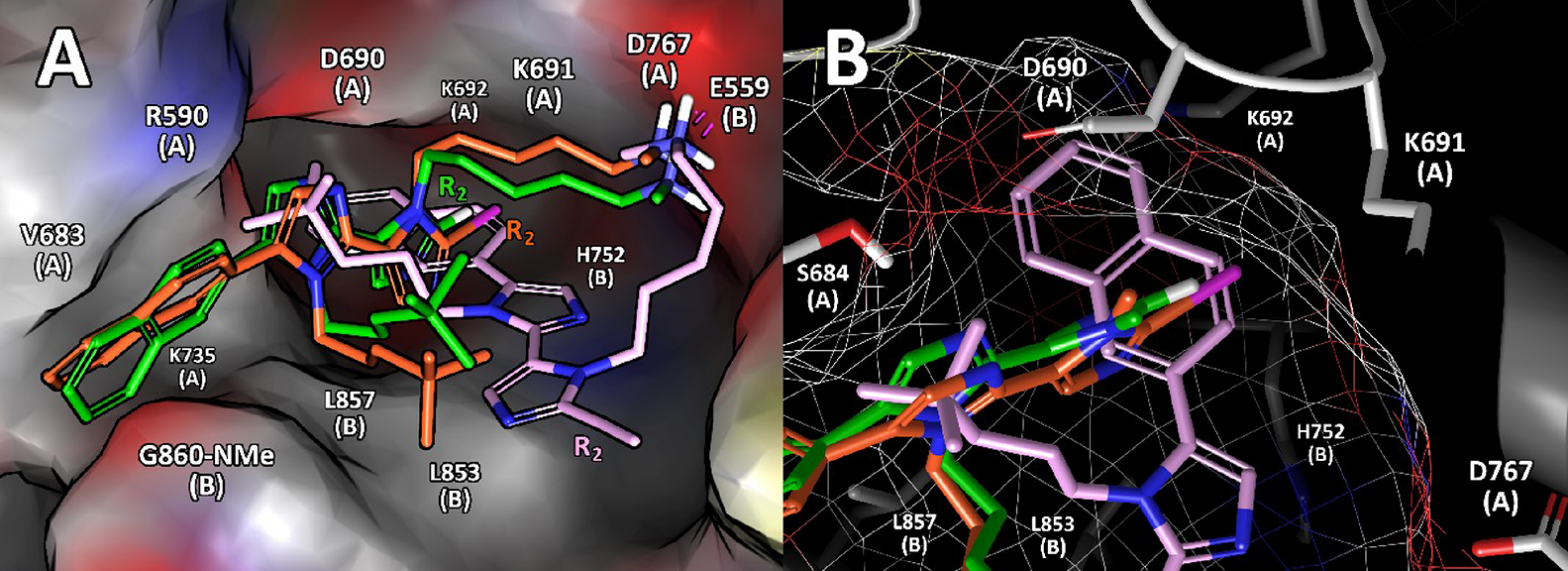
(A) Best docking poses of **Dim2** (green sticks), **Dim16** (orange sticks), and **Dim3** (pink sticks) compounds. The R_2_ group of each compound is labeled. (B) Focused view of the HMG-CoAR area in which the **Dim2** and **Dim16** R_2_ groups are projected. HMG-CoAR, in both panels, is represented as solvent-accessible surface and colored as a standard atom type.

To predict the binding modes of **Dim2**, **Dim3**, and **Dim16** on HMG-CoAR, rationalizing their structure–activity relationships (Table 3), docking calculations and MD simulations were performed. The best docking poses (by *G* score) of the compounds explained how the R_2_ substitution from H (**Dim2**) or I (**Dim16**) to a different group, such as the alkyne of **Dim3**, influenced the predicted binding mode of the compounds (Figure 6). In fact, the H and I atoms of **Dim2** and **Dim16**, respectively, were positioned in a small hydrophobic pocket surrounded by Asp690 (chain A), Lys691 (chain A), Lys692 (chain A), and His752 (chain B). We can suppose that the substitution of the -H or -I atoms by the ethyne group of **Dim3** causes a different, and not productive (in terms of bonds that can be shaped), binding mode (Figure 6B). We do not know if the GLIDE algorithm is so accurate to appreciate a variation of the shape and substituent area of 3.4 Å^2^ (Figure S4, Supporting Information); however the **Dim3**-predicted binding mode was different.

The **Dim2**, **Dim3**, and **Dim16** binding modes predicted by docking calculations were further optimized by performing 250 ns long MD simulations. In these simulations, **Dim2** and **Dim16** remained well anchored in the HMG-CoAR catalytic site for the entire simulation time, showing an average RMSD value of 2.0 Å (SD = 0.3 Å) and 2.8 Å (SD = 0.9 Å), respectively (Figure S4A, Supporting Information). At variance, **Dim3** unbound from the target active site before the first 50 nanoseconds of the MD simulations (Figure S4B, Supporting Information). **Dim2** and **Dim16** displayed calculated ΔG* values of −42.6 ± 0.5 and −37.9 ± 0.6 kcal/mol, respectively, further confirming that our computational data are strongly in line with the experimental HMG-CoAR IC_50_ values reported in Table 3. In fact, **Dim2** showed a lower IC_50_ (40.48 ± 15.24 μM) compared to **Dim16** (146.8 ± 75.09 μM). In addition, **Dim3**, which showed no in vitro inhibitory activity on HMG-CoAR (Table 3), was very unstable during the MD simulations and left the active site of HMG-CoAR after a few steps of MD simulations.

### Preliminary Pharmacokinetic Characterization of Dim22

Since the compound of this series displayed promising activity as hypocholesterolemic agents and, if properly developed, could indeed represent new drug candidates, two pharmacokinetic (PK) parameters, such as water solubility and metabolic stability, were experimentally determined. Experiments were conducted on **Dim22** because it can be considered the cheapest and closest structural analogue of **Dim16**. Indeed, the theoretical prediction of the ADME parameters carried out for both compounds (by Qikprop tool of Maestro) strongly suggested quite similar PK properties (see the Supporting Information). Consequently, the results achieved for **Dim22** highlighted that at pH 7.4, the solubility of the compound was about 10 μM (5.8 μg/mL; Table S2, Supporting Information), a value that can be considered acceptable for a drug active in the low micromolar range. On the other hand, the metabolic stability of **Dim22** was evaluated after the incubation of mouse and human liver microsomes in the presence of uridine diphosphate glucuronic acid (UDPGA) to study the stability of the compound in a glucuronidation phase II reaction. Standards used in the experiments, 7-ethoxycoumarin (7-EC) and 7-ethoxycoumarin (7-OHC), were tested as references for Phase I and II reactions, respectively. The results of these experiments (Table 4 and Tables S1–S3, Supporting Information) highlighted that **Dim22** showed a medium–high clearance with no difference between the two tested species (human and mouse), while the observed metabolism was NADPH-dependent in mice and partially non-NADPH-dependent in humans (50%). The classification of in vitro stability is reported in Table S4 (Supporting Information).

**Table 4:** Phase II Stability in Liver Microsomes of **Dim22** and Standards

	human				mouse			
compound	Cli	SD		SD	Cli	SD		SD
	μL/min/mg protein		min		μL/min/mg protein		min	
**Dim22**	51.5	0.7	26.9	0.4	57.0	2.7	24.3	1.1
7-EC	46.1	0.4	30.0	0.2	290.9	105.4	5.1	1.8
7-OHC	390.0	53.1	3.6	0.5	533.3	105.0	2.7	0.5

### Effect of **Dim16** on Platelet Aggregation

**Figure 7:**
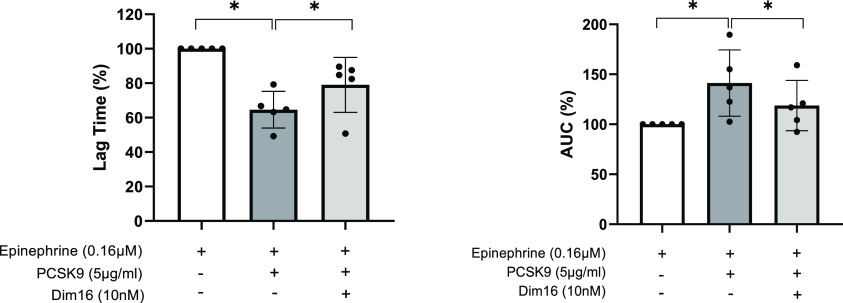
Lag time values (on the left) and area under the curve (AUC, on the right) of platelet aggregation induced by epinephrine (0.16 μM) in the absence (white bar) or presence (gray bar) of PCSK9 (5 μg/mL). The effect of **Dim16** (10 nM) preincubation is reported (light gray bar). Data are expressed as mean ± SD (*n* = 5), setting the lag time and AUC of the epinephrine-stimulated samples as 100.

The effect of PCSK9 inhibition by **Dim16** on platelet function was assessed by light transmission aggregometry on platelet-rich plasma samples from healthy donors (*n* = 5). PCSK9 (5 μg/mL) added to platelet-rich plasma samples significantly potentiated platelet aggregation induced by subthreshold concentrations of epinephrine (0.16 μM), reducing the lag time (∼40%; Figure 7A) and increasing the area under the curve (∼60%; Figure 7B). This effect was significantly prevented by preincubation with 10 nM **Dim16** (Figure 7).

## Discussion

**Figure 8:**
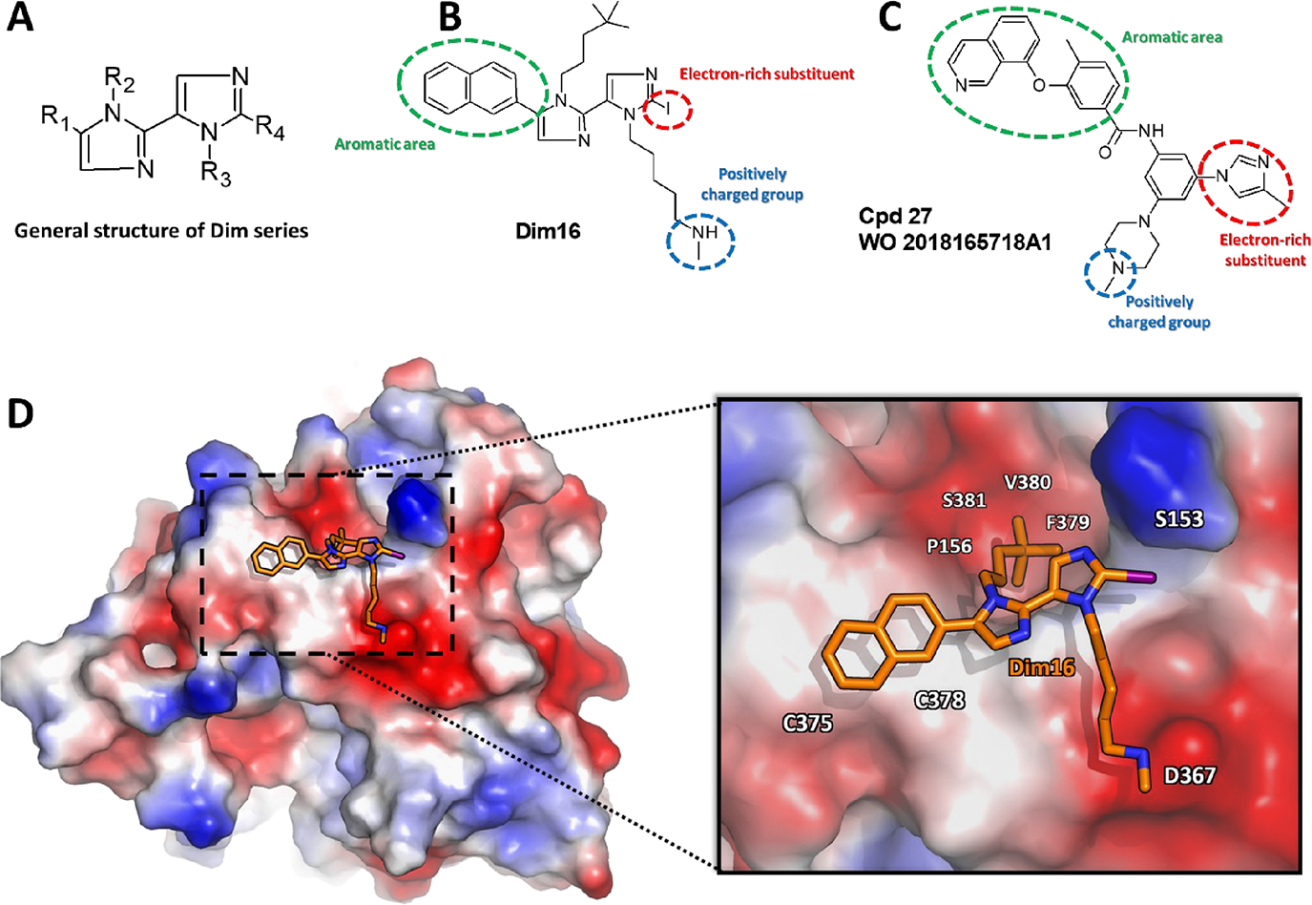
(A) General structure of the Dim series reported in this paper. (B) Molecular formula for **Dim16**. (C) Molecular formula for **Cpd27**, as found in Nyrada patent no. WO2018165718A1. The red, green, and cyan dashed circles depicted in (B) and (C) highlight the **Dim16** and **Cpd27** common structural features. (D) Predicted binding mode of **Dim16** as resulted from docking, metadynamics, and MD simulations. The solvent-accessible surface of PCSK9 is colored depending on the partial charge of the atoms: positive areas are depicted as blue, while red areas suggest the presence of positively charged residues. The carbon atoms of **Dim16** are represented as orange sticks.

In this article, starting from our studies on the simplest tetraimidazole (**MeIm**), we designed new PCSK9 inhibitors endowed with a diimidazole scaffold, which has shown the lowest PCSK9 IC_50_ value (0.8 nM) reported in the literature to date. Considering the theoretical and experimental studies on the series of tetraimidazoles (Table 1), triimidazoles,^25^ and diimidazoles (Figure 8B, Table 2), we can advance the hypothesis that the most potent PCSK9 inhibitors, among those endowed with polyimidazole structures, need at least the following four structural features (Figure 8):

A planar aromatic group capable of interacting with the PCSK9 area shaped by the disulfide-bridged Cys375–Cys378;
A branched alkyl chain capable of filling the hydrophobic PCSK9 pocket sized by Ile369, Pro155, Ala239, Phe379, and Ala371;
An optimal length to reach the PCSK9 negatively charged area close to Asp367;
An electron-rich group, like a halogen or an alkyne substituent.

These features are required to interact with the positively charged amino term of PCSK9 (Ser153), resulting from autocatalytic maturation of the enzyme. All these structural features can be found in the structure of **Dim16**, whose supposed binding mode on the PCSK9 surface is reported in Figure 8D. The absence of one of these structural requirements leads to the compounds being less active on PCSK9. In fact, **Dim2**, which contains H as R_4_, displayed an IC_50_ value 220 times higher than **Dim3**, holding the ethyne as R_4_. In addition, **Dim2** was about 2500 times less active than **Dim16**, which contains I as R_4_. Moreover, the importance of the proper alkyl chain as R_2_ substituent is demonstrated by the data of **Dim21**–**Dim23**. In fact, in all tested compounds, high IC_50_ values were obtained when compared to their analogues, **Dim2**, **Dim3**, and **Dim16**. The importance of the basic chain as R_3_ was discussed in our previous paper.^25^ In these compounds, the optimal distance between the positively charged nucleus and the imidazole core seemed fundamental to designing active compounds. Then again, the aromatic ring as R_1_ seems essential to obtain theoretically active compounds, as demonstrated by the ΔG* values calculated for compounds **Dim17**–**Dim20**. In fact, the presence of the naphthalene ring permitted retention of the activity on PCSK9, permitting the reduction of the synthetic cost of the compounds since it was inserted to mimic the phenyl and first imidazole ring of **Rim13**.

Interestingly, some structural features of **Dim16** can also be found in **Cpd 27** (Figure 1, Nyrada patent). In fact, comparing their chemical structures (Figure 8), it can be easily noted that both compounds contain the following:

A planar skeleton bearing some substituents: the diimidazole scaffold of **Dim16** and the carboxamido-phenyl moiety in **Cpd27**;
The presence of an area rich in aromatic substituents: the naphthyl scaffold of **Dim16** and the isoquinoline moiety of **Cpd27** (green area in Figure 8);
Electron-rich substituents: the iodine atom of **Dim16** (or the ethyne of **Dim2**) and the 3-methyl-imidazole of **Cpd27** (red area in Figure 8);
A positively charged arm: the 5-(*N*-methylamino)-pentyl group of the Dim series and the N_4_-methyl-piperazin-1-yl substituent of Cpd27 (blue area in Figure 8), both protonated at physiologic pH.

Finally, we can suppose that **Dim16**, also bearing an additional branched alkyl chain, such as R_2_, may have all the structural features that justify the low-nanomolar affinity (IC_50_ = 0.8 nM; Table 3).

Nevertheless, the diimidazole series reported in this paper is interesting not only considering their SAR studies in light of the IC_50_ values but also for the remarkable activity displayed by in vitro experiments. In fact, to assess more deeply the molecular and functional effects of PCSK9 inhibition on LDLR pathway modulation, human hepatic HepG2 cells were used. Indeed, HepG2 cells are recognized worldwide as a valuable model for studying hepatocyte functions. Notably, these cells have been shown to express the major enzymes of intra- and extracellular cholesterol metabolism, that is, PCSK9, HMGCoAR, and LDLR.^36^ As clearly shown in Figure 3, both **Dim3** and **Dim16** were safe for this cell line in the range of concentration 0.1 nM to 10 μM. Thus, the cholesterol-lowering activity of both compounds was assessed at the cellular level.

Our findings demonstrate that **Dim3** and **Dim16** possess different functional behaviors in the modulation of cholesterol metabolism. More specifically, through the inhibition of PCSK9/LDLR PPI, both **Dim3** and **Dim16** (1 and 10 nM) restored the active LDLR protein level reduction induced by the incubation of HepG2 cells with PCSK9. These results clearly correlate with the functional ability of both compounds to renew the reduced ability of HepG2 cells to absorb extracellular LDL from the extracellular environment in the same range of concentrations (1 and 10 nM). Overall, these results indicate that both **Dim3** and **Dim16** are 100-fold more active than **Rim13**.^25^ In addition, at the cellular level, **Dim16** is 10-fold more effective than **Dim3** in restoring the LDLR protein level expressed on the surface of human hepatocytes (Figure 4). Unlike **Dim3**, only **Dim16** (10 nM) improved the functional ability of HepG2 cells co-incubated with PCSK9 to uptake LDL compared to untreated cells (Figure 5B). Interestingly, only **Dim16** inhibited both HMG-CoAR activity and PCSK9/LDLR PPI, a peculiar feature that has already been observed by our group. Indeed, we have recently reported on peptide **P5** (LILPKHSDAD), demonstrating that it is capable of inhibiting the PPI between PCSK9 and LDLR, being also one of the most potent food peptides derived from lupin proteins.^14,15^ A molecular docking study allowed the simulation of the effects induced by **P5** on this PPI. The further superimposition of **P5** on the EGF-A domain of LDLR co-crystallized with PCSK9 (PDB accession code 4NE9)^37^ shows good overlapping, justifying the **P5** inhibitory property with an IC_50_ equal to 1.6 μM. In parallel, an experiment demonstrated that **P5** reduced the catalytic activity of HMG-CoAR with an IC_50_ value of 147.2 μM,^14^ and an in silico investigation predicted the potential binding mode to the catalytic site of this enzyme.^38^ Through the inhibition of HMG-CoAR activity, **P5** increases the LDLR protein level in HepG2 cells through the activation of the SREBP-2 transcription factor, and, through down-regulation of HNF-1α, it reduces PCSK9 protein levels and its secretion in the extracellular environment.^14^ This unique synergistic dual inhibitory behavior of **P5** determined the improved ability of HepG2 cells to uptake extracellular LDL with a final hypocholesterolemic effect. In light of these observations, **Dim16** is 2000-fold more active than **P5** in the PCSK9/LDLR PPI, whereas it showed a comparable ability to inhibit HMG-CoAR activity, displaying an IC_50_ equal to 146.8 ± 75.09 μM, clearly suggesting that **Dim16** is the first small molecule endowed with this cholesterol-lowering multitarget activity.

Finally, considering the reported relationship between PCSK9 plasma levels and cardiovascular events^2,39^ and the finding that PCSK9 potentiates platelet aggregation induced by the subthreshold concentration of agonist,^3^ we tested the effect of **Dim16** on platelet aggregation. As shown in Figure 7, our results clearly highlight the capacity of **Dim16** to prevent the potentiating effect of PCSK9 when platelet aggregation is induced by a subthreshold concentration of epinephrine. They also highlight that this pleiotropic effect of PCSK9 occurs through an LDL receptor-dependent mechanism.

Moreover, as a result of the preliminary PK experiments and preparing the samples to conduct the biological assays, the compounds displayed high water solubility, although they displayed medium–high liver clearance in humans as well as in mouse microsomes. In fact, to improve the PK properties (such as solubility and metabolic stability), the naphthyl moiety could be properly decorated by polar or metabolic-resistant groups. Therefore, additional efforts should be made to improve these properties to make these prototypal structures likely drugs for the treatment of hypercholesterolemia.

## Conclusions

The development of new diimidazole (Dim) derivatives, aiming at improving the MeIm polyimidazole structure for inhibiting PCSK9, were successfully achieved. Their design process was guided by computational methods, and a set of compounds were synthesized and thoroughly assessed for their biological activity (performing biochemical and cellular experiments). **Dim3** and **Dim16** impaired the PCSK9/LDLR PPI in the low nanomolar range, effectively increasing the population of LDL-R on the surface of HepG2 cells, improving also the functional hepatic LDL uptake. In addition, **Dim16** exhibited a dual inhibitory effect, being able to target HMGCoA-R and reinforcing its potential use as an innovative hypocholesterolemic agent. Results also demonstrated that these compounds with promising pharmacokinetics properties exert antiplatelet aggregation activity, suggesting further potential therapeutic applications of the Dim analogues. Hence, overall, the Dim analogues hold promise as a new class of drugs for treating cardiovascular diseases.

## Experimental Section

### PCSK9 Model Setup and Docking Protocol

The computational systems utilized in this study were built starting from the coordinates of the PCSK9/RIm13 complex model previously reported by us.^25,32^ All the compounds reported in this article were created using the Maestro platform (release 2020-4, Schrödinger, LLC, New York, NY). Then, all compounds were docked by the GLIDE tool of Maestro. The receptor grid was centered on the RIm13 molecule, and the inner box and outer box dimensions were set to 20 and 30 Å, respectively. Extra precision (XP) mode was used for the docking calculation, and only the poses conformationally distinct were generated (the poses with RMSD values of heavy atoms less than 1.5 Å were discarded). The two binding poses acquiring the lowest *G* score were submitted to BPMD simulations, adopting the BPMD protocol available in Maestro (the number of trials per pose was set to 20).

### Binding Pose Metadynamics

In BPMD, the simulating system’s free-energy landscape is sampled by a history-dependent bias on a small set of collective variables (CVs). Then, monitoring the system free-energy values as a function of the CVs variation, the simulating systems explore their free-energy landscapes escaping from the free-energy minima in which they could be trapped. Essentially, the ligand is automatically obliged to move in the binding site, and the observed mobility under the biasing potential is considered indicative of the predicted binding mode stability or instability.^40^ By applying this method, we were able to reliably discriminate between the ligand-binding poses generated with the docking procedure. Only the data of the six molecules synthesized are shown in the Supporting Information (Figure S1).

### MD Simulations

Once the most probable binding pose for each compound was selected, the PCSK9/ligand complex was solvated using the “tleap” module of AMBER21.^41^ The atomic partial charges of ligands were calculated by the RESP procedure^42^ by using “antechamber” to create the prep file needed to build the topology file for AMBER21^41^ MD simulations. MD simulations (250 ns long) were performed using the pmemd.cuda module of AMBER21^41^ for each ligand/protein complex. The applied protocol and parameters for the MD simulations were the ones reported in our previous papers.^9,27^

### MM-GBSA Binding Free-Energy Calculations

MM-GBSA calculations were performed to compute the ligand binding free-energy values considering the MD frames belonging to the cluster of PCSK9/ligand conformations in which the ligand displayed the highest stability on the enzyme (the RMSD/time plot of the non-hydrogen atoms was considered). The MMPBSA.py module^18^ of AMBER21^41^ was used to accomplish the MM-GBSA calculations, keeping all parameters in the default values. In these calculations, the single trajectory approach was applied, and the entropy contributions to the binding free energy were neglected. For this reason, the estimated binding free-energy values are termed “ΔG*” and not “Δ*G*”.^1,9,19^

### HMG-CoAR Model Setup and MD Simulations

For simplicity, we have selected only the functionally active homodimer (i.e., chains A and B) of the entire homotetramer HMG-CoAR structure solved by X-ray crystallography, available in the “Protein Data Bank” with the PDB ID code 3CCZ,^21^ for the computational analyses. The system was prepared and minimized using the default settings of the “Protein Preparation Wizard” tool, which is available in the Maestro software package (release 2020-4). The ligand docking of **Dim2**, **Dim3**, and **Dim16** was performed using the GLIDE software^22^ implemented in Maestro. The XP mode was applied in these calculations, setting as the center of the grid the centroid of the statin found in the chain A (residue code 5HI), which was co-crystallized in the catalytic site of the HMG-CoAR protein. Only the **Dim2** and **Dim16** poses, endowed with the best-predicted *G* score, were selected for the subsequent 250 ns long MD simulation applying the AMBER21^41^ protocol previously described for the PCSK9/ligand complexes. The statin present in the chain B was kept in its original position to prevent any conformational distortion of the protein that could be induced by the absence of the ligand. The binding free energy estimation was performed considering the last 200 ns of the MD simulations, applying the MM-GBSA calculations available in the AMBER21 package.^41^

### Compound Synthesis

#### General Methods

All commercial materials and solvents (>95% purity grade) were used without further purification. Solvents were purchased as “anhydrous” and used without further purification. All reactions were monitored by thin-layer chromatography on precoated silica gel 60F254; spots were visualized with UV light or by treatment with a 1% aqueous KMnO_4_ solution or 0.2% ninhydrin solution in ethanol. Products were purified by flash chromatography (FC) on silica gel 60 (230–400 mesh) or gravimetric column chromatography on silica gel (60 mesh). NMR spectra were recorded on 300 or 400 MHz Bruker spectrometers. ^1^H NMR and ^13^C NMR chemical shifts were reported in parts per million (ppm) downfield from tetramethylsilane. For ^13^C NMR, the APT pulse sequence was adopted. Coupling constants (*J*) were reported in hertz (Hz). The residual solvent peak was used as an internal reference: ^1^H NMR (CDCl_3_ 7.26 ppm), ^13^C NMR (CDCl_3_ 77.16 ppm). Multiplicities in ^1^H NMR are reported as follows: s = singlet, d = doublet, t = triplet, m = multiplet, br = broad signal. The mass spectra were obtained in ESI positive mode ((+)-HRESIMS) using a Waters Micromass Q-Tof micro mass spectrometer. The purity of the compounds (>95%) was established by elemental analysis.

##### 1-(4,4-Dimethylpentyl)-5-(naphthalen-2-yl)-1*H*-imidazole (**1**)

Under nitrogen, in a flame-dried round bottom flask, 2-naphthaldehyde (515 mg, 3.3 mmol, 1 eq) was dissolved in dry DMF (3.3 mL, 1 M). 4,4-Dimethylpentan-1-amine (760 mg, 6.6 mmol, 2 eq) was added, and the resulting mixture was heated at 70 °C and stirred for 2 h. Potassium carbonate (684 mg, 4.9 mmol, 1.5 eq) and tosylmethyl isocyanide (773 mg, 4.0 mmol, 1.2 mmol) were added sequentially, and the reaction was stirred for an additional 2 h at 70 °C. Tosylmethyl isocyanide (773 mg, 4.0 mmol, 1.2 mmol) was added again, and the reaction was stirred at 70 °C for 12 h. The reaction was cooled to room temperature and then partitioned between ethyl acetate/water. The aqueous phase was extracted with ethyl acetate (×3), and then the combined organic phases were washed with brine (×5), dried over Na_2_SO_4_, and concentrated under reduced pressure to give a residue that was purified by flash chromatography (dichloromethane/methanol 99:1). The purified product was obtained with a 95% yield as a brown foamy solid (yield 95%). ^1^H NMR (400 MHz, CDCl_3_): δ 7.92–7.83 (m, 4H), 7.64 (d, *J* = 1.1 Hz, 1H), 7.55–7.51 (m, 2H), 7.48 (dd, *J* = 8.4, 1.7 Hz, 1H), 7.17 (d, *J* = 1.1 Hz, 1H), 4.01 (t, *J* = 7.3 Hz, 2H), 1.66–1.58 (m, 2H), 1.09–1.04 (m, 2H), 0.78 (s, 9H). ^13^C NMR (100 MHz, CDCl_3_): δ 138.2, 133.3 (Cq), 132.9 (Cq), 132.7 (Cq), 128.4 (2C), 128.0, 127.8, 127.7, 127.6 (Cq), 126.62, 126.58, 126.5, 46.3, 40.6, 30.0 (Cq), 29.1 (3C), 26.2 HRMS (ESI) *m/z*: [M + H]^+^ calcd for C_20_H_25_N_2_ 293.2018; found 293.2029.

##### 1-(Cyclohexylmethyl)-5-(naphthalen-2-yl)-1*H*-imidazole (**2**)

Under nitrogen, in a flame-dried round bottom flask, 2-naphthaldehyde (1 g, 6.4 mmol, 1 eq) was dissolved in dry DMF (6.4 mL, 1 M). Cyclohexylmethanamine (1.7 mL, 12.8 mmol, 2 eq) was added, and the resulting mixture was heated at 70 °C and stirred for 2 h. Potassium carbonate (1.33 g, 9.6 mmol, 1.5 eq) and tosylmethyl isocyanide (1.5 g, 7.68 mmol, 1.2 mmol) were added sequentially, and the reaction was stirred for an additional 2 h at 70 °C. Tosylmethyl isocyanide (1.5 g, 7.68 mmol, 1.2 eq) was added again, and the reaction was stirred at 70 °C for 12 h. The reaction was cooled to room temperature and then partitioned between ethyl acetate/water. The aqueous phase was extracted with ethyl acetate (×3), and then the combined organic phases were washed with brine (×5), dried over Na_2_SO_4_, and concentrated under reduced pressure to give a residue that was purified by flash chromatography (dichloromethane/methanol 99:1 to 98:2). The purified product was obtained as a brown foamy solid (yield 84%). ^1^H NMR (400 MHz, CDCl_3_): δ 7.93–7.85 (m, 4H), 7.58 (s, 1H), 7.54 (dd, *J* = 8.8, 4.8 Hz, 2H), 7.49 (dd, *J* = 8.4, 1.4 Hz, 1H), 7.17 (s, 1H), 3.89 (d, *J* = 7.0 Hz, 2H), 1.67–1.49 (m, 7H), 1.14–1.03 (m, 2H), 0.85–0.76 (m, 2H). ^13^C NMR (100 MHz, CDCl_3_): δ 139.5, 134.0 (2 Cq), 133.9 (Cq), 133.4 (Cq) 129.3, 129.1, 128.7, 128.4 (2C), 127.3, 127.2, 127.1, 52.4, 39.7, 31.2 (2C), 26.7, 26.1 (2C) HRMS (ESI) *m/z*: [M + H]^+^ calcd for C_20_H_23_N_2_ 291.1861; found 291.1855.

##### 1-(4,4-Dimethylpentyl)-5-(naphthalen-2-yl)-1*H*-imidazole-2-carbaldehyde (**3**)

In a flame-dried round bottom flask, compound **1** (1.18 g, 4.0 mmol, 1 eq) was dissolved in dry THF (8 mL, 0.5 M) under a nitrogen atmosphere and cooled to −78 °C. An *n*-butyl lithium solution (1.6 M in hexane, 5 mL, 8.0 mmol, 2 eq) was added dropwise, and the resulting mixture was stirred for 2 h, during which the temperature was slowly raised up to −30 °C. The reaction was cooled to −78 °C, then dimethylformamide (0.6 mL, 8.0 mmol, 2 eq) was added, and the reaction was slowly warmed up to room temperature and stirred for 12 h. The resulting mixture was quenched with distilled water and extracted with ethyl acetate (×2). The combined organic phases were washed with brine (×3), dried over Na_2_SO_4_, and concentrated under reduced pressure to give a residue that was purified by flash chromatography (dichloromethane/ethyl acetate 97:3). The purified product was obtained as a brown foamy solid (yield 76%). ^1^H NMR (400 MHz, CDCl_3_): δ 9.91 (s, 1H), 7.98 (d, *J* = 8.4 Hz, 1H), 7.95–7.89 (m, 3H), 7.61–7.59 (m, 2H), 7.50 (d, *J* = 8.4 Hz, 1H), 7.43 (s, 1H), 4.41 (t, *J* = 7.6 Hz, 2H), 1.74–1.66 (m, 2H), 1.10–1.05 (m, 2H), 0.81 (s, 9H). ^13^C NMR (100 MHz, CDCl_3_): δ 182.6, 144.8 (Cq), 140.2 (Cq), 133.9 (Cq), 133.8 (Cq), 132.2, 129.5 (2C), 128.9, 128.5, 127.9, 127.7, 126.9, 126.3 (Cq), 46.8, 41.0, 30.3 (Cq), 29.8 (3C), 27.2 HRMS (ESI) *m/z*: [M + H]^+^ calcd for C_21_H_25_N_2_O 321.1967; found 321.1951.

##### 1-(Cyclohexylmethyl)-5-(naphthalen-2-yl)-1*H*-imidazole-2-carbaldehyde (**4**)

In a flame-dried round bottom flask, compound **2** (2.32 g, 8.0 mmol, 1 eq) was dissolved in dry THF (16 mL, 0.5 M) under nitrogen atmosphere and cooled to −78 °C. *n*-Butyl lithium solution (1.6 M in hexane, 10 mL, 16.0 mmol, 2 eq) was added dropwise, and the resulting mixture stirred for 2 h, during which the temperature was slowly raised up to −30 °C. The reaction was cooled to −78 °C, then dimethylformamide (1.24 mL, 16.0 mmol, 2 eq) was added, and the reaction was slowly warmed up to room temperature and stirred for 12 h. The resulting mixture was quenched with distilled water and extracted with ethyl acetate (×2). The combined organic phases were washed with brine (×3), dried over Na_2_SO_4_, and concentrated under reduced pressure to give a residue that was purified by flash chromatography (dichloromethane/ethyl acetate 98:2 to 97:3). The purified product was obtained as a brown foamy solid (yield 77%). ^1^H NMR (400 MHz, CDCl_3_): δ 9.89 (s, 1H), 7.99–7.90 (m, 4H), 7.61–7.59 (m, 2H), 7.49 (dd, *J* = 8.4, 1.6 Hz, 1H), 7.41 (s, 1H), 4.42 (d, *J* = 7.4 Hz, 2H), 1.59–1.50 (m, 5H), 1.41–1.37 (m, 2H), 1.05–0.94 (m, 2H), 0.72–0.63 (m, 2H). ^13^C NMR (101 MHz, CDCl_3_): δ 182.8, 145.3 (Cq), 140.7 (Cq), 133.9 (Cq), 133.8 (Cq), 132.5, 129.6, 129.5, 128.9, 128.5, 127.8, 127.6, 127.0, 126.7 (Cq), 51.6, 39.9, 30.8 (2C), 26.6, 26.1 (2C) HRMS (ESI) *m/z*: [M + Na]^+^ calcd for C_21_H_22_N_2_NaO 341.1630; found 341.1646.

##### *tert*-Butyl-(5-(1-(4,4-dimethylpentyl)-5-(naphthalen-2-yl)-1*H*,3′*H*-[2,4′-biimidazol]-3′-yl)pentyl)(methyl)carbamate (**5**)

Under nitrogen and in a flame-dried round bottom flask, aldehyde **3** (973 mg, 3.0 mmol, 1 eq) was dissolved in dry DMF (3 mL, 1 M). Amine **11** (789 mg, 3.7 mmol, 1.2 eq) was added, and the resulting mixture was heated at 70 °C and stirred for 2 h. Potassium carbonate (630 mg, 4.5 mmol, 1.5 eq) and tosylmethyl isocyanide (713 mg, 3.7 mmol, 1.2 eq) were added sequentially, and the reaction stirred for an additional 2 h at 70 °C. Tosylmethyl isocyanide (713 mg, 3.7 mmol, 1.2 eq) was added again, and the reaction was stirred at 70 °C for 12 h. The reaction was cooled to room temperature and then partitioned between ethyl acetate/water. The aqueous phase was extracted with ethyl acetate (×3), and the combined organic phases were washed with brine (×5), dried over Na_2_SO_4_, and concentrated under reduced pressure to give a residue that was purified by flash chromatography (dichloromethane/methanol 98:2 to 96:4). The purified product was obtained as a dark brown foamy solid (yield 66%). ^1^H NMR (400 MHz, CDCl_3_): δ 7.97–7.89 (m, 4H), 7.72 (s, 1H), 7.59–7.54 (m, 3H), 7.32 (s, 1H), 7.29 (s, 1H), 4.28 (t, *J* = 7.4 Hz, 2H), 4.12 (t, *J* = 7.6 Hz, 2H), 3.20 (t, *J* = 6.4 Hz, 2H), 2.84 (s, 3H), 1.81–1.75 (m, 2H), 1.58–1.51 (m, 2H), 1.47 (s, 9H), 1.42–1.30 (m, 4H), 0.91–0.87 (m, 2H), 0.68 (s, 9H). ^13^C NMR (100 MHz, CDCl_3_): δ 156.4 (Cq), 139.4, 139.3 (Cq), 134.9 (Cq), 134.0 (2Cq), 133.5 (Cq), 130.3, 129.5, 129.2, 128.7 (2C), 128.5, 127.4, 127.3 (2C), 122.9 (Cq), 79.9 (Cq), 48.9, 46.7, 46.4, 41.0, 34.8, 31.4, 30.6 (Cq), 29.7 (3C), 29.2 (3C), 27.8, 26.8, 24.4 HRMS (ESI) *m/z*: [M + H]^+^ calcd for C_34_H_48_N_5_O_2_ 558.3808; found 558.3817.

##### tert-Butyl-(5-(1-(cyclohexylmethyl)-5-(naphthalen-2-yl)-1*H*,3′*H*-[2,4′-biimidazol]-3′-yl)pentyl)(methyl)carbamate (**6**)

Under nitrogen, in a flame-dried round bottom flask, aldehyde **4** (306 mg, 0.96 mmol, 1 eq) was dissolved in dry DMF (2 mL, 0.5 M). Amine **11** (249 mg, 1.15 mmol, 1.2 eq) was added, and the resulting mixture was heated at 70 °C and stirred for 2 h. Potassium carbonate (199 mg, 1.44 mmol, 1.5 eq) and tosylmethyl isocyanide (224.9 mg, 1.15 mmol, 1.2 eq) were added sequentially, and the reaction was stirred for additional 2 h at 70 °C. Tosylmethyl isocyanide (244.9 mg, 1.15 mmol, 1.2 eq) was added again, and the reaction was stirred at 70 °C for 12 h. The reaction was cooled to room temperature and then partitioned between ethyl acetate/water. The aqueous phase was extracted with ethyl acetate (×3), and the combined organic phases were washed with brine (×5), dried over Na_2_SO_4_, and concentrated under reduced pressure to give a residue that was purified by flash chromatography (dichloromethane/methanol 98:2 to 95:5). The purified product was obtained as a dark brown foamy solid (yield 83%). ^1^H NMR (300 MHz, CDCl_3_): δ 7.95–7.88 (m, 4H), 7.78 (s, 1H) 7.58–7.49 (m, 3H), 7.34 (s, 1H), 7.25 (s, 1H), 4.29 (t, *J* = 7.2 Hz, 2H), 4.05 (d, *J* = 7.1 Hz, 2H), 3.18 (t, *J* = 6.9 Hz, 2H), 2.81 (s, 3H), 1.82–1.72 (m, 2H), 1.55–1.52 (m, 2H), 1.49 (s, 9H), 1.48–1.24 (m, 7H), 0.89–0.75 (m, 2H), 0.57–0.46 (m, 2H). ^13^C NMR (100 MHz, CDCl_3_): δ 156.5 (Cq), 140.0 (Cq), 139.4, 135.6 (Cq), 134.1 (2Cq), 133.4 (Cq), 130.6, 129.5, 129.3, 128.8, 128.6, 128.5, 127.3, 127.2 (2C), 123.2 (Cq), 79.9 (Cq), 52.1, 49.1, 46.6, 39.0, 34.9, 31.3, 30.7, 30.4, 29.2 (3C), 27.9, 26.5, 26.0 (2C), 24.5 HRMS (ESI) *m/z*: [M + Na]^+^ calcd for C_34_H_45_N_5_NaO_2_ 578.3471; found 578.3462.

##### 5-(1-(4,4-Dimethylpentyl)-5-(naphthalen-2-yl)-1*H*,3′*H*-[2,4′-biimidazol]-3′-yl)-*N*-methylpentan-1-amine (**Dim2**)

Under nitrogen, in a flame-dried round bottom flask, compound **5** (48 mg, 0.10 mmol, 1 eq) was dissolved in dry ethyl acetate (1 mL, 0.1 M) and cooled to 0 °C. HCl solution (4 N in ethyl acetate, 0.5 mL, 2 mmol, 20 eq) was added dropwise at 0 °C, and then the reaction was allowed to warm up to room temperature and stirred for 2 h. The reaction mixture was concentrated under reduced pressure, and the product was partitioned between saturated aqueous NaHCO_3_ and CH_2_Cl_2_. The organic phase was dried over Na_2_SO_4_ and concentrated under reduced pressure to afford pure **Dim2** as a dark brown solid (yield >99%). ^1^H NMR (400 MHz, CDCl_3_): δ 7.95–7.87 (m, 4H), 7.67 (s, 1H), 7.56–7.53 (m, 3H), 7.29–7.27 (m, 2H), 4.26 (t, *J* = 7.2 Hz, 2H), 4.10 (t, *J* = 7.5 Hz, 2H), 2.62 (t, *J* = 6.6 Hz, 2H), 2.46 (s, 3H), 1.79–1.75 (m, 2H), 1.60–1.52 (m, 2H), 1.45–1.37 (m, 4H), 0.90–0.86 (m, 2H), 0.67 (s, 9H). ^13^C NMR (10 MHz, CDCl_3_): δ 138.9 (2Cq), 134.2 (Cq), 133.3 (2Cq), 132.9 (Cq), 128.8, 128.5, 128.0, 127.9, 127.8, 126.7, 126.6 (3C), 122.3 (Cq), 51.3, 45.8, 45.7, 40.3, 35.9, 30.8, 29.7 (Cq), 29.0 (3C), 28.7, 26.1, 24.2 HRMS (ESI) *m/z*: [M + H]^+^ calcd for C_29_H_40_N_5_ 458.3284; found 458.3275. Anal. calcd. for C_29_H_39_N_5_: C, 76.11; H, 8.59; N, 15.30; found: C, 76.35; H, 8.50; N, 15.16.

##### 5-(1-(Cyclohexylmethyl)-5-(naphthalen-2-yl)-1*H*,3′*H*-[2,4′-biimidazol]-3’-yl)-*N*-methylpentan-1-amine (**Dim21**)

Under nitrogen, in a flame-dried round bottom flask, compound **6** (108 mg, 0.194 mmol, 1 eq) was dissolved in dry ethyl acetate (1 mL, 0.2 M) and cooled to 0 °C. HCl solution (4 N in ethyl acetate, 1 mL, 4 mmol, 20 eq) was added dropwise at 0 °C, and then the reaction was allowed to warm up to room temperature and stirred for 2 h. The reaction mixture was concentrated under reduced pressure, and the product was partitioned between saturated aqueous NaHCO_3_ and CH_2_Cl_2_. The organic phase was dried over Na_2_SO_4_ and concentrated under reduced pressure to afford pure **Dim21** as a dark brown solid (yield >99%). ^1^H NMR (400 MHz, CDCl_3_): δ 7.96–7.84 (m, 6H), 7.57–7.50 (m, 3H), 7.28 (m, 1H), 5.74 (br s, 1H), 4.25 (t, *J* = 7.1 Hz, 2H), 4.06 (d, *J* = 7.0 Hz, 2H), 2.74 (t, *J* = 6.9 Hz, 2H), 2.53 (s, 3H), 1.84–1.76 (m, 2H), 1.68–1.60 (m, 2H), 1.53–1.40 (m, 7H), 0.94–0.86 (m, 4H), 0.59–0.50 (m, 2H). ^13^C NMR (100 MHz, CDCl_3_): δ 135.0 (Cq), 133.4 (2Cq), 132.8 (2Cq), 129.7, 128.6 (2C), 128.1 (2C), 127.9, 127.8, 126.7, 126.6, 126.5, 123.5 (Cq), 51.4, 50.5, 45.7, 38.3, 34.9, 30.5, 30.1 (2C), 27.5, 25.9, 25.4 (2C), 24.0 HRMS (ESI) *m/z*: [M + H]^+^ calcd for C_29_H_38_N_5_ 456.3127; found 456.3133. Anal. calcd. for C_29_H_37_N_5_: C, 76.44; H, 8.19; N, 15.37; found: C, 76.22; H, 8.35; N, 15.02.

##### *tert*-Butyl-(5-(1-(4,4-dimethylpentyl)-2′-iodo-5-(naphthalen-2-yl)-1*H*,3′*H*-[2,4′-biimidazol]-3′-yl)pentyl)(methyl)carbamate (**7**)

In a flame-dried round bottom flask, compound **5** (942 mg, 1.7 mmol, 1 eq) was dissolved in dry THF (3.4 mL, 0.5 M) under a nitrogen atmosphere and cooled to −78 °C. *n*-Butyl lithium solution (1.6 M in hexane, 2.1 mL, 3.4 mmol, 2 eq) was added dropwise, and the resulting mixture was stirred for 2 h, during which the temperature was slowly raised up to −30 °C. Iodine (858 mg, 3.4 mmol, 2 eq) was dissolved in the minimum possible amount of dry THF, and then the solution was added to the reaction mixture, slowly warmed up to room temperature, and stirred for 24 h. The reaction was cooled to 0 °C, and the resulting dark mixture was quenched with saturated aqueous NH_4_Cl and extracted with ethyl acetate (×3). The combined organic phases were washed with saturated aqueous Na_2_CO_3_ (×1) and brine (×1), dried over Na_2_SO_4_, and concentrated under reduced pressure to give a residue that was purified by flash chromatography (dichloromethane/ethyl acetate 1:1). The purified product was obtained as a dark orange solid (yield 62%). ^1^H NMR (400 MHz, CDCl_3_): δ 7.98–7.90 (m, 4H), 7.60–7.53 (m, 3H), 7.34–7.33 (m, 2H), 4.25 (t, *J* = 7.7 Hz, 2H), 4.07 (t, *J* = 7.6 Hz, 2H), 3.21 (t, *J* = 7.1 Hz, 2H), 2.85 (s, 3H), 1.77–1.69 (m, 2H), 1.59–1.51 (m, 2H), 1.47 (s, 9H), 1.43–1.32 (m, 4H), 0.91–0.87 (m, 2H), 0.68 (s, 9H). ^13^C NMR (100 MHz, CDCl_3_): δ 156.5 (Cq), 138.3 (Cq), 135.3 (Cq), 134.0, 133.9 (Cq), 133.7 (2Cq), 129.4, 128.9, 128.7 (2C), 128.5, 127.7 (Cq), 127.5 (2C), 127.1, 94.4 (Cq), 79.9 (Cq), 48.9 (2C), 46.6, 41.0, 34.9, 31.0, 30.4 (Cq), 29.7 (3C), 29.2 (3C), 27.9, 26.9, 24.4 HRMS (ESI) *m/z*: [M + H]^+^ calcd for C_34_H_47_IN_5_O_2_ 684.2774; found 684.2783.

##### tert-Butyl-(5-(1-(cyclohexylmethyl)-2′-iodo-5-(naphthalen-2-yl)-1*H*,3′*H*-[2,4′-biimidazol]-3′-yl)pentyl)(methyl)carbamate (**8**)

In a flame-dried round-bottom flask, compound **6** (566 mg, 1.0 mmol, 1 eq) was dissolved in dry THF (2 mL, 0.5 M) under a nitrogen atmosphere and cooled to −78 °C. *n*-Butyl lithium solution (1.6 M in hexane, 0.95 mL, 1.5 mmol, 1.5 eq) was added dropwise, and the resulting mixture was stirred for 2 h, during which the temperature was slowly raised up to −30 °C. Iodine (518 mg, 2.0 mmol, 2 eq) was dissolved in the minimum possible amount of dry THF, and then the solution was added to the reaction mixture, slowly warmed up to room temperature, and stirred for 24 h. The reaction was cooled to 0 °C, and the resulting dark mixture was quenched with saturated aqueous NH_4_Cl and extracted with ethyl acetate (×3). The combined organic phases were washed with saturated aqueous Na_2_CO_3_ (×1) and brine (×1), dried over Na_2_SO_4_, and concentrated under reduced pressure to give a residue that was purified by flash chromatography (dichloromethane/ethyl acetate 1:1). The purified product was obtained as a dark orange solid (yield 75%). ^1^H NMR (400 MHz, CDCl_3_): δ 7.97–7.89 (m, 4H), 7.60–7.52 (m, 3H), 7.37 (s, 1H), 7.29 (s, 1H), 4.28 (t*, J* = 6.7 Hz, 2H), 4.04 (d, *J* = 7.2 Hz, 2H), 3.22 (t, *J* = 6.7 Hz, 2H), 2.86 (s, 3H) 1.75–1.71 (m, 2H), 1.59–1.55 (m, 2H), 1.53–1.50 (m, 2H), 1.48 (s, 9H), 1.38–1.29 (m, 5H), 0.93–0.87 (m, 4H), 0.57–0.54 (m, 2H). ^13^C NMR (100 MHz, CDCl_3_): δ 156.5 (Cq), 139.1 (Cq), 135.9 (Cq), 134.0 (2Cq), 133.8, 133.5 (Cq), 130.4, 129.3, 128.8, 128.7, 128.5, 128.4 (Cq), 127.4, 127.3, 127.1, 94.1 (Cq), 80.0 (Cq), 52.2, 49.4, 48.8, 38.9, 35.0, 31.0, 30.8, 30.4, 29.2 (3C), 28.3, 26.5, 26.0 (2C), 24.5 HRMS (ESI) *m/z*: [M + H]^+^ calcd for C_34_H_45_IN_5_O_2_ 682.2618; found 682.2627.

##### 5-(1-(4,4-Dimethylpentyl)-2′-iodo-5-(naphthalen-2-yl)-1*H*,3′*H*-[2,4′-biimidazol]-3′-yl)-*N*-methylpentan-1-amine (**Dim16**)

Under nitrogen and in a flame-dried round bottom flask, compound **7** (50 mg, 0.07 mmol, 1 eq) was dissolved in dry ethyl acetate (0.35 mL, 0.2 M) and cooled to 0 °C. HCl solution (4 N in ethyl acetate, 0.35 mL, 1.4 mmol, 20 eq) was added dropwise at 0 °C, and then the reaction was allowed to warm up to room temperature and stirred for 2 h. The reaction mixture was concentrated under reduced pressure, and the product was partitioned between saturated aqueous NaHCO_3_ and CH_2_Cl_2_. The organic phase was dried over Na_2_SO_4_ and concentrated under reduced pressure to afford pure **Dim16** as a dark brown solid (yield >99%). ^1^H NMR (400 MHz, CDCl_3_): δ 7.95–7.89 (m, 4H), 7.56–7.53 (m, 3H), 7.31 (m, 2H), 4.15 (t, *J* = 7.1 Hz, 2H), 4.02 (t, *J* = 7.5 Hz, 2H), 2.97 (t, *J* = 6.2 Hz, 2H), 2.66 (s, 3H), 1.92–1.80 (m, 4H), 1.53–1.46 (m, 2H), 1.40–1.35 (m, 2H), 0.88–0.83 (m, 2H), 0.66 (s, 9H). ^13^C NMR (100 MHz, CDCl_3_): δ 138.4 (Cq), 135.4 (Cq), 133.93 (Cq), 133.92 (Cq), 133.6 (Cq), 129.5, 129.3, 128.83, 128.77, 128.5, 127.8 (Cq), 127.4 (2C), 127.2, 126.3 (Cq), 95.1 (Cq), 49.9, 48.4, 46.5, 41.0, 34.1, 30.6 (Cq), 30.2, 29.7 (3C), 26.8, 26.1, 24.2 HRMS (ESI) *m/z*: [M + H]^+^ calcd for C_29_H_39_IN_5_ 584.2250; found 584.2262. Anal. calcd. for C_29_H_38_IN_5_: C, 59.69; H, 6.56; I, 21.75; N, 12.00; found: C, 59.37; H, 6.76; N, 11.89.

##### 5-(1-(Cyclohexylmethyl)-2′-iodo-5-(naphthalen-2-yl)-1*H*,3′*H*-[2,4′-biimidazol]-3′-yl)-*N*-methylpentan-1-amine (**Dim22**)

Under nitrogen, in a flame-dried round bottom flask, compound **8** (50 mg, 0.07 mmol, 1 eq) was dissolved in dry ethyl acetate (0.35 mL, 0.2 M) and cooled to 0 °C. HCl solution (4 N in ethyl acetate, 0.35 mL, 1.4 mmol, 20 eq) was added dropwise at 0 °C, and then the reaction was allowed to warm up to room temperature and stirred for 2 h. The reaction mixture was concentrated under reduced pressure, and the product was partitioned between saturated aqueous NaHCO_3_ and CH_2_Cl_2_. The organic phase was dried over Na_2_SO_4_ and concentrated under reduced pressure to afford pure **Dim22** as a dark brown solid (yield >99%). ^1^H NMR (400 MHz, CDCl_3_): δ 9.26 (br s, 1H), 7.97–7.89 (m, 4H), 7.59–7.55 (m, 3H), 7.47 (s, 1H), 7.37 (s, 1H), 4.17–4.13 (m, 2H), 4.02 (d, *J* = 6.6 Hz, 2H), 3.14–3.07 (m, 2H), 2.74 (s, 3H), 1.92–1.82 (m, 2H), 1.56–1.44 (m, 7H), 0.94–0.85 (m, 4H), 0.60–0.51 (m, 2H). ^13^C NMR (100 MHz, CDCl_3_): δ 137.3 (Cq), 135.8 (Cq), 133.3 (2Cq), 133.0 (Cq), 129.9, 128.9, 128.4, 128.3, 127.8 (2C), 126.9, 126.8, 126.3, 124.7 (Cq), 96.6 (Cq), 51.8, 49.3, 48.0, 38.2, 33.6, 30.1 (2C), 29.7, 29.5, 25.7, 25.3 (2C), 23.5 HRMS (ESI) *m/z*: [M + H]^+^ calcd for C_29_H_37_IN_5_ 582.2094; found 582.2088. Anal. calcd. for C_29_H_36_IN_5_: C, 59.90; H, 6.24; I, 21.82; N, 12.04; found: C, 59.71; H, 6.00; N, 11.91.

##### *tert*-Butyl-(5-(1-(4,4-dimethylpentyl)-5-(naphthalen-2-yl)-2′-((trimethylsilyl)ethynyl)-1*H*,3′*H*-[2,4′-biimidazol]-3′-yl)pentyl)(methyl)carbamate (**9**)

Under nitrogen and in a flame-dried round bottom flask, compound **7** (328 mg, 0.48 mmol, 1 eq), Pd(PPh_3_)_2_Cl_2_ (17 mg, 0.024 mmol, 5% mol), and CuI (9 mg, 0.048 mmol, 10% mol) were dissolved in a dry 3:1 THF/TEA mixture (1.8 mL, 0,3 M) which was previously deoxygenated by bubbling nitrogen for 5 min at −78 °C. Trimethylsilylacetylene (204 μL, 1.44 mmol, 3 eq) was added, and the reaction was stirred at 60 °C for 3 h. The resulting mixture was cooled to room temperature and then partitioned between ethyl acetate/water. The aqueous phase was extracted with ethyl acetate (×3), and the combined organic phases were washed with brine (×2), dried over Na_2_SO_4_, and concentrated under reduced pressure to give a residue that was purified by gravimetric column chromatography (dichloromethane/methanol 99:1). The purified product was obtained as a brown foamy solid (yield 32%). ^1^H NMR (400 MHz, CDCl_3_): δ 7.96–7.88 (m, 4H), 7.58–7.53 (m, 3H), 7.30–7.27 (m, 2H), 4.39 (t, *J* = 7.3 Hz, 2H), 4.10 (t, *J* = 7.3 Hz, 2H), 3.20 (t, *J* = 6.9 Hz, 2H), 2.84 (s, 3H), 1.85–1.76 (m, 2H), 1.59–1.51 (m, 2H), 1.46 (s, 9H), 1.42–1.32 (m, 4H), 0.91–0.85 (m, 2H), 0.68 (s, 9H), 0.31 (s, 9H). ^13^C NMR (100 MHz, CDCl_3_): δ 156.4 (Cq), 139.0 (Cq), 135.1 (Cq), 134.2 (Cq), 134.0 (Cq), 133.6 (Cq), 132.6, 129.2, 129.1, 128.7 (2C), 128.5, 128.2 (Cq), 127.4, 127.3, 127.2, 123.6 (Cq), 101.0 (Cq), 94.3 (Cq), 79.8 (Cq), 49.4, 46.6, 46.5, 41.0, 34.9, 31.0, 30.6 (Cq), 29.7 (3C), 29.1 (3C), 28.1, 26.8, 24.6, 0.3 (3C) HRMS (ESI) *m/z*: [M + H]^+^ calcd for C_39_H_56_N_2_O_2_Si 654.4203; found 654.4207.

##### *tert*-Butyl-(5-(1-(cyclohexylmethyl)-5-(naphthalen-2-yl)-2′-((trimethylsilyl)ethynyl)-1*H*,3′*H*-[2,4′-Biimidazol]-3′-yl)pentyl)(methyl)carbamate (**10**)

Under nitrogen and in a flame-dried round bottom flask, compound **8** (291 mg, 0.43 mmol, 1 eq), Pd(PPh_3_)_2_Cl_2_ (15 mg, 0.021 mmol, 5% mol), and CuI (8 mg, 0.043 mmol, 10% mol) were dissolved in a dry 3:1 THF/TEA mixture (1.5 mL, 0.3 M), which was previously deoxygenated by bubbling nitrogen for 5 min at −78 °C. Trimethylsilylacetylene (181 μL, 1.29 mmol, 3 eq) was added, and the reaction was stirred at 60 °C for 3 h. The resulting mixture was cooled to room temperature and then partitioned between ethyl acetate/water. The aqueous phase was extracted with ethyl acetate (×3), and the combined organic phases were washed with brine (×2), dried over Na_2_SO_4_, and concentrated under reduced pressure to give a residue that was purified by gravimetric column chromatography (dichloromethane/methanol 99:1). The purified product was obtained as a brown foamy solid (yield 44%). ^1^H NMR (400 MHz, CDCl_3_): δ 7.98–7.92 (m, 4H), 7.60–7.55 (m, 3H), 7.37–7.32 (m, 2H), 4.44 (t, *J* = 6.6 Hz, 2H), 4.07 (d, *J* = 6.8 Hz, 2H), 3.21 (t, *J* = 6.6 Hz, 2H), 2.85 (s, 3H), 1.84–1.76 (m, 2H), 1.60–1.54 (m, 2H), 1.47 (s, 9H), 1.39–1.30 (m, 7H), 0.94–0.88 (m, 4H), 0.55–0.53 (m, 2H), 0.33 (s, 9H). ^13^C NMR (100 MHz, CDCl_3_): δ 156.4 (Cq), 139.1 (Cq), 136.0 (Cq), 134.0 (Cq), 133.6 (Cq), 132.6, 129.4, 129.2, 129.1, 128.8 (2C), 128.5, 128.1 (Cq), 127.5, 127.0, 114.7 (Cq), 101.8 (Cq), 93.9 (Cq), 79.9 (Cq), 52.3, 49.1, 46.6, 38.9, 34.9, 31.0, 30.7 (2C), 29.2 (3C), 27.9, 26.5, 26.0 (2C), 24.6, 0.3 (3C) HRMS (ESI) *m/z*: [M + H]^+^ calcd for C_39_H_54_N_5_O_2_Si 652.4047; found 652.4055.

##### 5-(1-(4,4-Dimethylpentyl)-2′-ethynyl-5-(naphthalen-2-yl)-1*H*,3′*H*-[2,4′-biimidazol]-3′-yl)-*N*-methylpentan-1-amine (**Dim3**)

Under nitrogen and in a flame-dried round bottom flask, compound **9** (65 mg, 0.10 mmol) was dissolved in dry ethyl acetate (0.40 mL, 0.2 M) and cooled to 0 °C. HCl solution (4 N in ethyl acetate, 0.40 mL, 1.4 mmol, 20 eq) was added dropwise at 0 °C, and then the reaction was allowed to warm up to room temperature and stirred for 2 h. The reaction mixture was concentrated under reduced pressure, and the product was partitioned between saturated aqueous NaHCO_3_ and CH_2_Cl_2_. The organic phase was dried over Na_2_SO_4_ and concentrated under reduced pressure. The resulting crude product was dissolved in a 1:1 MeOH/THF mixture (5 mL, 0.02 M), potassium carbonate (27 mg, 0.20 mmol, 2 eq) was added, and the reaction was stirred at room temperature for 2 h. The resulting mixture was partitioned between ethyl acetate and water, the aqueous phase was extracted with ethyl acetate (×2), and the combined organic phases were dried over Na_2_SO_4_ and concentrated under reduced pressure. The final product was obtained as a dark brown solid (quantitative overall two-steps yield). HRMS (ESI) *m/z*: [M + H]^+^ calcd for C_31_H_40_N_5_ 482.3284; found 482.3291. Anal. calcd. for C_31_H_39_N_5_: C, 77.30; H, 8.16; N, 14.54; found: C, 77.39; H, 8.27; N, 14.21.

##### 5-(1-(Cyclohexylmethyl)-2′-ethynyl-5-(naphthalen-2-yl)-1*H*,3′*H*-[2,4′-biimidazol]-3′-yl)-*N*-methylpentan-1-mine (**Dim23**)

Under nitrogen, in a flame-dried round bottom flask, compound **10** (62.7 mg, 0.096 mmol) was dissolved in dry ethyl acetate (0.40 mL, 0.2 M) and cooled to 0 °C. HCl solution (4 N in ethyl acetate, 0.40 mL, 1.4 mmol, 20 eq) was added dropwise at 0 °C, and then the reaction was allowed to warm up to room temperature and stirred for 2 h. The reaction mixture was concentrated under reduced pressure, and the product was partitioned between saturated aqueous NaHCO3 and CH_2_Cl_2_. The organic phase was dried over Na_2_SO_4_ and concentrated under reduced pressure. The resulting crude product was dissolved in a 1:1 MeOH/THF mixture (5 mL, 0.02 M), potassium carbonate (26 mg, 0.19 mmol, 2 eq) was added, and the reaction was stirred at room temperature for 2 h. The resulting mixture was partitioned between ethyl acetate and water, the aqueous phase was extracted with ethyl acetate (×2), and the combined organic phases were dried over Na_2_SO_4_ and concentrated under reduced pressure. The final product was obtained as a dark brown solid (quantitative overall two-steps yield). HRMS (ESI) *m/z*: [M + H]^+^ calcd for C_31_H_38_N_5_ 480.3127; found 480.3133. Anal. calcd. for C_31_H_37_N_5_: C, 77.62; H, 7.78; N, 14.60; found: C, 77.82; H, 7.70; N, 14.48.

### Biological Assay

#### Chemicals

Dulbecco’s modified Eagle’s medium (DMEM), stable l-glutamine, fetal bovine serum (FBS), phosphate-buffered saline (PBS), penicillin/streptomycin, chemiluminescent reagent, and 96-well plates were purchased from Euroclone (Milan, Italy). The HMGCoAR assay kit, bovine serum albumin (BSA), Janus Green B, formaldehyde, HCl, and H_2_SO_4_ were from Sigma-Aldrich (St. Louis, MO, USA). The antibody against LDLR and the 3,3′,5,5′-tetramethylbenzidine (TMB) substrate were bought from Thermo Fisher Scientific (Waltham, MA, USA). The Quantikine ELISA kit was bought from R&D Systems (Minnneapolis, MN, USA). The LDL-DyLight 550 was from Cayman Chemical (Ann Arbor, MI, USA). The CircuLex PCSK9 in vitro binding Assay Kit was from CircuLex (CycLex Co., Nagano, Japan). The antibody against HMG-CoAR was bought from Abcam (Cambridge, UK). Phenylmethanesulfonyl fluoride (PMSF), Na-orthovanadate inhibitors, and the antibodies against rabbit Ig-horseradish peroxidase (HRP), mouse Ig-HRP, and SREBP-2 (which recognizes epitope located in a region between 833 and 1141 kDa and bands at about 132 kDa) were purchased from Santa Cruz Biotechnology Inc. (Santa Cruz, CA, USA). The antibodies against hepatocyte nuclear factor 1-alpha (HNF1-alpha) and PCSK9 were bought from GeneTex (Irvine, CA, USA). The inhibitor cocktail Complete Midi was from Roche (Basel, Switzerland). Mini protean TGX pre-cast gel 7.5% and Mini Nitrocellulose Transfer Packs were purchased from BioRad (Hercules, CA, USA).

#### In Vitro PCSK9-LDLR Binding Assay

Diimidazole analogues (0.1–1 × 10^6^ nM) were tested using the in vitro PCSK9-LDLR binding assay (CycLex Co., Nagano, Japan) following the manufacturer’s instructions and with the conditions already optimized.^15^ Briefly, the plates were pre-coated with a recombinant LDLR-AB domain containing the binding site of PCSK9. Before starting the assay, the tested analogues and/or the vehicle in DMSO were sonicated at 37 °C, diluted in the reaction buffer, and added in microcentrifuge tubes. Afterward, the reaction mixtures were added in each well of the microplate, and the reaction was started by adding His-tagged PCSK9 solution (3 μL). The microplate was allowed to incubate for 2 h at room-temperature (RT) shaking at 300 rpm on an orbital microplate shaker. Subsequently, the wells were washed 4 times with the wash buffer. After the last wash, the biotinylated anti-His-tag monoclonal antibody (100 μL) was added and incubated at RT for 1 h shaking at 300 rpm. After incubation, the wells were washed 4 times with wash buffer. After the last wash, 100 μL of HRP-conjugated streptavidin were added, and the plate was incubated for 20 min at RT. After incubation, the wells were washed 4 times with wash buffer. Finally, the substrate reagent (tetra-methylbenzidine) was added, and the plate was incubated for 10 min at RT shaking at ca. 300 rpm. The reaction was stopped with 2.0 M sulfuric acid, and the absorbance at 450 nm was measured using a Synergy H1 fluorescent plate reader (Winooski, VT, USA).

#### HMG-CoAR Activity Assay

The experiments were carried out following the manufacturer’s instructions and optimized protocol.^43^ The assay buffer, NADPH, substrate solution, and HMG-CoAR were provided in the HMG-CoAR Assay Kit (Sigma Aldrich SRL, Milan, Italy). The experiments were carried out following the manufacturer’s instructions at 37 °C. In particular, each reaction (200 μL) was prepared adding the reagents in the following order: 1× assay buffer, a 10–500 μM dose of sonicated Dim analogues or vehicle (C), NADPH (4 μL), the substrate solution (12 μL), and finally, HMG-CoAR (catalytic domain) (2 μL). Subsequently, the samples were mixed, and the absorbance at 340 nm was read by a microplate reader (Synergy H1, Winooski, VT, USA) at times of 0 and 10 min. The HMGCoAR-dependent oxidation of NADPH and the inhibition properties of the peptides were measured by absorbance reduction, which is directly proportional to enzyme activity.

#### HepG2 Cell Culture Conditions and Treatment

The HepG2 cell line was bought from ATCC (HB-8065, ATCC from LGC Standards, Milan, Italy) and cultured in DMEM high glucose with stable l-glutamine supplemented with 10% FBS, 100 U/mL penicillin, and 100 μg/mL streptomycin (complete growth medium) with incubation at 37 °C under a 5% CO_2_ atmosphere.

#### In-Cell Western Assay

For the experiments, a total of 3 × 10^4^ HepG2 cells/well were seeded in 96-well plates. The following day, the cells were washed with PBS and then starved overnight (O/N) in DMEM without FBS and antibiotics. After starvation, the HepG2 cells were treated with 4.0 μg/mL PCSK9-WT and 4.0 μg/mL PCSK9 + diimidazole analogues and vehicle (H_2_O) for 2 h at 37 °C under a 5% CO_2_ atmosphere. Subsequently, they were fixed in 4% paraformaldehyde for 20 min at room temperature (RT). Cells were washed 5 times with 100 μL of PBS/well (each wash was for 5 min at RT), and the endogenous peroxide activity was quenched by adding 3% H_2_O_2_ for 20 min at RT. Non-specific sites were blocked with 100 μL/well of 5% bovine serum albumin (BSA, Sigma) in PBS for 1.5 h at RT. LDLR primary antibody solution (1:3000 in 5% BSA in PBS, 25 μL/well) was incubated O/N at +4 °C. Subsequently, the primary antibody solution was discarded, and each sample was washed 5 times with 100 μL/well of PBS (each wash was for 5 min at RT). Goat anti-rabbit Ig-HRP secondary antibody solution (Santa Cruz) (1:6000 in 5% BSA in PBS, 50 μL/well) was added and incubated 1 h at RT. The secondary antibody solution was washed 5 times with 100 μL/well of PBS (each wash for 5 min at RT). Freshly prepared TMB substrate (Pierce, 100 μL/well) was added, and the plate was incubated at RT until the desired color was developed. The reaction was stopped with 2 M H_2_SO_4_, and then the absorbance at 450 nm was measured using a microplate reader (Synergy H1, Winooski, VT, USA). After the reading, the cells were stained by adding 1× Janus Green stain, incubating for 5 min at RT. The dye was removed, and the sample was washed 5 times with water. Afterward, 100 μL of 0.5 M HCl for well was added, and the solution was incubated for 10 min. After 10 s of shaking, the OD at 595 nm was measured using the microplate reader (Synergy H1, Winooski, VT, USA).

#### Fluorescent LDL Uptake

HepG2 cells (3 × 10^4^/well) were seeded in 96-well plates and kept in complete growth medium for 2 days before treatment. On the third day, the cells were washed with PBS and then starved overnight (O/N) in DMEM without FBS and antibiotics. After starvation, they were treated with 4.0 μg/mL PCSK9, 4.0 μg/mL PCSK9 + diimidazole analogues, and vehicle (H_2_O) for 2 h with at 37 °C under a 5% CO_2_ atmosphere. At the end of the treatment, the culture medium was replaced with 50 μL/well LDL-DyLight 550 working solution (Cayman Chemical Company, Ann Arbor, MI, USA) prepared in DMEM without FBS and antibiotics. The cells were additionally incubated for 2 h at 37 °C, and then the culture medium was aspirated and replaced with PBS (100 μL/well). The degree of LDL uptake was measured using a Synergy H1 fluorescent plate reader (Winooski, VT, USA) (excitation and emission wavelengths of 540 and 570 nm, respectively). Fluorescent LDL uptake was finally assessed following optimized protocol.^14^

### Statistical Analysis

All the data sets were checked for normal distribution by D’Agostino and Pearson tests. Since they are all normally disturbed with *p* values of <0.05, we proceeded with statistical analyses of one-way ANOVA followed by Tukey’s post hoc test using Graphpad Prism 9 (San Diego, CA, USA). Values were reported as means ± s.d.; *p* values of <0.05 were considered to be significant. The IC_50_ values of samples inhibiting PCSK9-LDLR binding were evaluated by using the log(inhibitor) vs response model of GraphPad Prism 9 (San Diego, CA, USA). This model assumes that the dose–response curves has a standard slope, equal to a Hill slope (or slope factor) of −1.0. This is the slope expected when a ligand binds to a receptor following the law of mass action and is the slope expected of a dose–response curve when the second messenger created by receptor stimulation binds to its receptor by the law of mass action (https://graphpad.com/guides/prism/latest/curvefitting/reg_dr_inhibit.htm).

### Blood Collection

Blood was collected by venipuncture of the antecubital vein of healthy volunteers (*n* = 5, 2 males and 3 females, mean age 37 ± 15 years) who did not take antiplatelet drugs within 10 days before blood donation and gave their informed consent to participate in the study. Whole blood (WB) was drawn with a 19-gauge needle without venous stasis into citrate (1/10 volume of 0.129 M sodium citrate), discarding the first 4 mL. Platelet-rich plasma (PRP) was obtained by centrifuging whole blood for 10 min at 100*g* without breaks at room temperature as previously described.^44^

### Metabolic Stability in Liver Microsomes

Test compound (**Dim22**) in duplicate was dissolved in DMSO to obtain 1 mM solutions and pre-incubated at the final concentration of 5 μM for 10 min at 37 °C in potassium phosphate buffer 50 mM (pH 7.4), 3 mM MgCl_2_, and mouse and human liver microsomes (Sigma Aldrich) at a final concentration of 0.5 mg/mL. After the pre-incubation period, the reaction was started by adding the cofactor mixture (NADP, Glc6P, and Glc6P-DH in 2% sodium bicarbonate) and UDPGA. Samples (25 μL) were taken at times of 0, 10, 20, 30, 45, and 60 min and then mixed with 150 μL of ACN to stop the reaction. After centrifugation, the supernatants were analyzed by LC–MS/MS. A control sample without cofactors was included in the assay in order to check the stability of the test compounds in the matrix after 60 min. 7-Ethoxycoumarin (7-EC) and 7-hydroxycoumarin (7-OHC) were contemporarily tested as reference standards for the Phase I and Phase II reactions, respectively.

### Solubility in pH 7.4

Solubility was tested after incubation of the test item at 200 μM and 500 μM in PBS buffer pH 7.4 starting from an initial DMSO solution of **Dim22** 100 mM. Samples in duplicates were incubated at 37 °C for 90 min, were then centrifuged at 12500 rpm for 5 min. Supernatants were analyzed in LC/MS together with a solution of the compound in ACN.

### Platelet Function Assessment

Platelet function was assessed by light transmission aggregometry using PAP-8 aggregometer (BioData). Lag time and area under the curve (AUC) were used as the main parameters describing the kinetic of platelet aggregation as previously described.^3^ Briefly, PRP was preincubated with PCSK9 (5 μg/mL, 2 min at 37 °C) followed by epinephrine (0.16 μM) stimulation. To test the effect of **Dim16** on PCSK9-dependent platelet aggregation, PCSK9 was preincubated with **Dim16** (10 nM) for 15 min at room temperature and then added to PRP.

## Supporting Information Available

Supporting figures and tables; copies of ^1^H and ^13^C NMR spectra of all new compounds (PDF)
Molecular formula strings (CSV)

## Abbreviations Used

BSA: bovine serum albumin
CV: cardiovascular
DMEM: Dulbecco’s modified Eagle’s medium
FBS: fetal bovine serum
HepG2: human hepatoma G2
HMG-CoAR: 3-hydroxy-3-methylglutaryl co-enzyme A reductase
HNF-1 α: hepatocyte nuclear factor 1 alpha
HRP: horseradish peroxidase
ICW: in-cell Western assay
LDL: low-density lipoprotein
LDL-C: LDL cholesterol
LDLR: LDL receptor
MD: molecular dynamics
MM-GBSA: molecular mechanics-generalized Born surface area
PBS: phosphate-buffered saline
PCSK9: proprotein convertase subtilisin/kexin 9
PMSF: phenylmethanesulfonyl fluoride
PPIs: protein–protein interactions
RMSD: root mean square deviation
RT: room temperature
SDS: sodium dodecyl sulfate
SREBP: sterol regulatory element binding proteins
