# Supplementary material for: Computational Design, Synthesis, and Biological Evaluation of Diimidazole Analogues Endowed with Dual PCSK9/HMG-CoAR-Inhibiting Activity
Source: J Med Chem. 2023 Jun 1;66(12):7943–58. doi: 10.1021/acs.jmedchem.3c00279 (PMC10291552; doi:10.1021/acs.jmedchem.3c00279)
Supplement: Supplementary file 1 — jm3c00279_si_001.pdf [file jm3c00279_si_001.pdf]

Electronic Supporting Informations *for*

Computational Design, Synthesis, and Biological Evaluation of Diimidazole Analogs Endowed with Dual PCSK9/HMG-CoAR Inhibiting Activity.

Carmen Lammi[1]*, Enrico M. A. Fassi[1], Marco Manenti[2], Marta Brambilla[3], Maria Conti[3], Jianqiang Li[1], Gabriella Roda[1], Marina Camera[1,3], Alessandra Silvani[2,]*, Giovanni Grazioso[1,]*

[1]Dipartimento di Scienze Farmaceutiche, Università degli Studi di Milano, Via L. Mangiagalli 25, 20133 Milan, Italy

[2]Dipartimento di Chimica, Università degli Studi di Milano, Via Golgi 10, 20133 Milan, Italy

[3] Centro Cardiologico Monzino IRCCS, via Parea 4, 20138 Milan, Italy

Corresponding author information. Carmen Lammi (carmen.lammi@unimi.it), Alessandra Silvani (alessandra.silvani@unimi.it), Giovanni Grazioso (giovanni.grazioso@unimi.it).

Table of contents:

- Figures S1, S2, S3, and S4.
- Tables S1, S2, and S3, and S4.
- Copies of ^{1}H and ^{13}C NMR spectra of all new compounds.

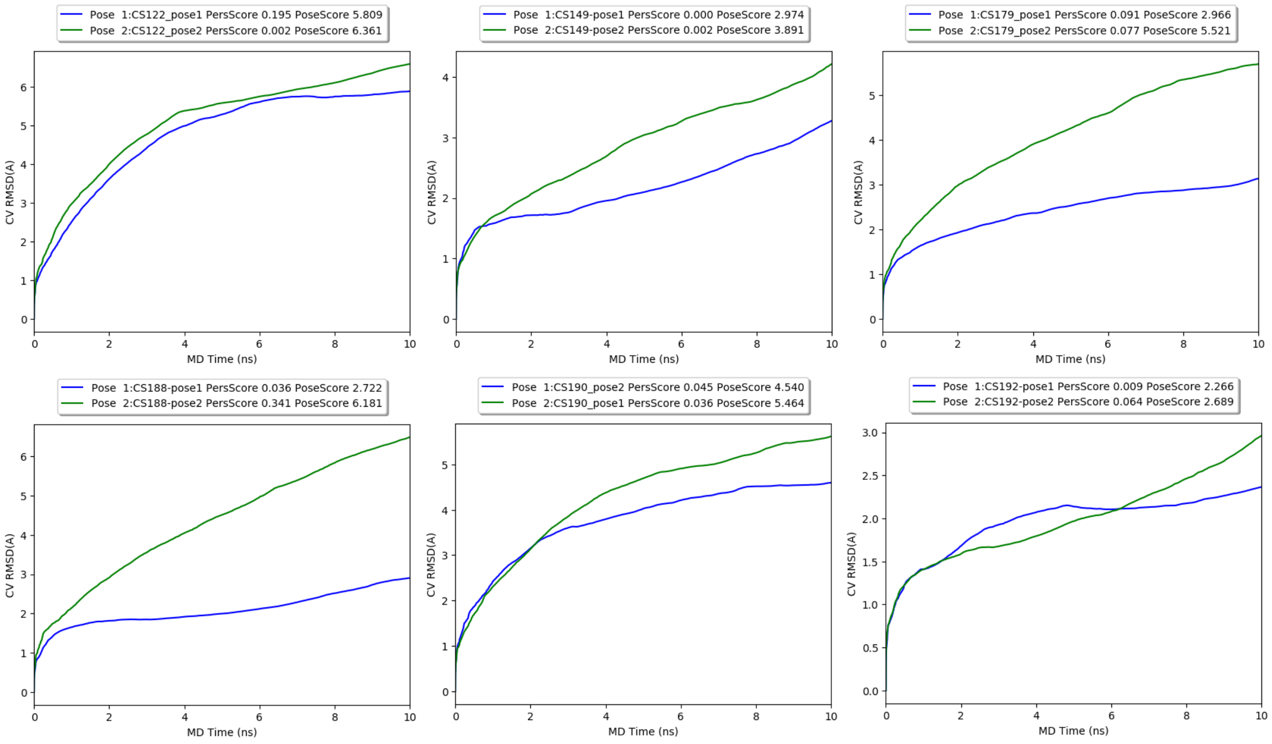

Figure S1. Binding Pose Metadynamics (BPMD) simulations of the two best docking poses of the 6 molecules synthesized and assayed (Table 3 of the main text).

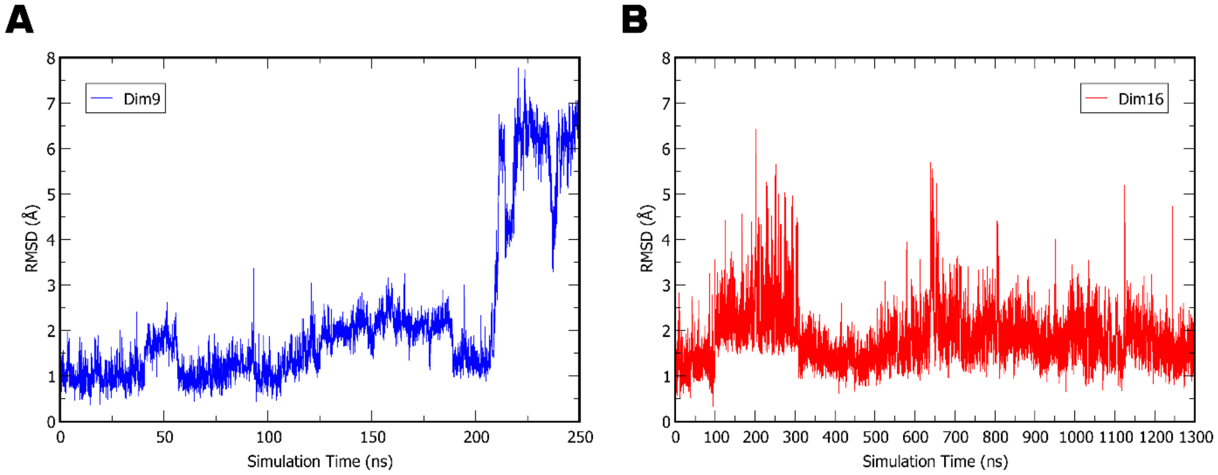

Figure S2. RMSD vs simulation time plot of **Dim9** (A) and **Dim16** (B) in complex with PCSK9 protein.

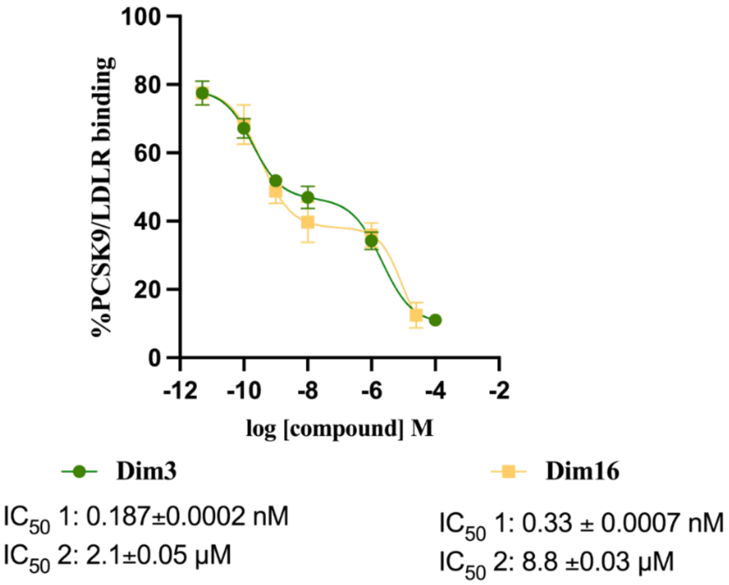

Figure S3. Inhibition of the protein–protein interaction between PCSK9 and LDLR, considering a biphasic behaviour.

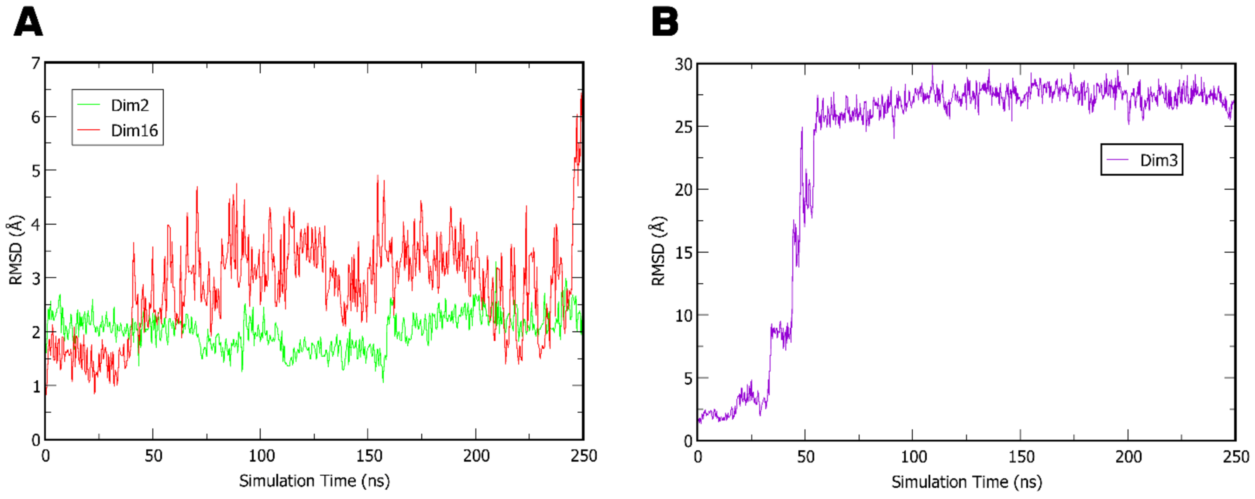

Figure S4. RMSD vs simulation time plots of (A) **Dim2** (green), **Dim16** (red), and (B) **Dim3** (purple) in complex with HMG-CoAR protein. In the case of **Dim2** and **Dim16**, the range 50-250 ns was considered for the DG* calculation.

Table S1. **Dim22** kinetic solubility profile at pH 7.4.

	1 rep µM	2 repl µM	Mean µM	±SD
Sol 200uM	5.92	8.32	7.12	1.70
Sol 500uM	10.38	14.02	12.20	2.57

Table S2. Percentage of **Dim22** remaining in mouse liver microsomes.

	Time (min)	1 rep	2 rep	Mean	SD
**Dim22**	0	100.0	100.0	100.0	0.0
	10	99.7	78.0	88.8	15.3
	20	77.8	74.8	76.3	2.2
	30	49.2	38.7	43.9	7.4
	45	23.3	16.3	19.8	4.9
	60	20.2	11.9	16.0	5.9
**7-EC**	0	100.0	100.0	100.0	0.0
	10	33.6	16.7	25.2	12.0
	30	6.1	3.0	4.5	2.2
	45	0.4	0.4	0.4	0.0
	60	0.1	0.1	0.1	0.0
**7-OHC**	0	100.0	100.0	100.0	0.0
	10	11.4	10.0	10.7	1.0
	30	5.4	6.3	5.9	0.6
	45	0.5	-	0.5	
	60	-	-	-	

Table S3. Percentage of **Dim22** remaining in human liver microsomes.

	Time (min)	1 rep	2 rep	Mean	SD
**Dim22**	0	100.0	100.0	100.0	0.0
	10	93.8	97.6	95.7	2.7
	20	86.5	82.5	84.5	2.8
	30	39.5	48.7	44.1	6.5
	45	35.6	35.0	35.3	0.4
	60	17.8	16.5	17.1	1.0
**7EC**	0	100.0	100.0	100.0	0.0
	10	66.3	64.8	65.6	1.0
	30	40.3	40.8	40.5	0.4
	45	41.5	38.7	40.1	2.0
	60	35.9	37.9	36.9	1.4
**7-OHC**	0	100.0	100.0	100.0	0.0
	10	18.6	13.1	15.8	3.9
	20	6.4	4.5	5.4	1.3
	30	3.9	2.5	3.2	1.0
	60	2.0	1.5	1.7	0.3

Table S4. *In vitro* clearance classification.

	**Cli (μL/min/mg)***		
**Classification**	**Stable**	**Medium**	**High**
**Mouse**	≤2.5	2.5-66	>66
**Human**	≤1.8	1.8-48	>48

* data were extrapolated from references [1-3].

^{1}H NMR (400 MHz, $CDCl_3$) of compound **1**

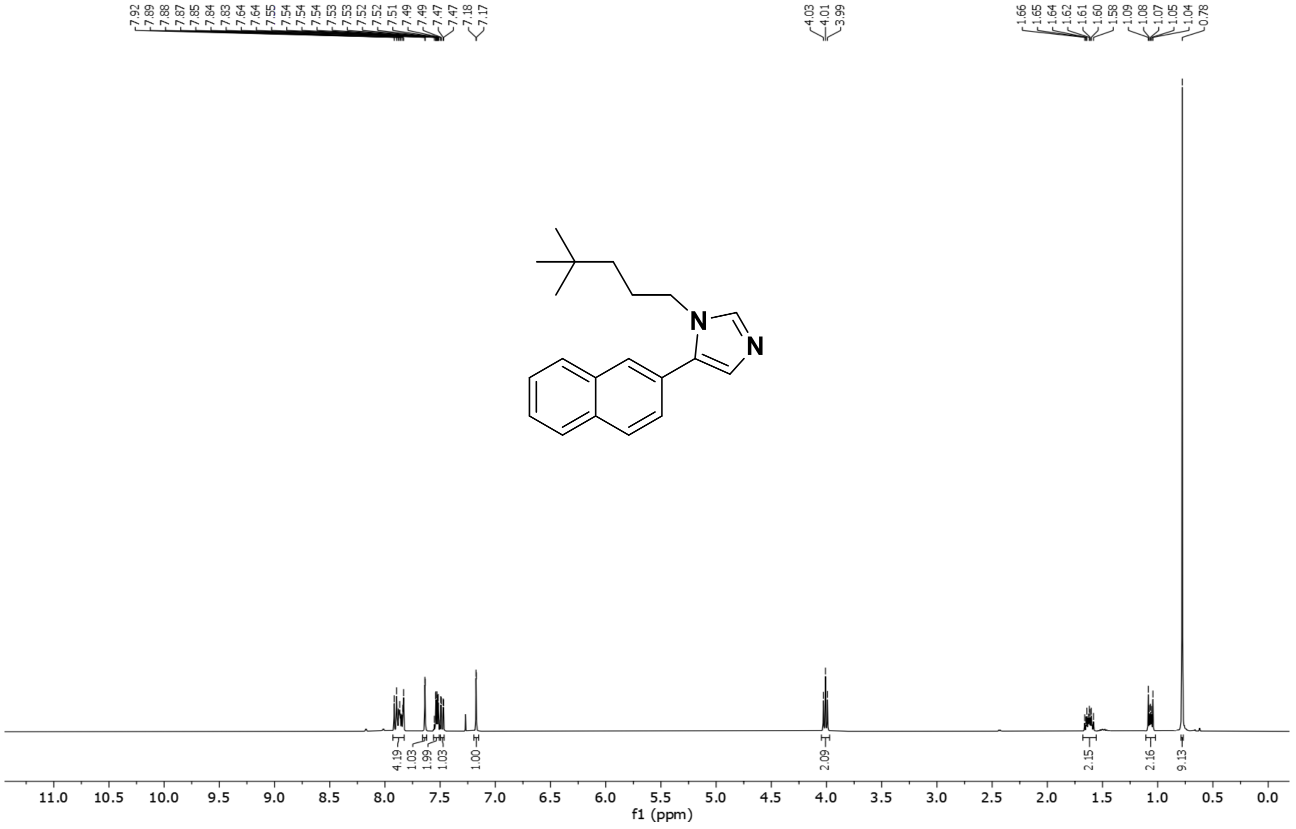

^{13}C NMR (100 MHz, $CDCl_3$) of compound **1**

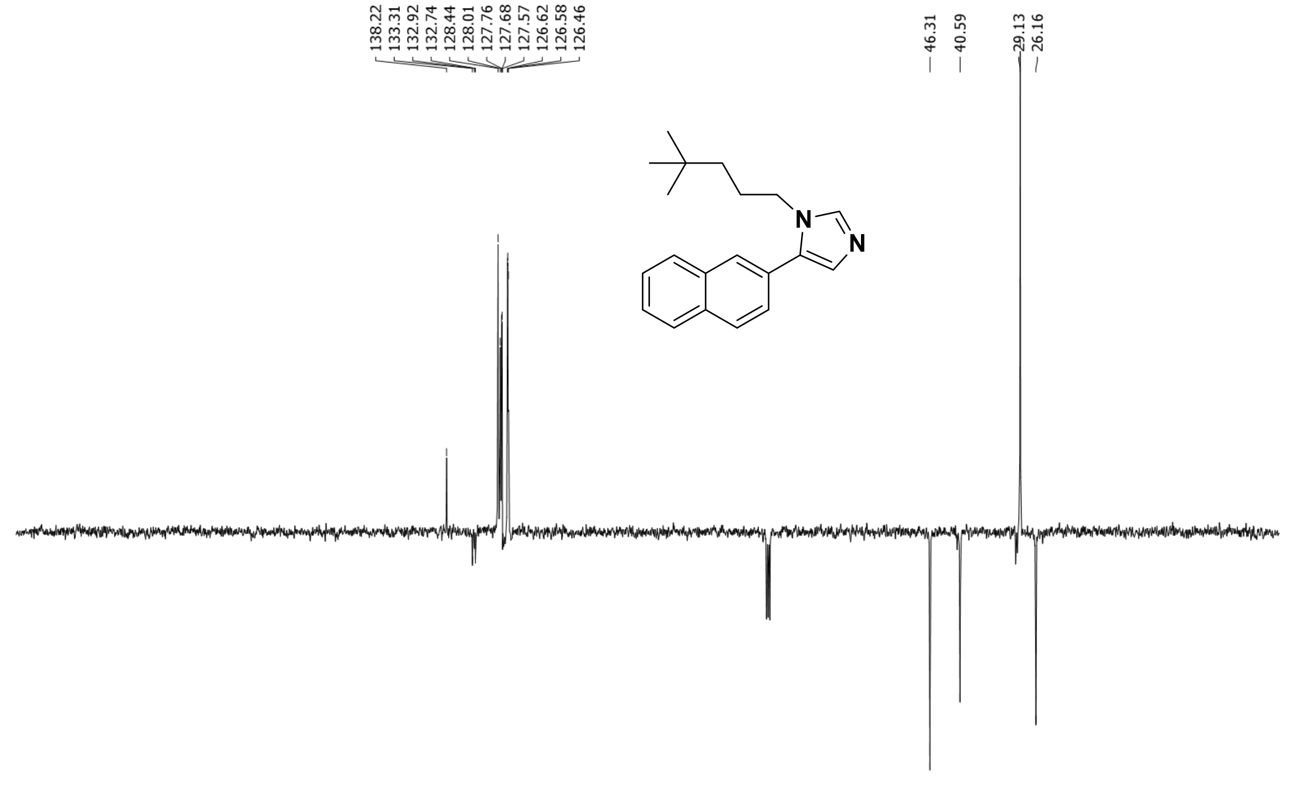

^{1}H NMR (400 MHz, $CDCl_3$) of compound **2**

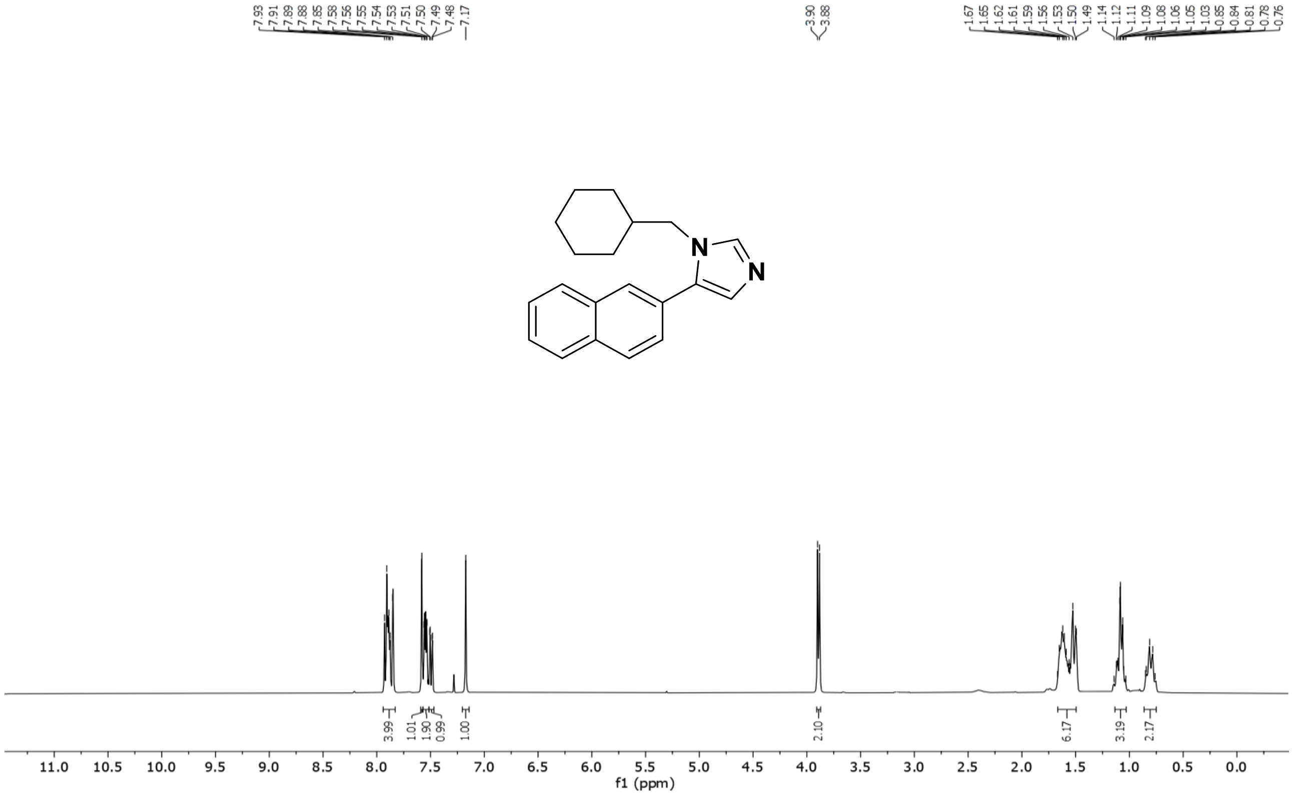

^{13}C NMR (100 MHz, $CDCl_3$) of compound **2**

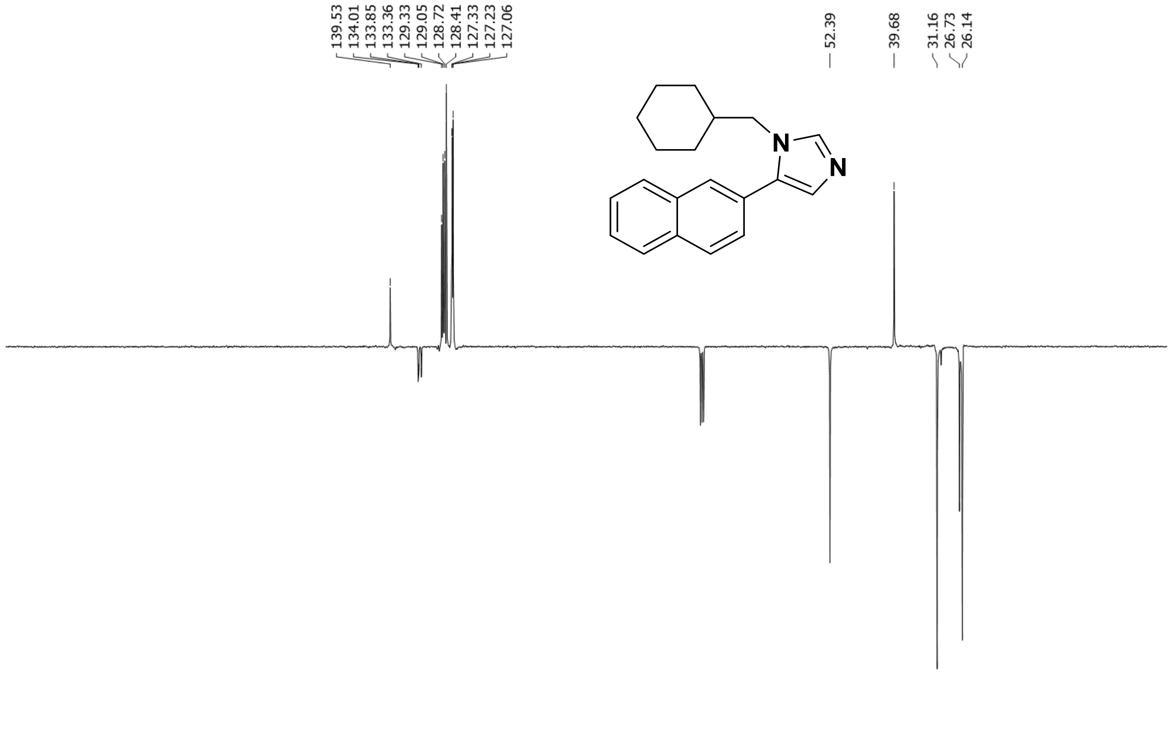

^{1}H NMR (400 MHz, $CDCl_3$) of compound **3**

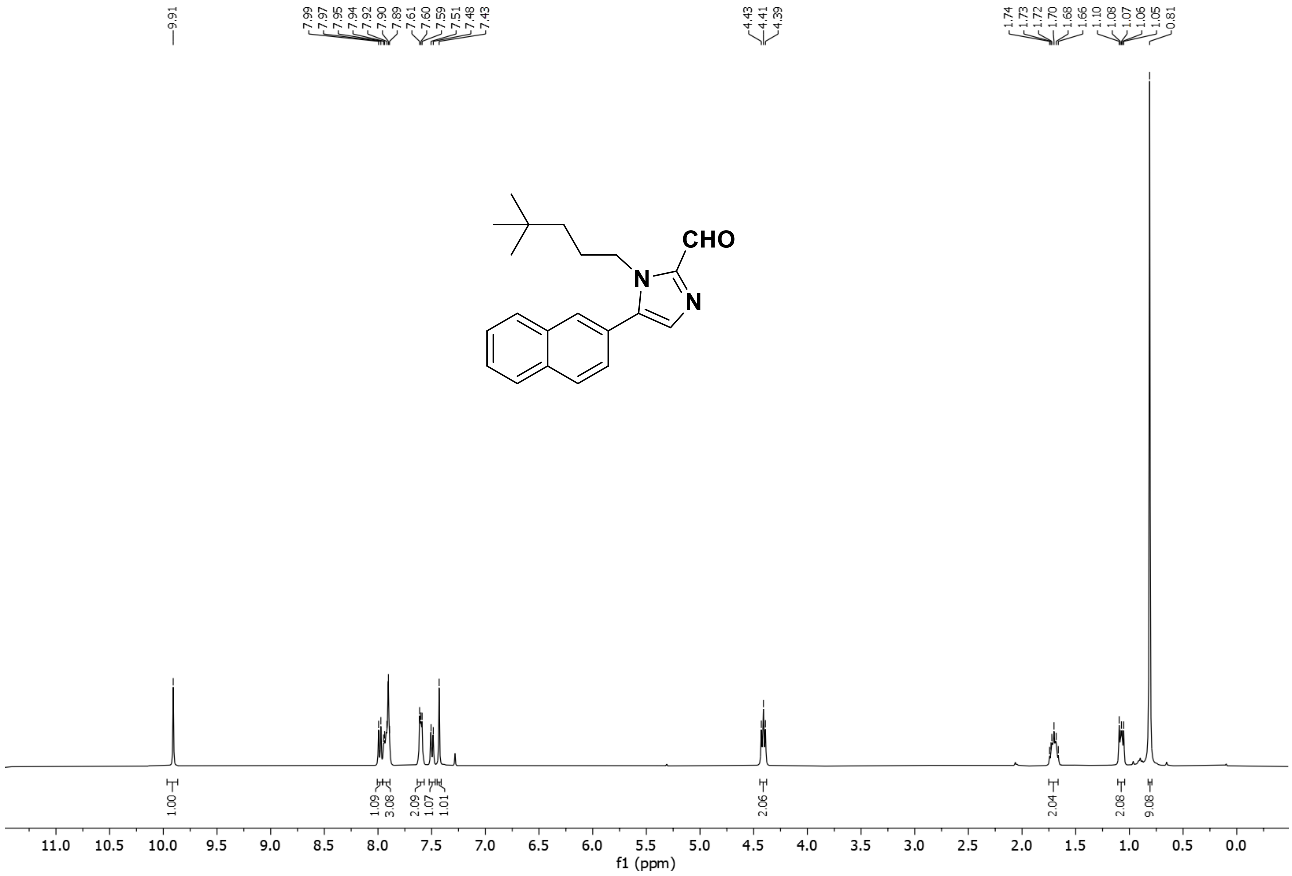

^{13}C NMR (100 MHz, $CDCl_3$) of compound **3**

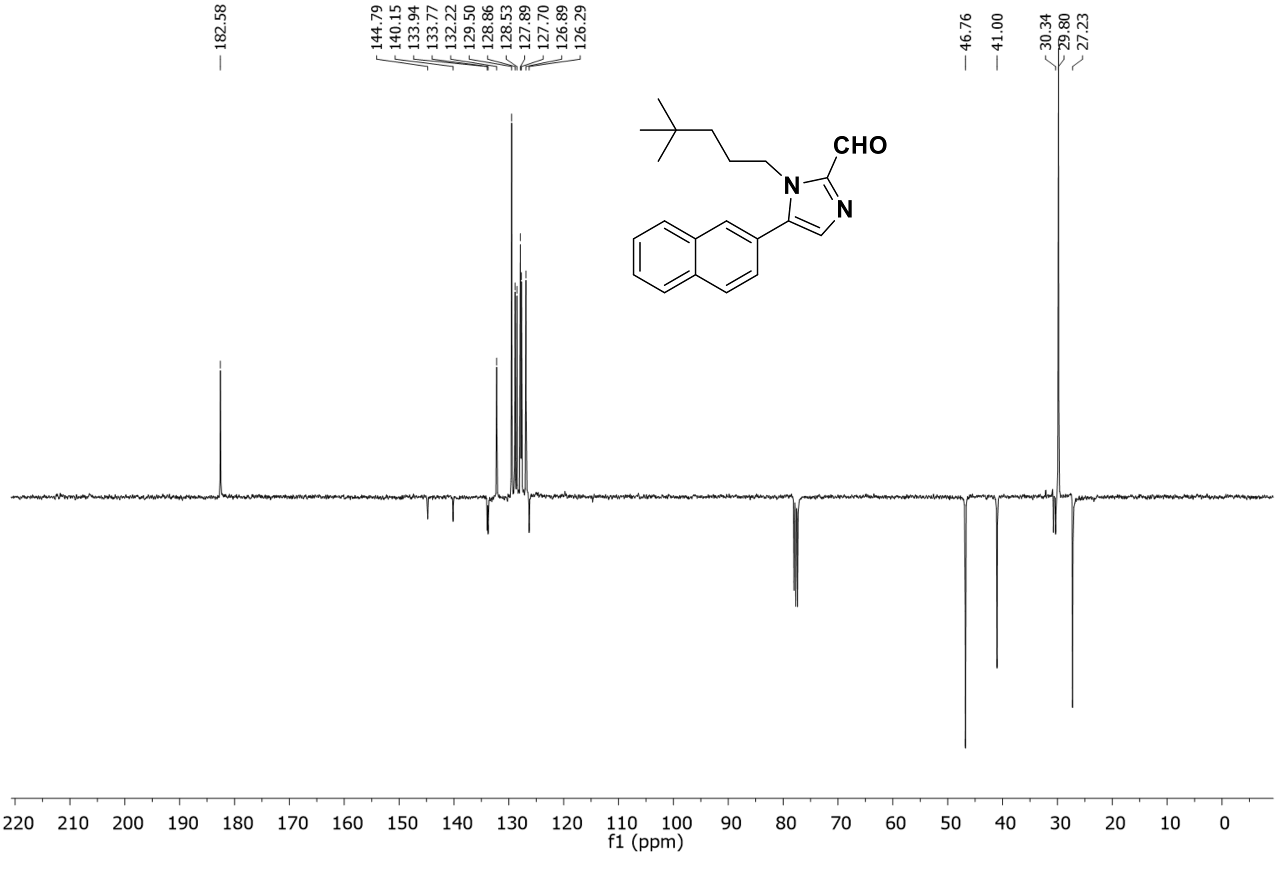

^{1}H NMR (400 MHz, $CDCl_3$) of compound **4**

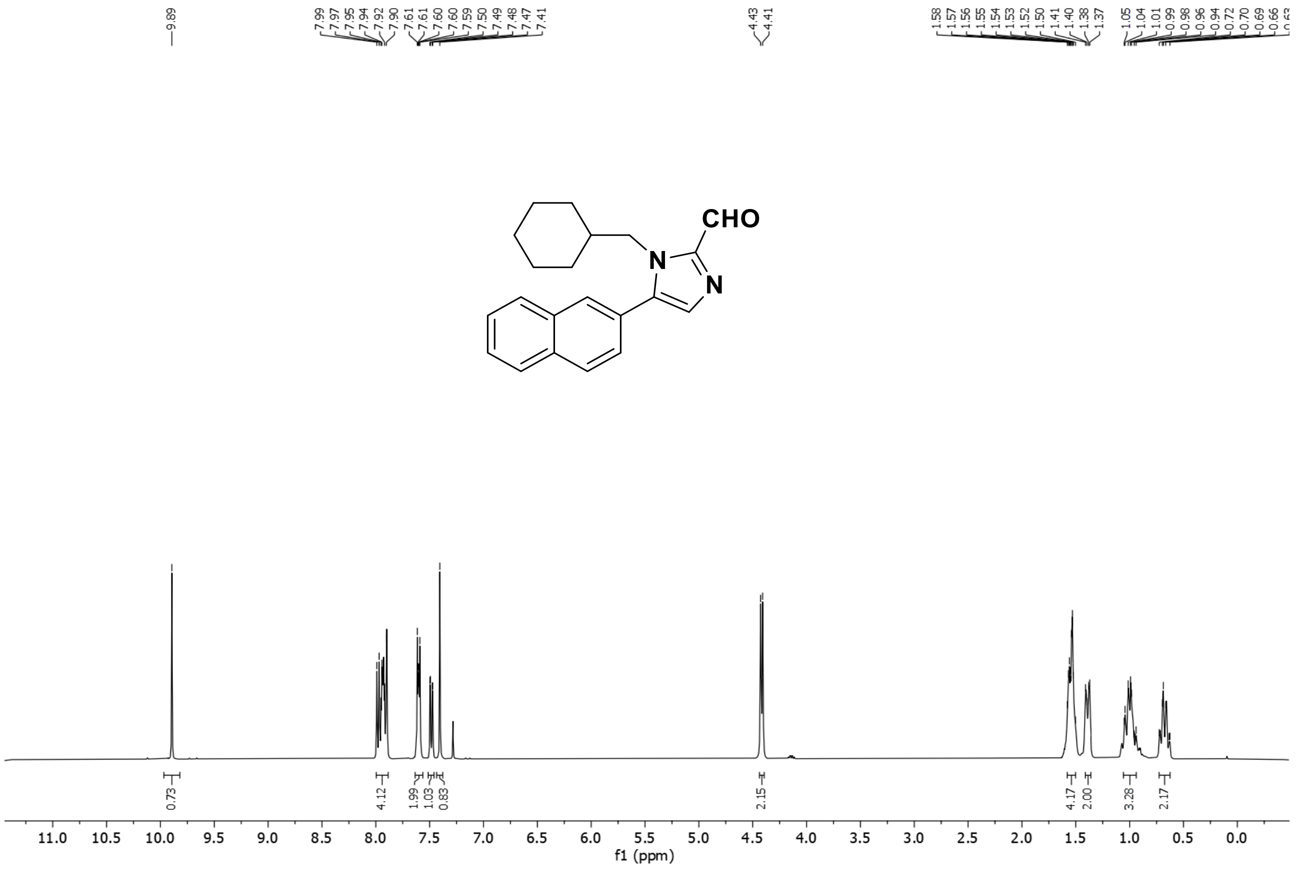

^{13}C NMR (100 MHz, $CDCl_3$) of compound **4**

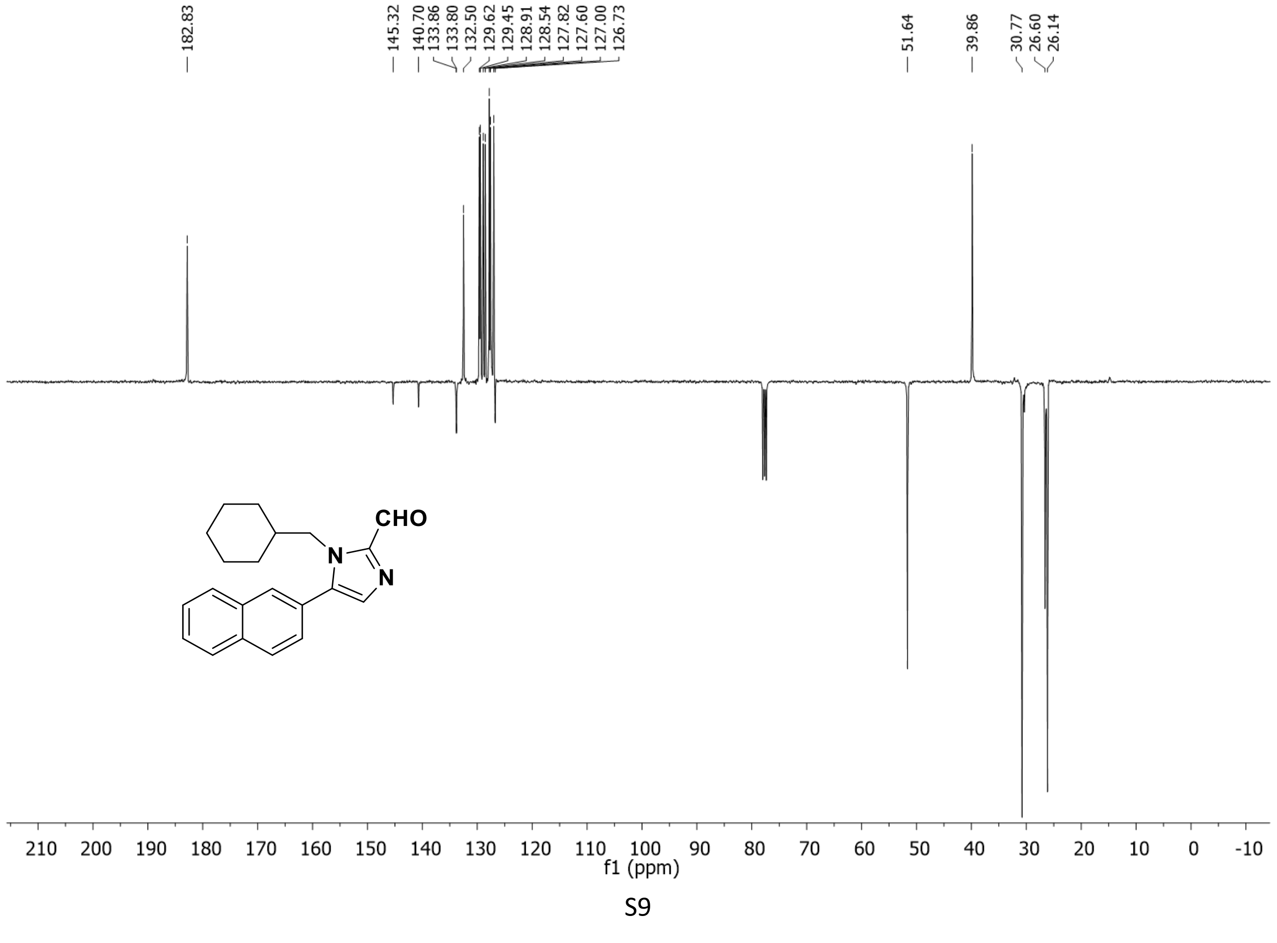

^{1}H NMR (400 MHz, $CDCl_3$) of compound **5**

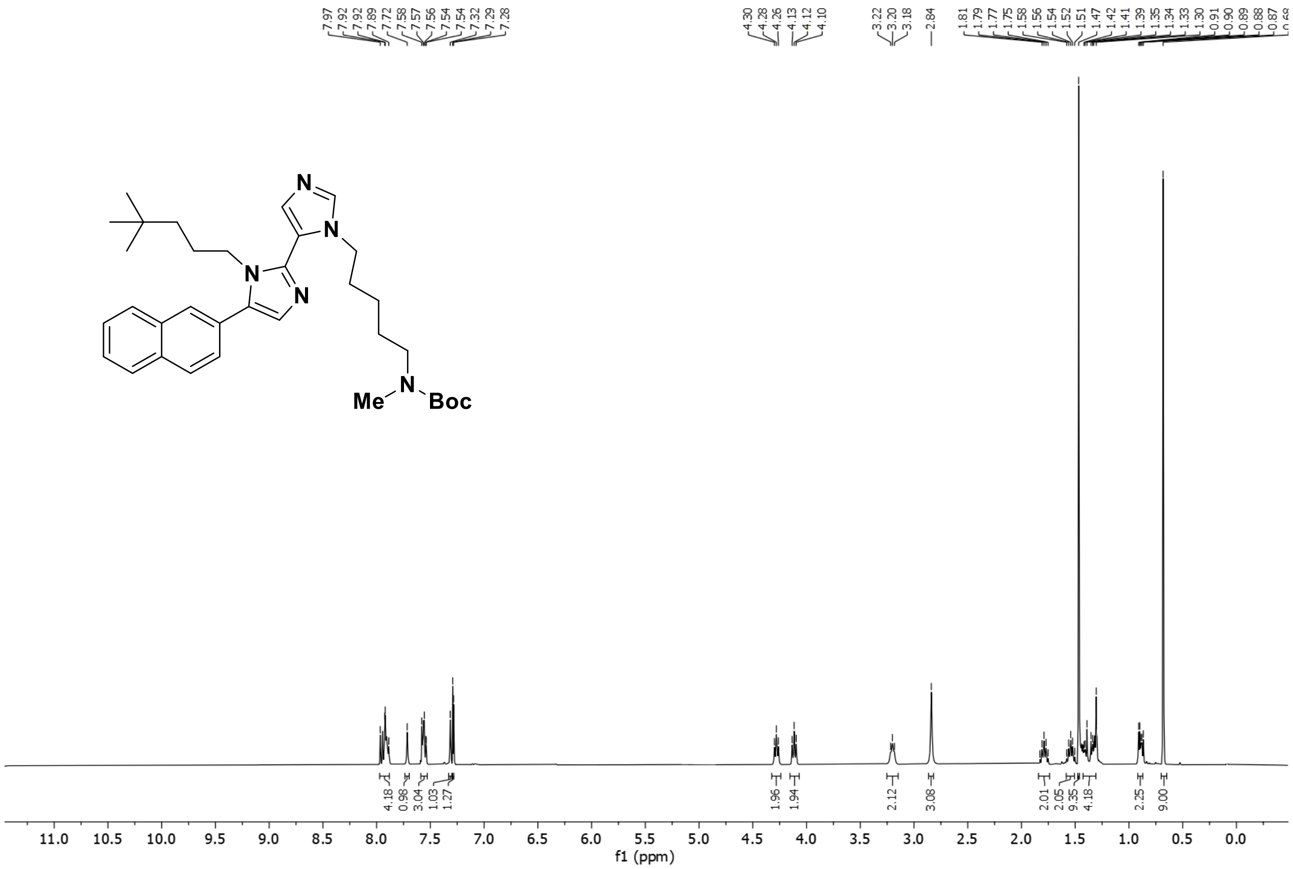

^{13}C NMR (100 MHz, $CDCl_3$) of compound **5**

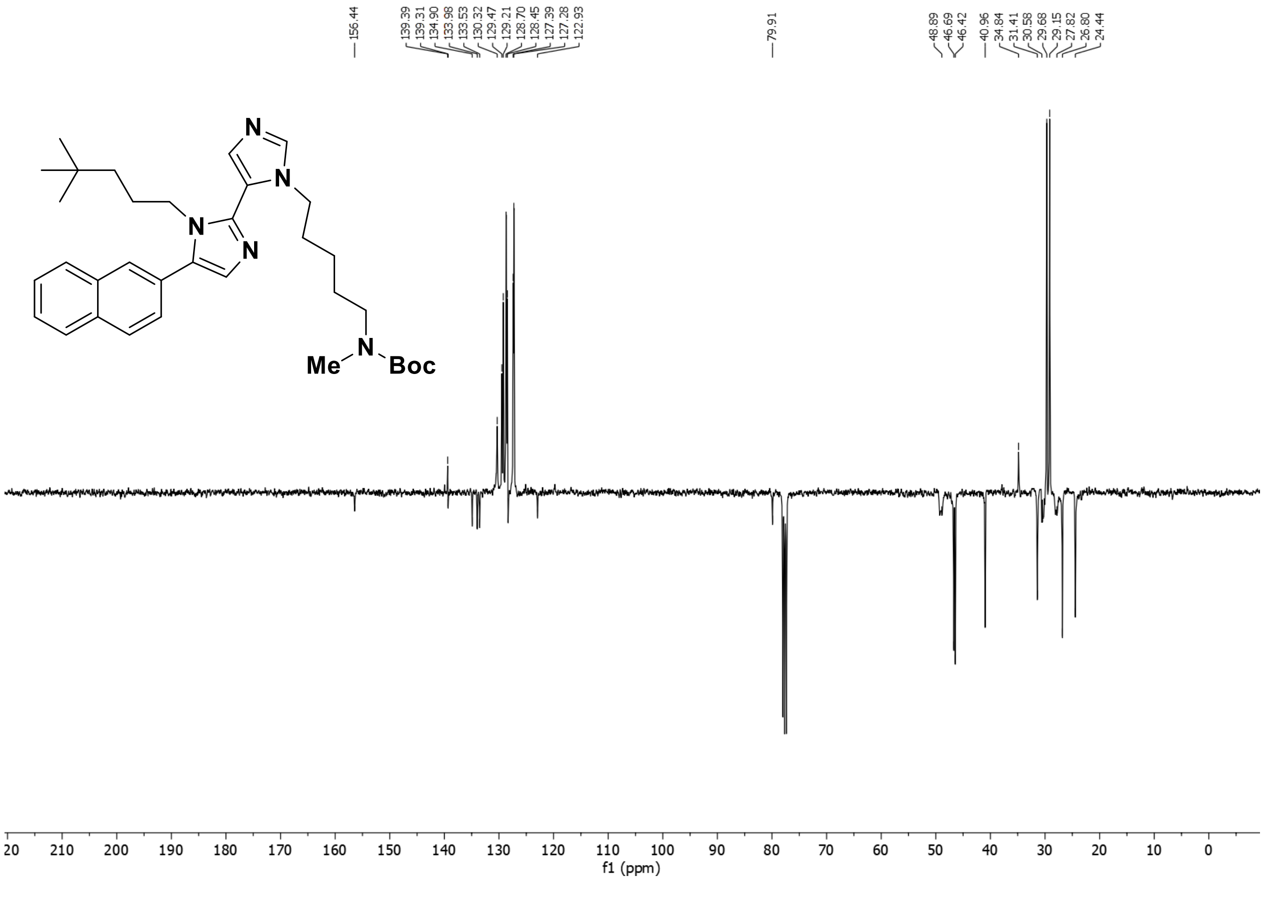

^{1}H NMR (300 MHz, $CDCl_3$) of compound **6**

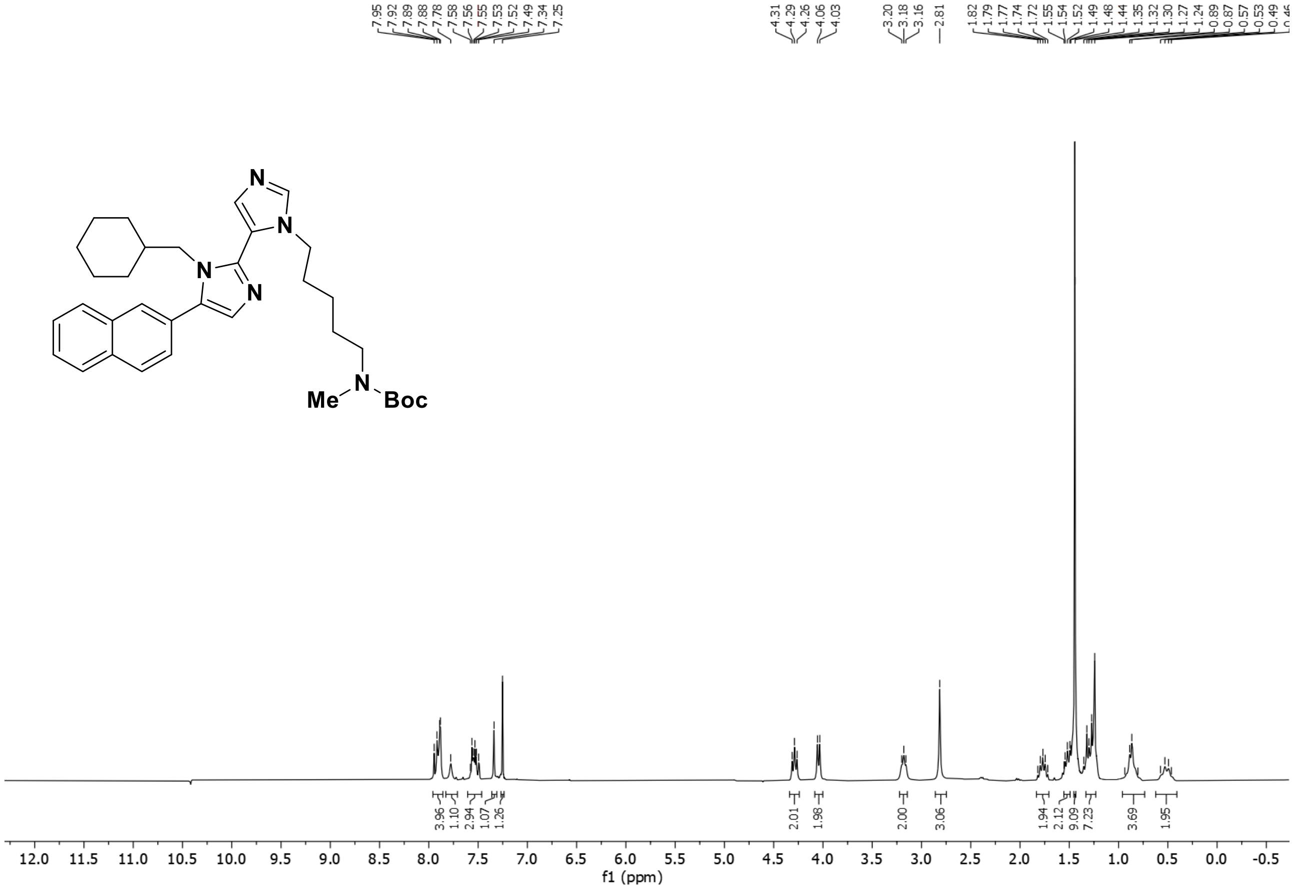

^{13}C NMR (100 MHz, $CDCl_3$) of compound **6**

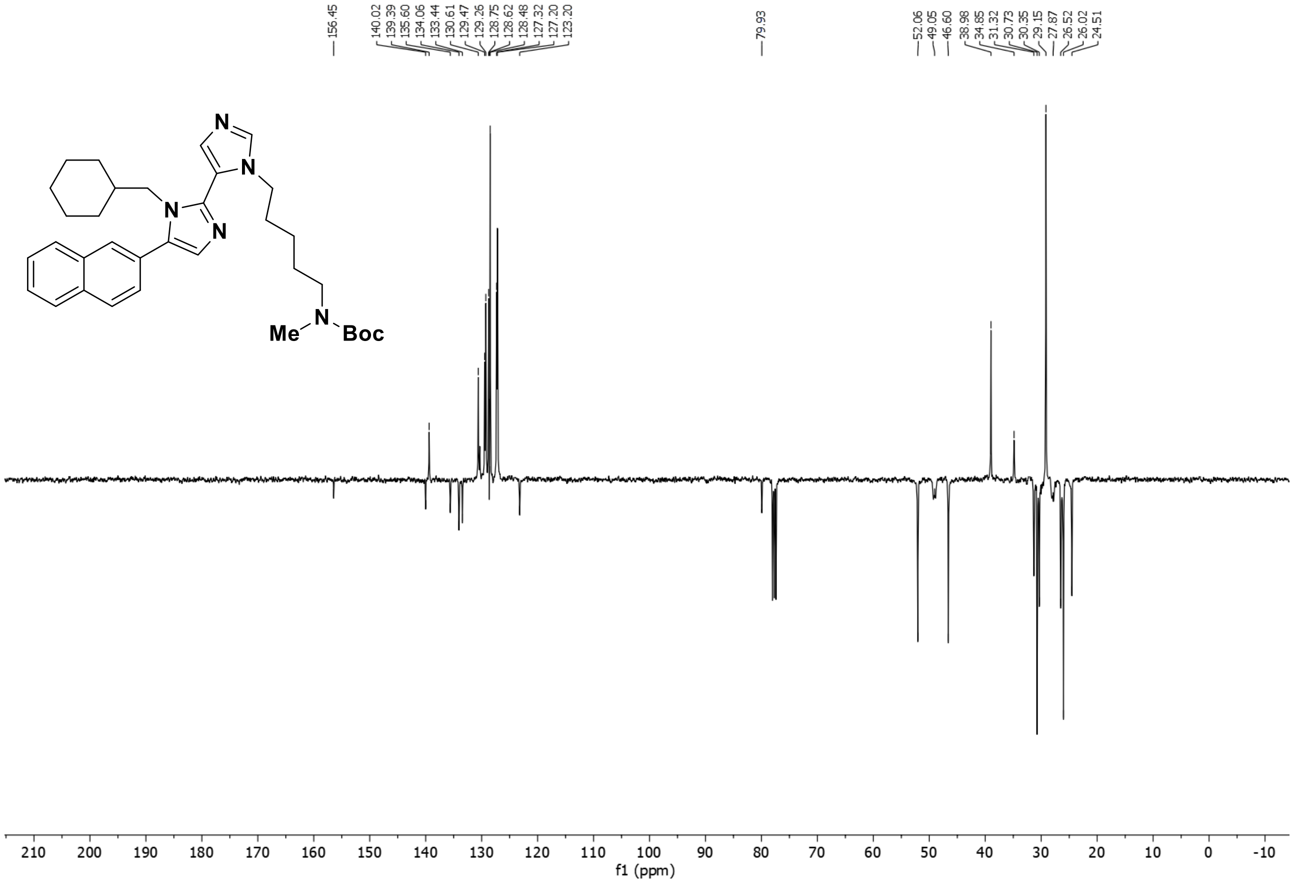

^{1}H NMR (400 MHz, $CDCl_3$) of compound **Dim2**

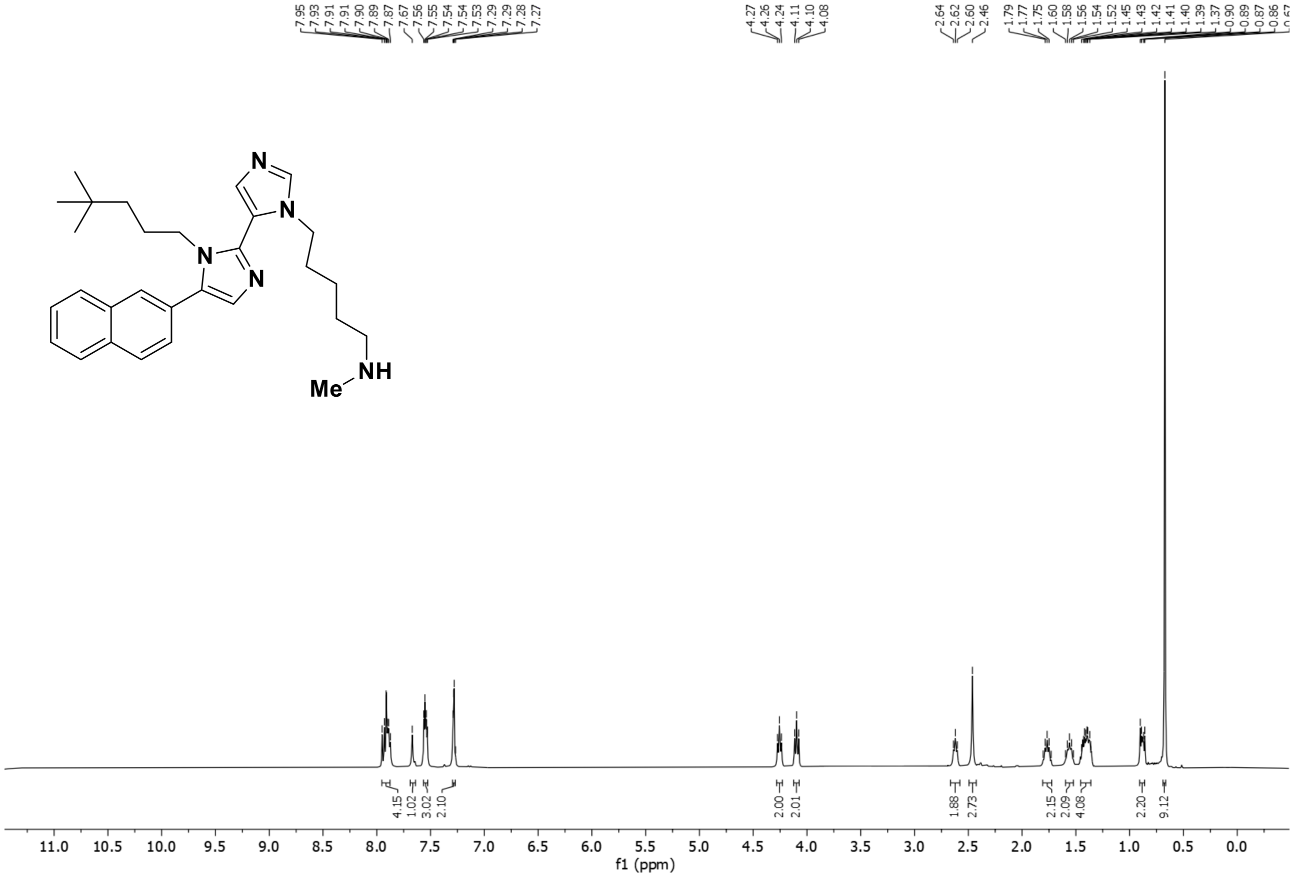

^{13}C NMR (100 MHz, $CDCl_3$) of compound **Dim2**

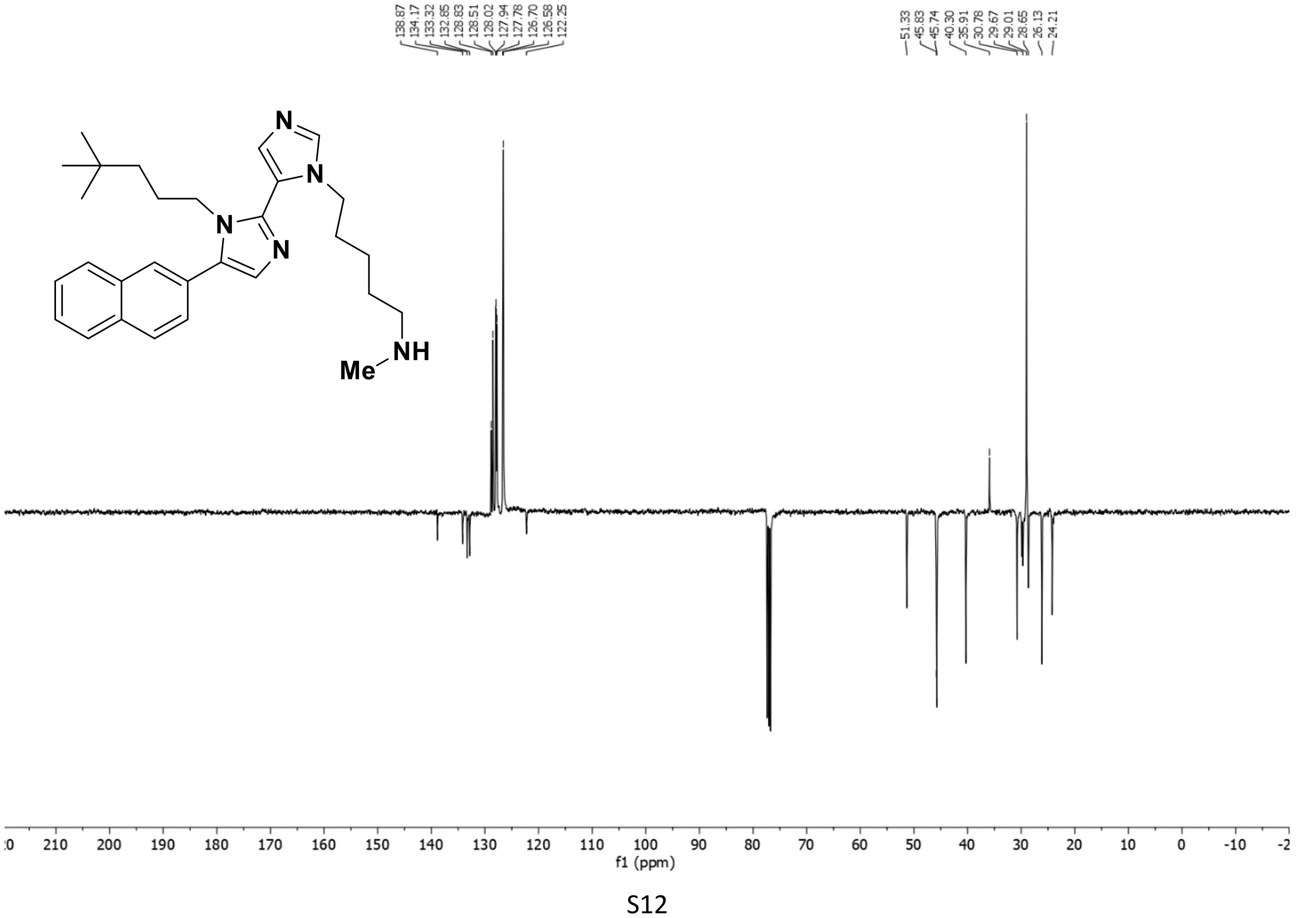

^{1}H NMR (300 MHz, $CDCl_3$) of compound **Dim21**

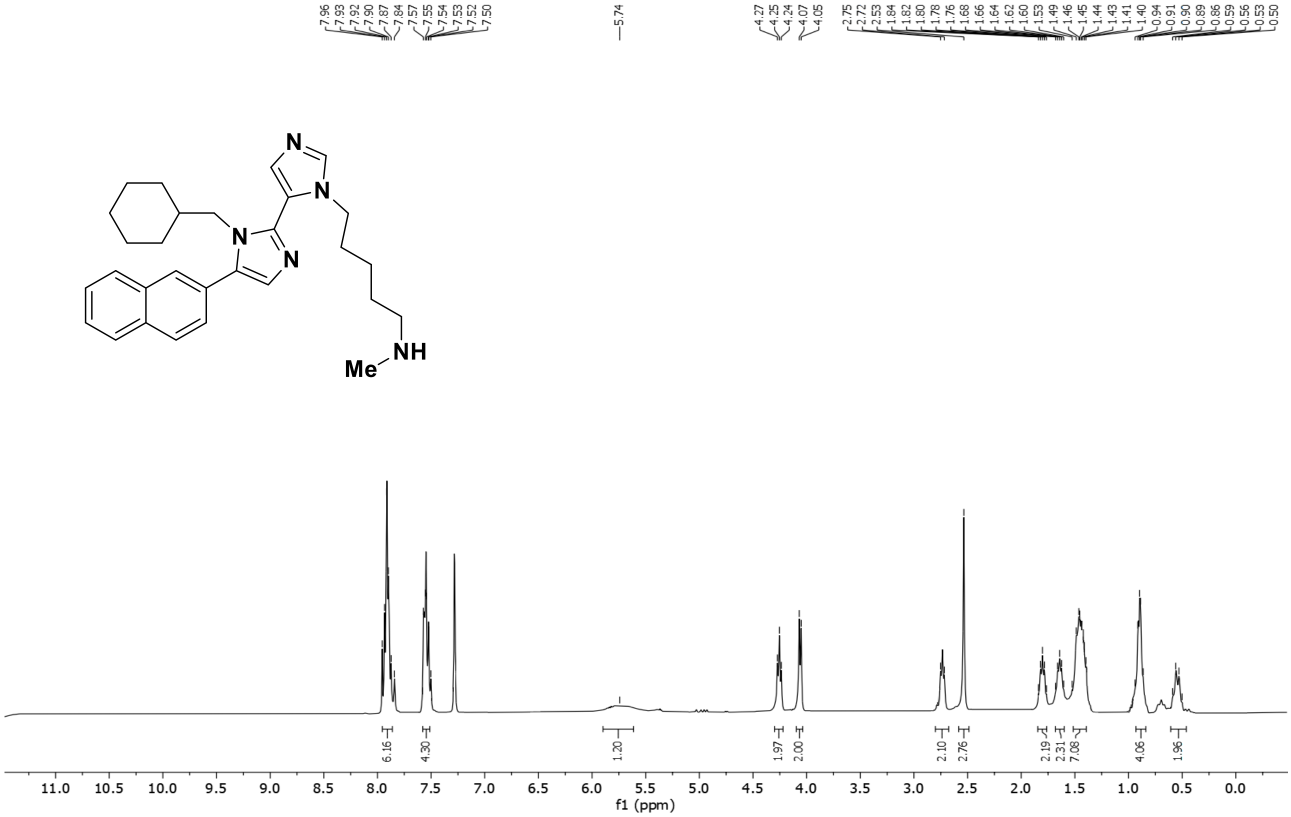

^{13}C NMR (100 MHz, $CDCl_3$) of compound **Dim21**

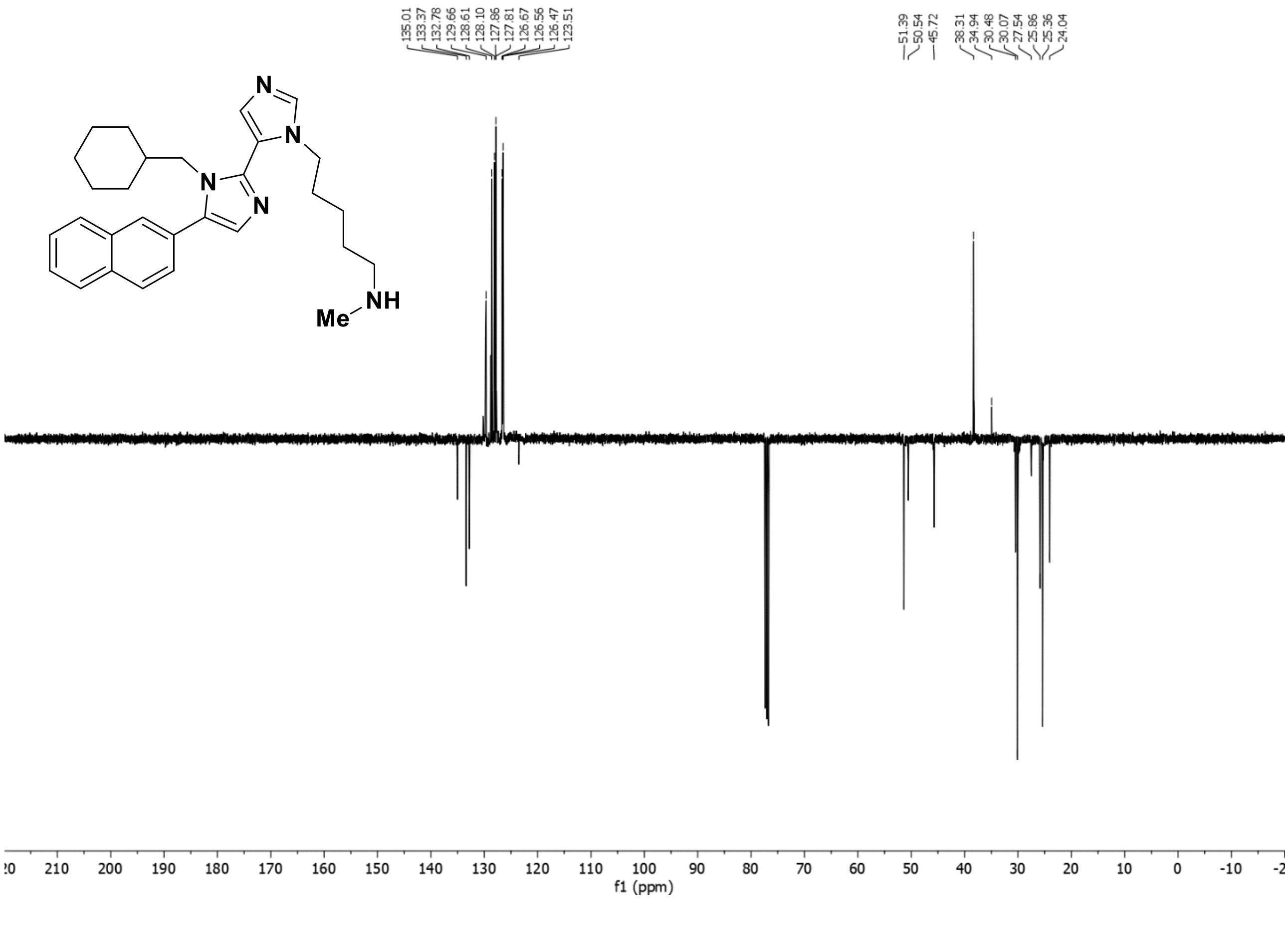

^{1}H NMR (400 MHz, $CDCl_3$) of compound **7**

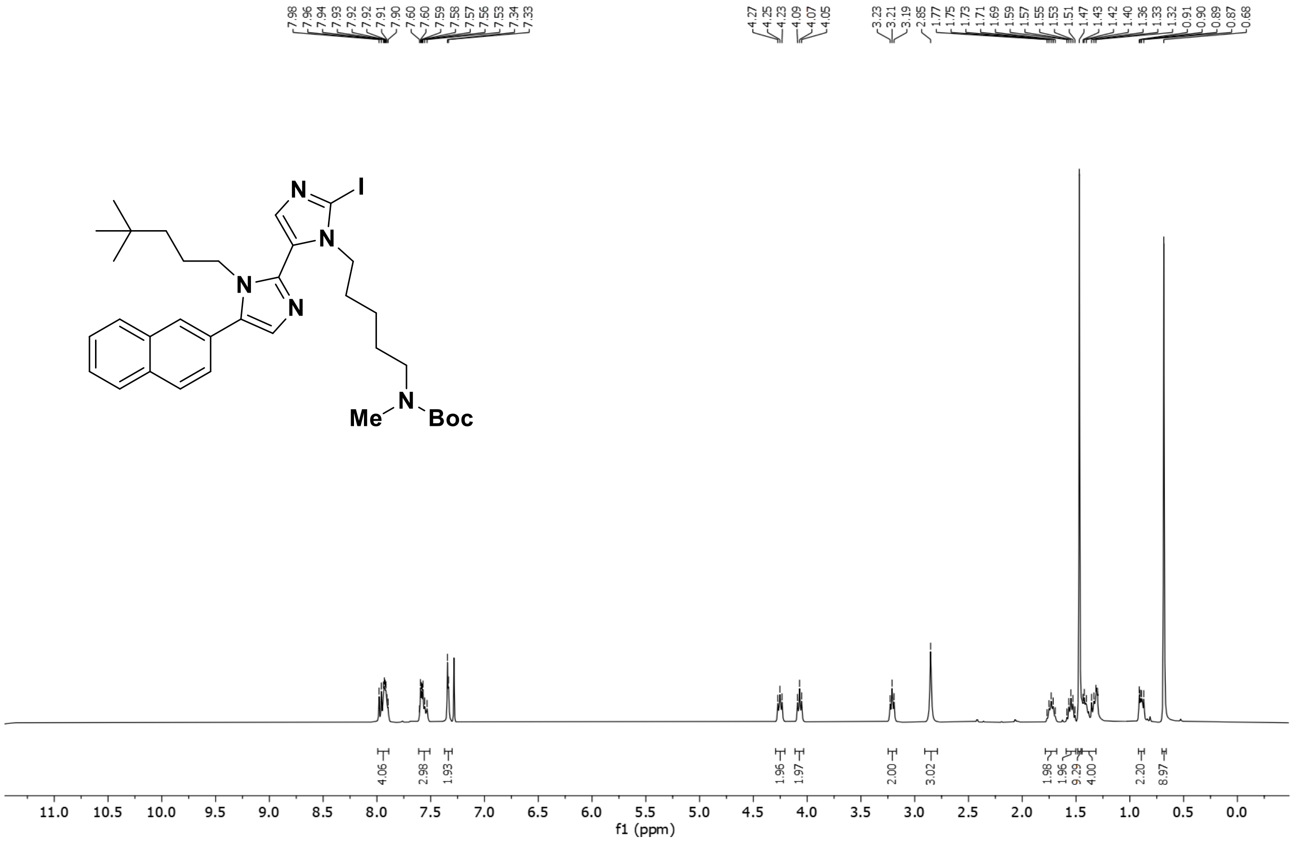

^{13}C NMR (100 MHz, $CDCl_3$) of compound **7**

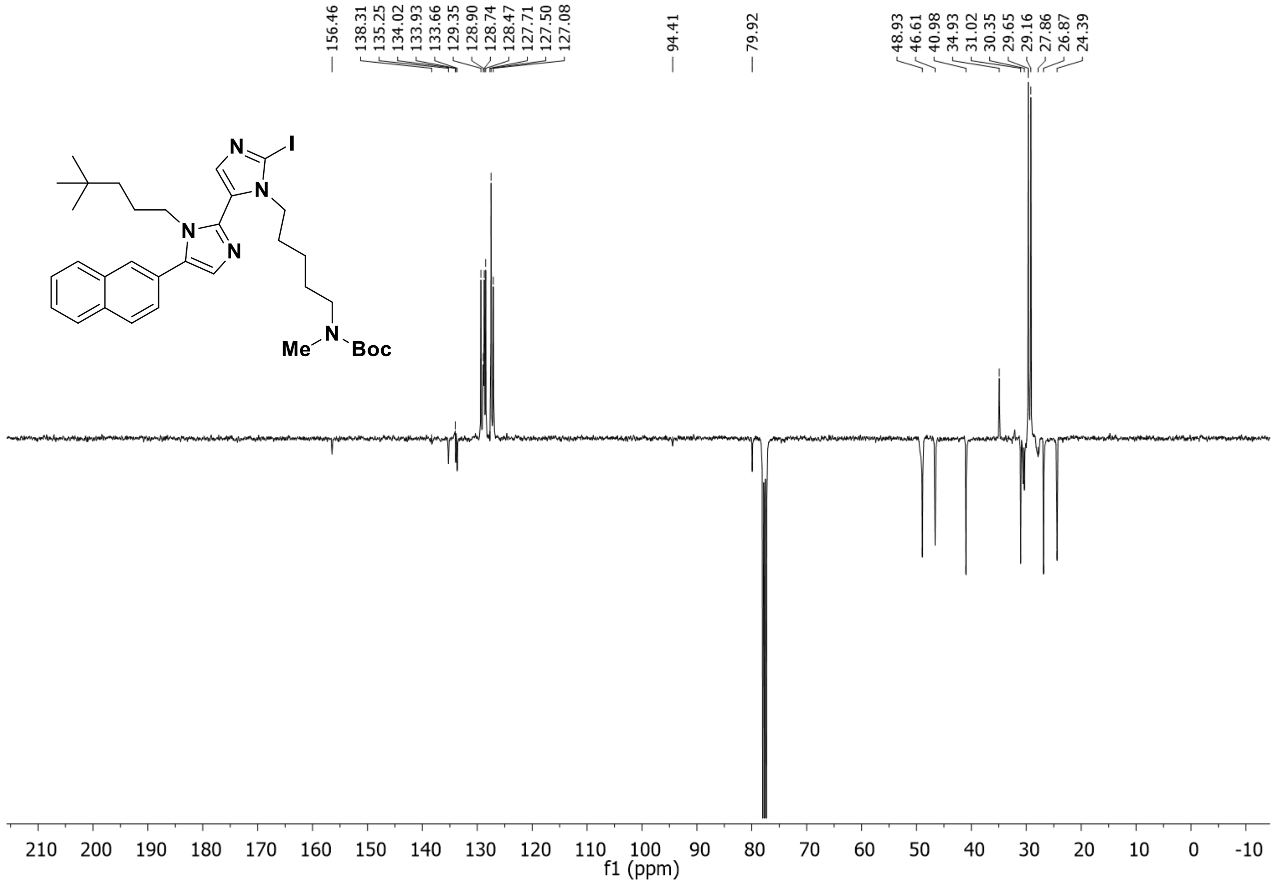

^{1}H NMR (400 MHz, $CDCl_3$) of compound **8**

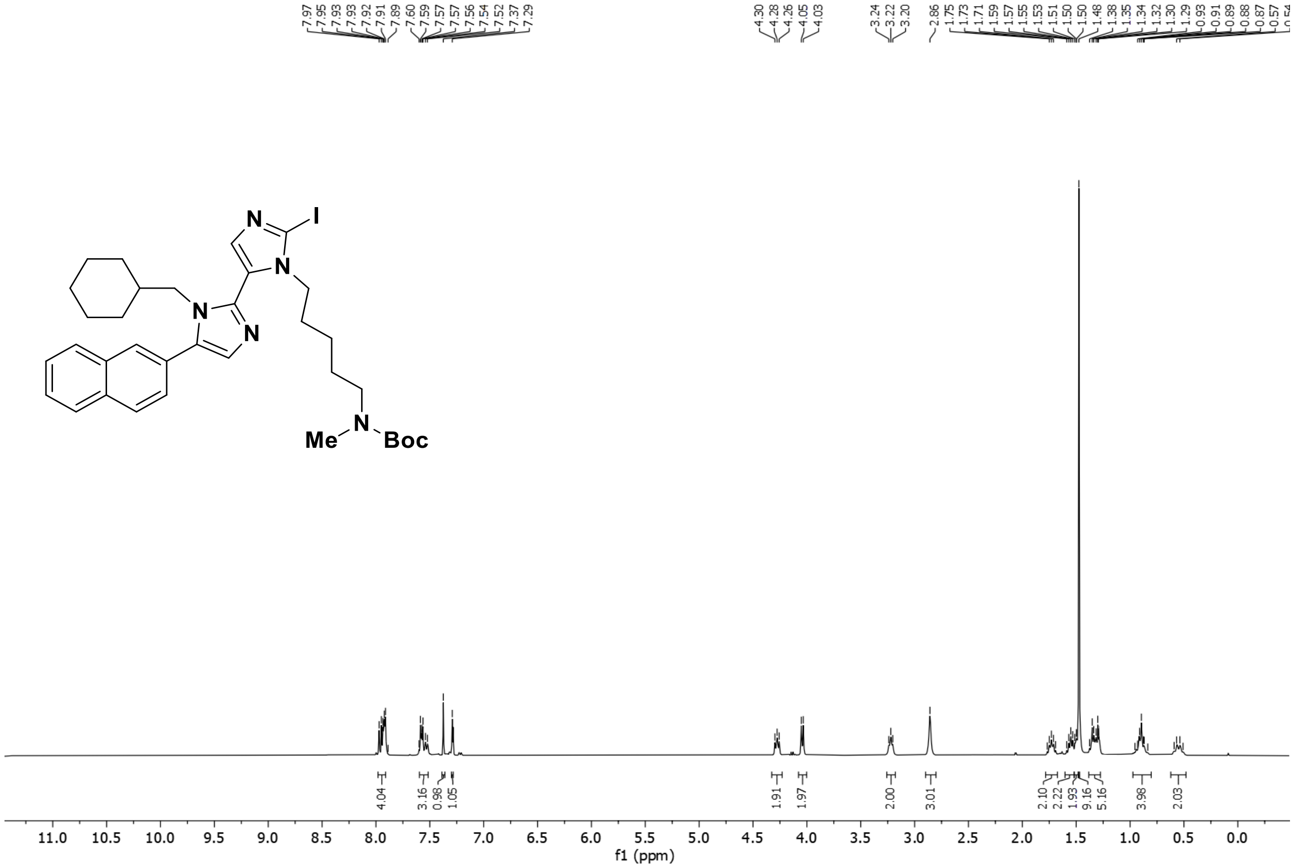

^{13}C NMR (100 MHz, $CDCl_3$) of compound **8**

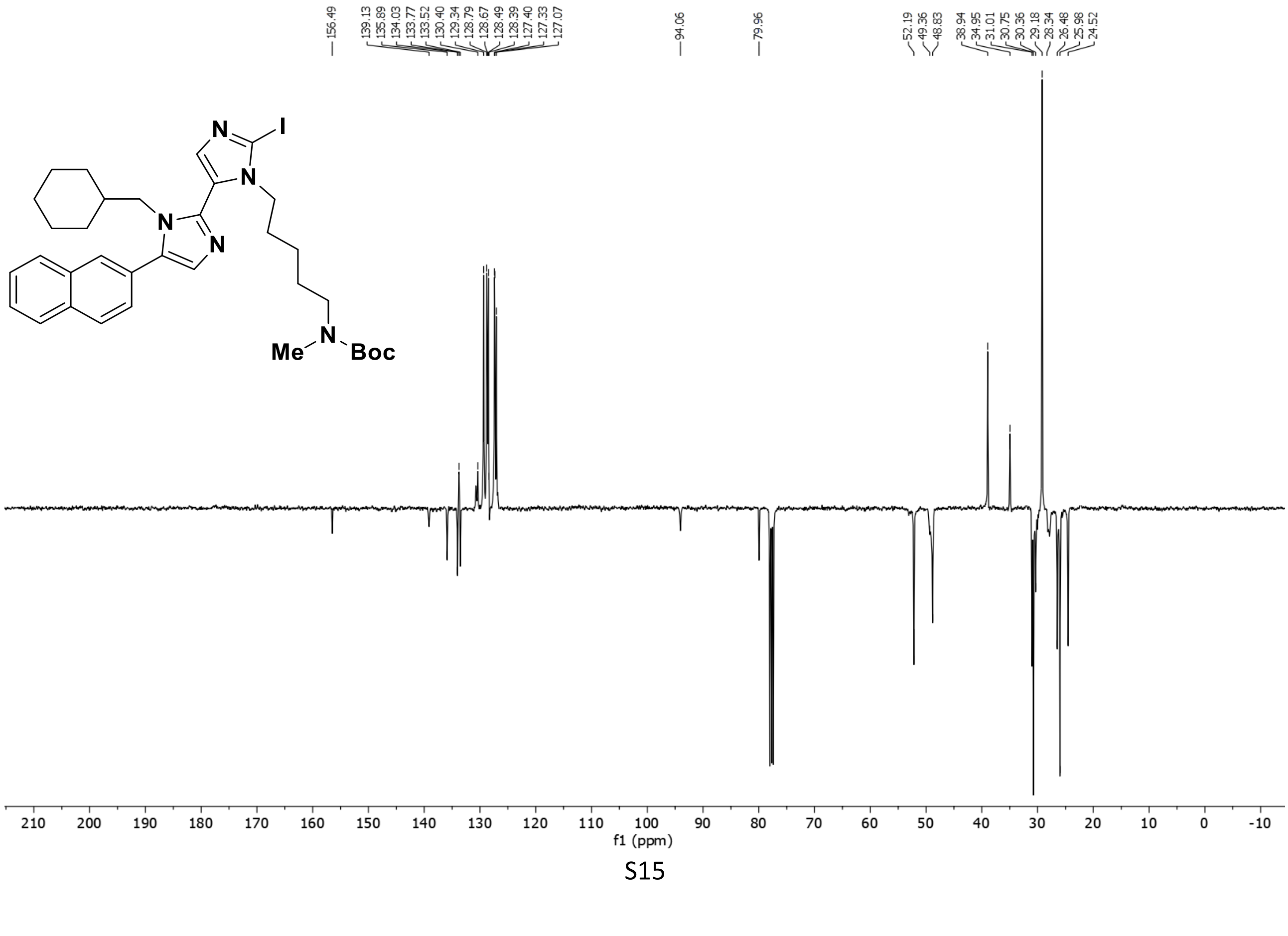

^{1}H NMR (400 MHz, $CDCl_3$) of compound **Dim16**

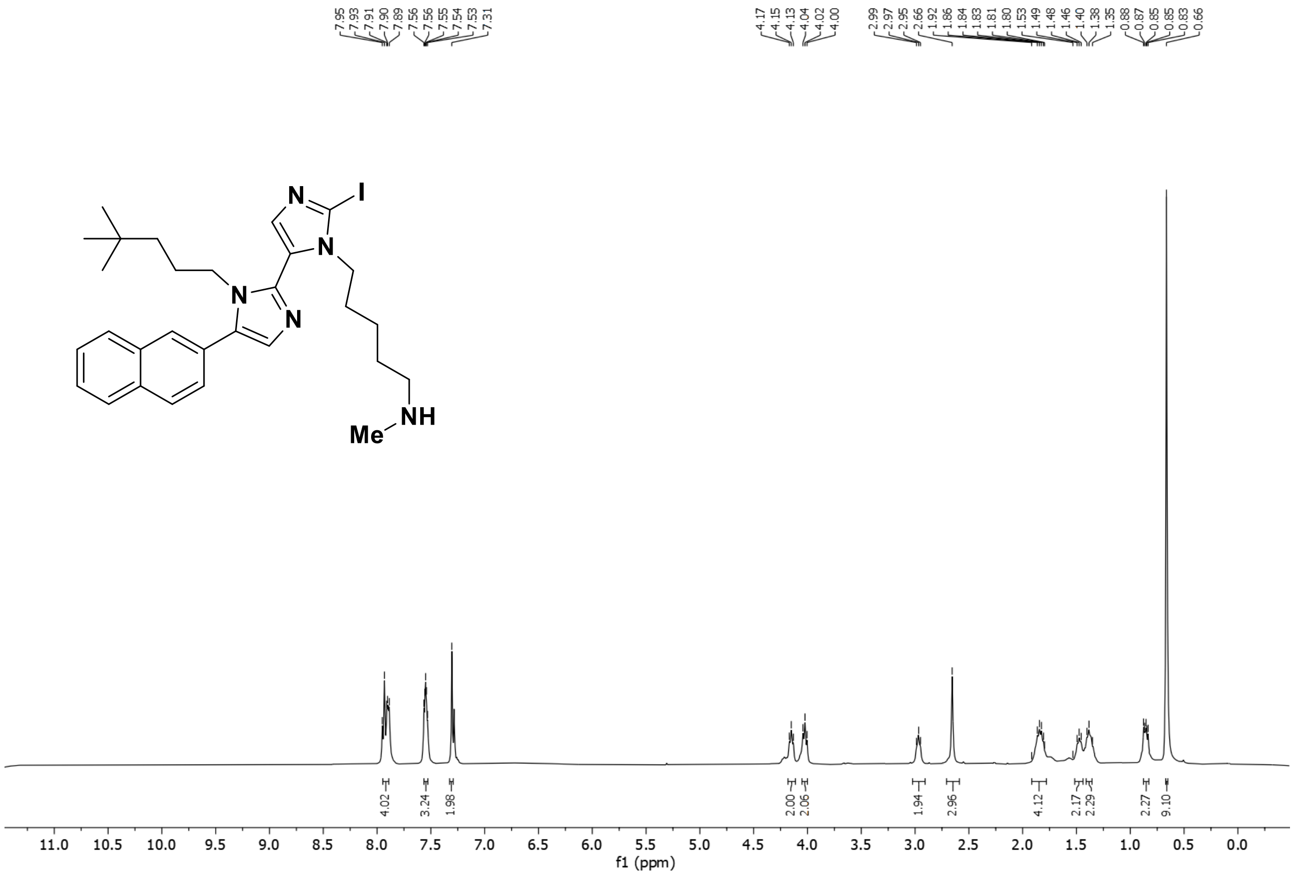

^{13}C NMR (100 MHz, $CDCl_3$) of compound **Dim16**

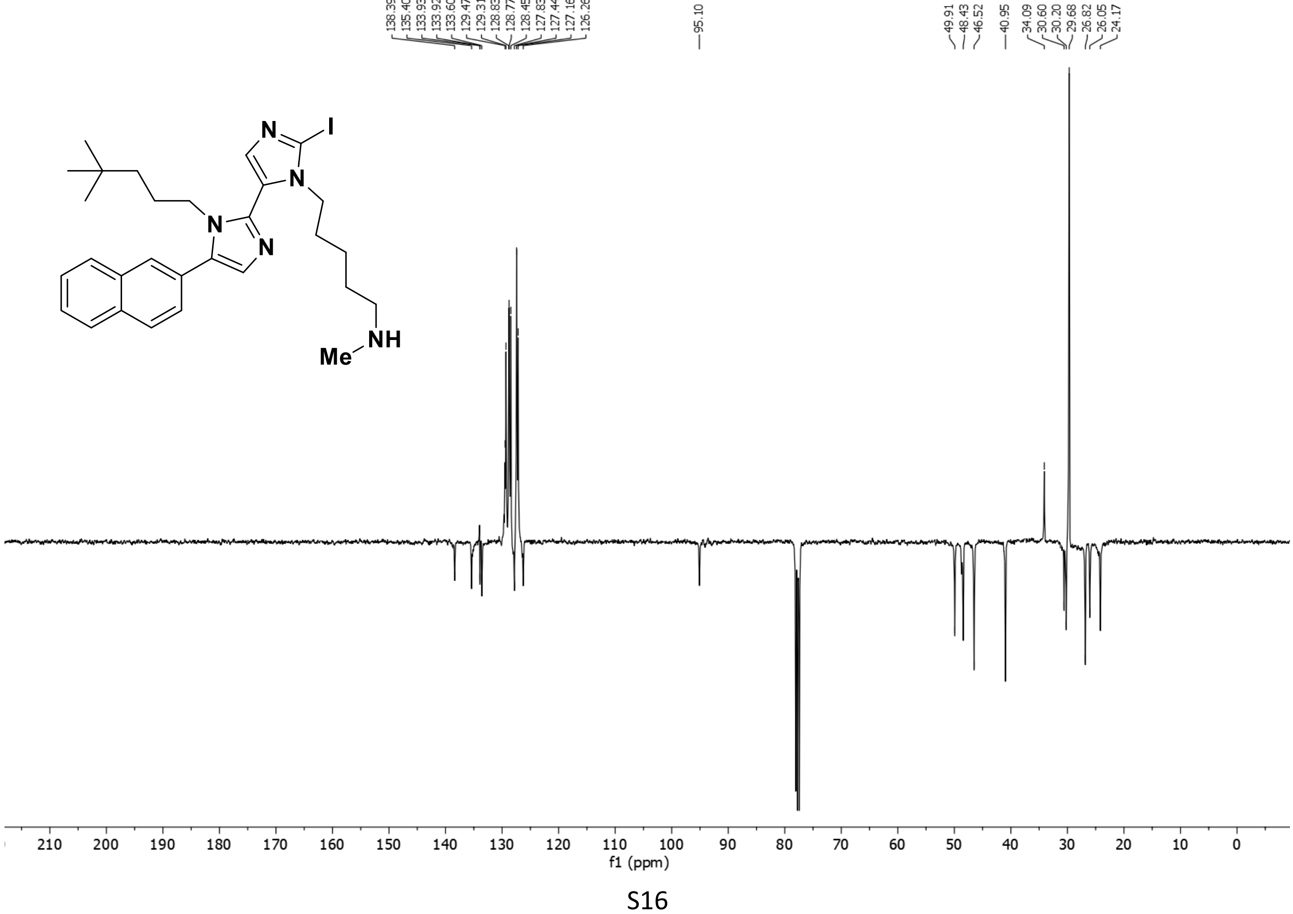

^{1}H NMR (400 MHz, $CDCl_3$) of compound **Dim22**

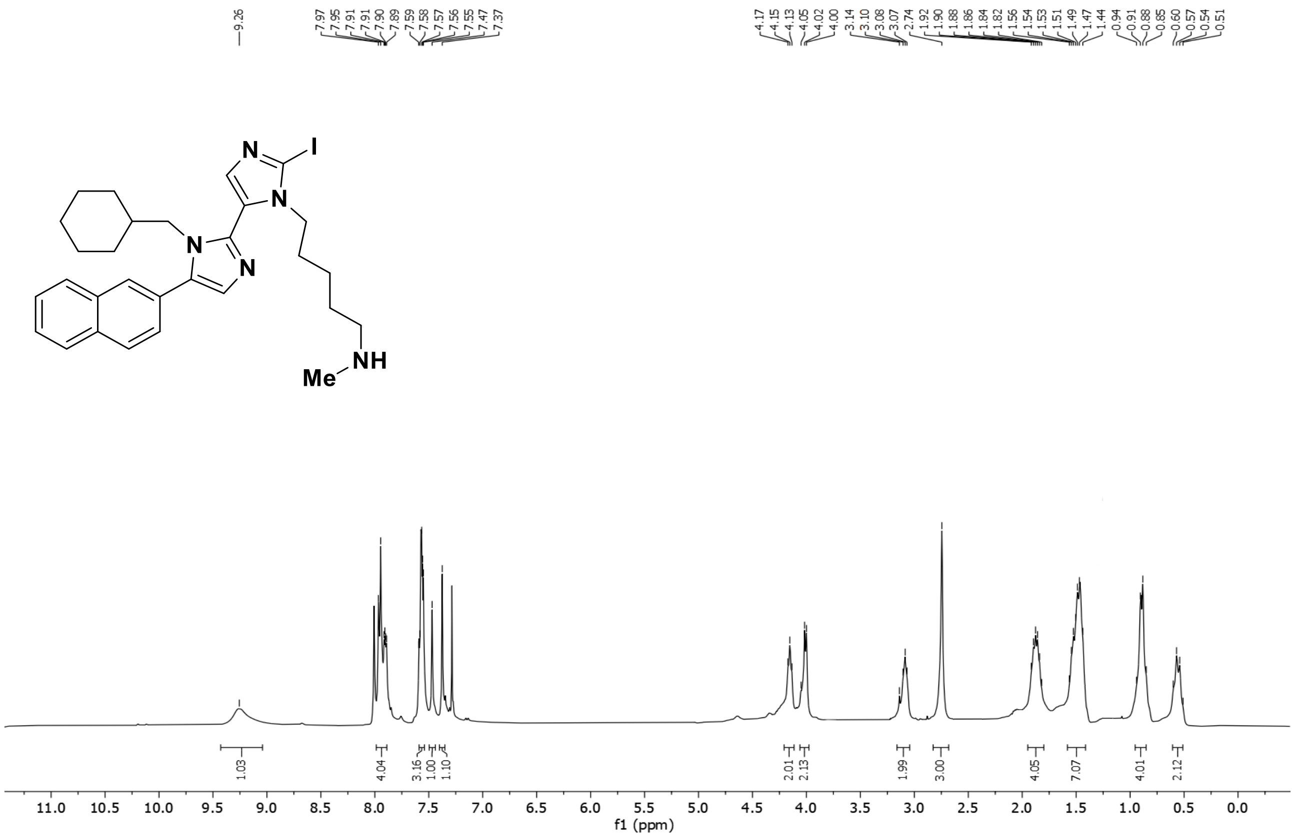

^{13}C NMR (100 MHz, $CDCl_3$) of compound **Dim22**

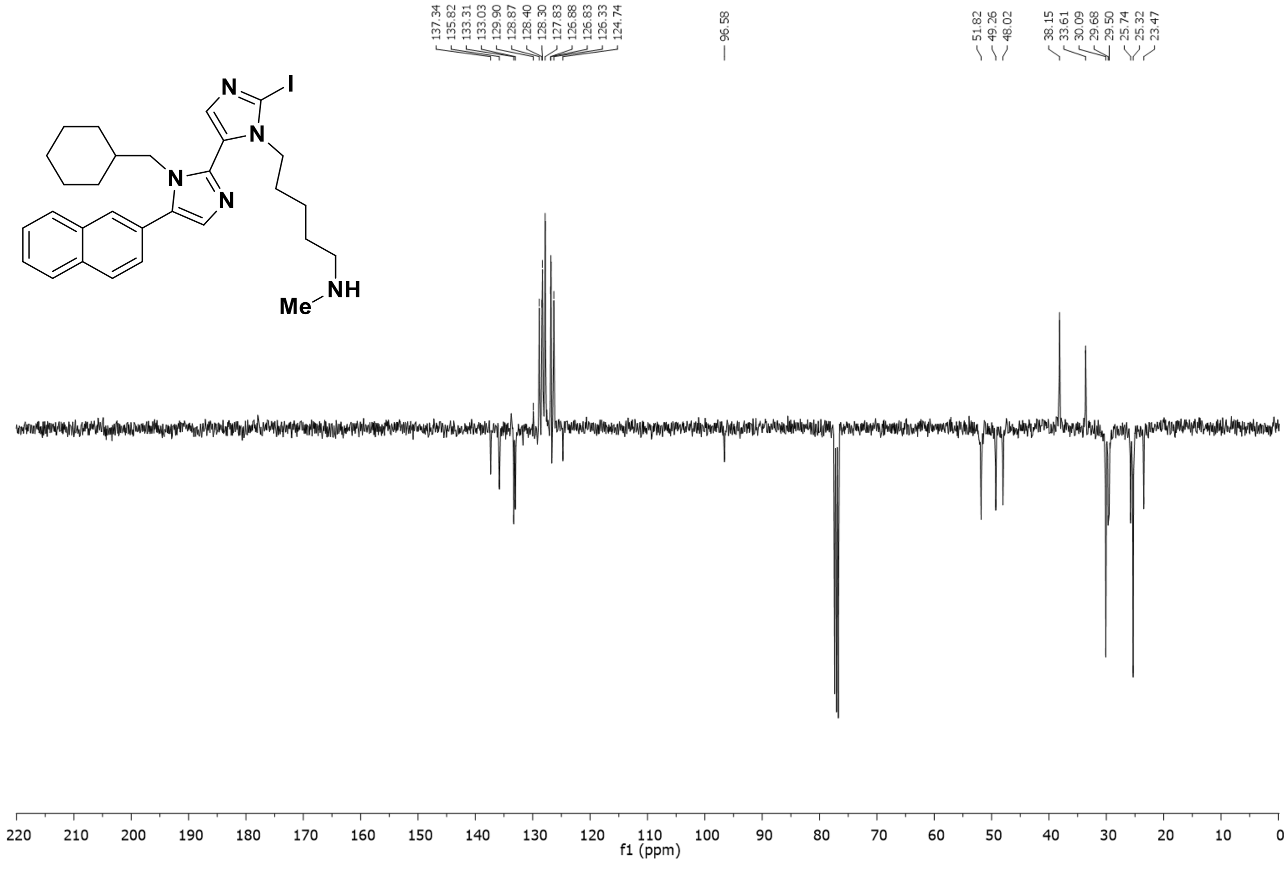

^{1}H NMR (400 MHz, $CDCl_3$) of compound **9**

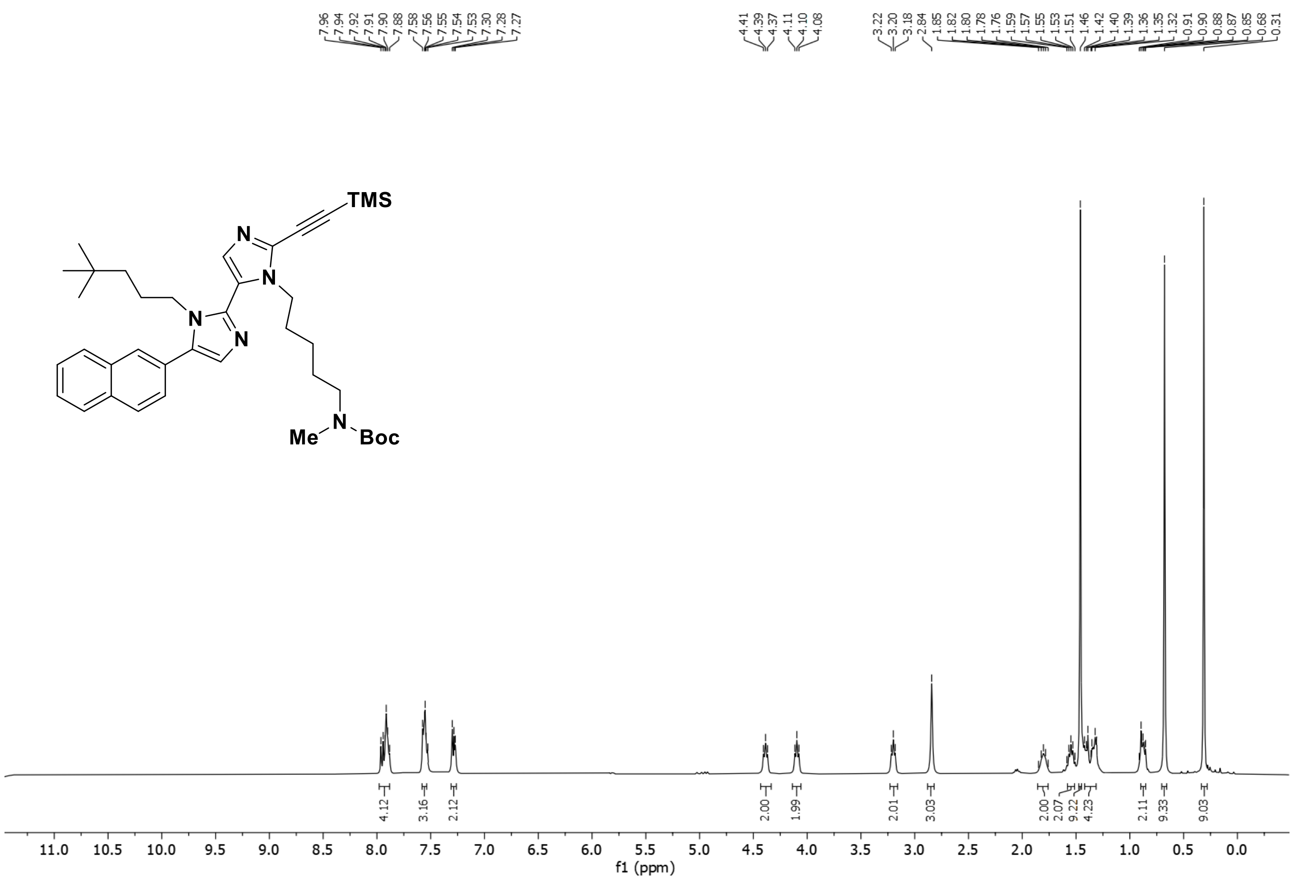

^{13}C NMR (100 MHz, $CDCl_3$) of compound **9**

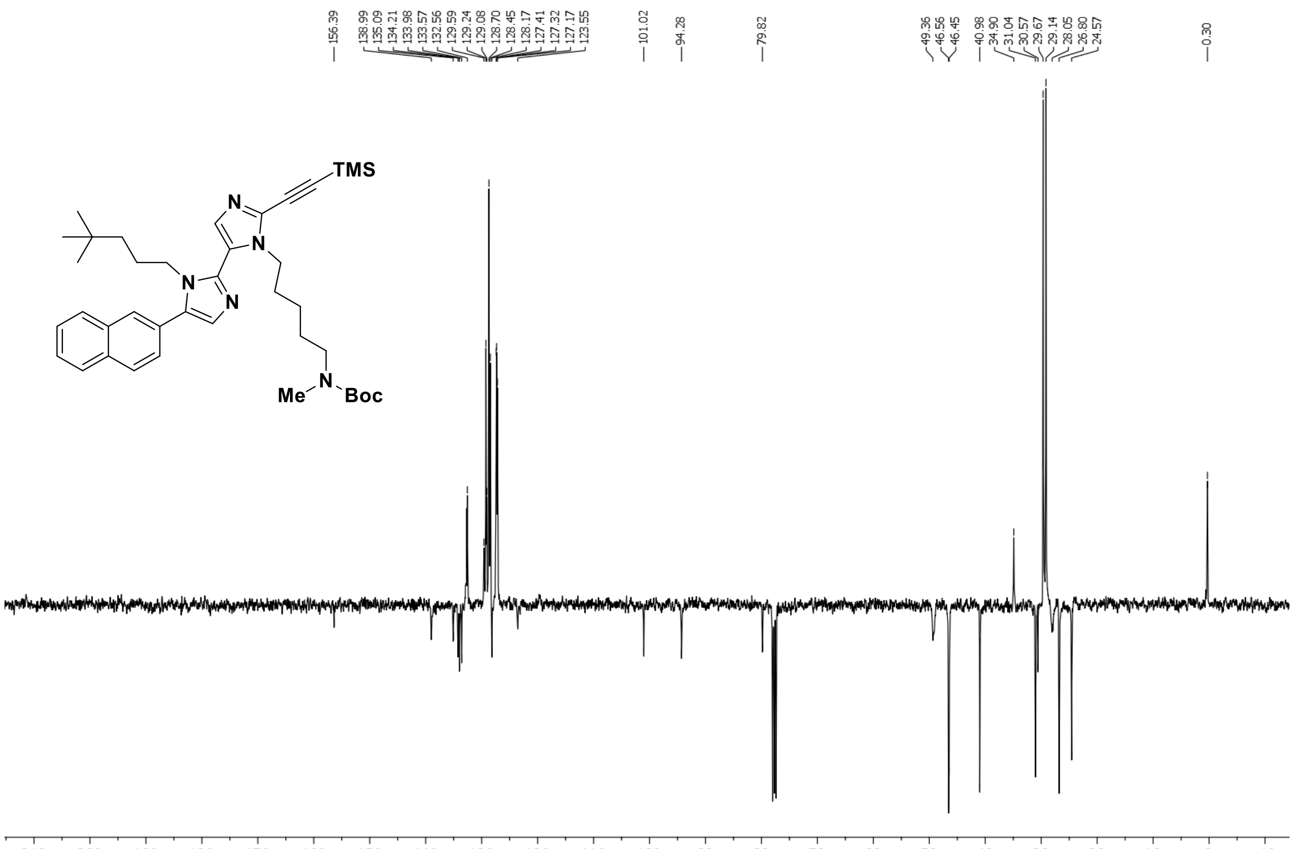

^{1}H NMR (400 MHz, $CDCl_3$) of compound **10**

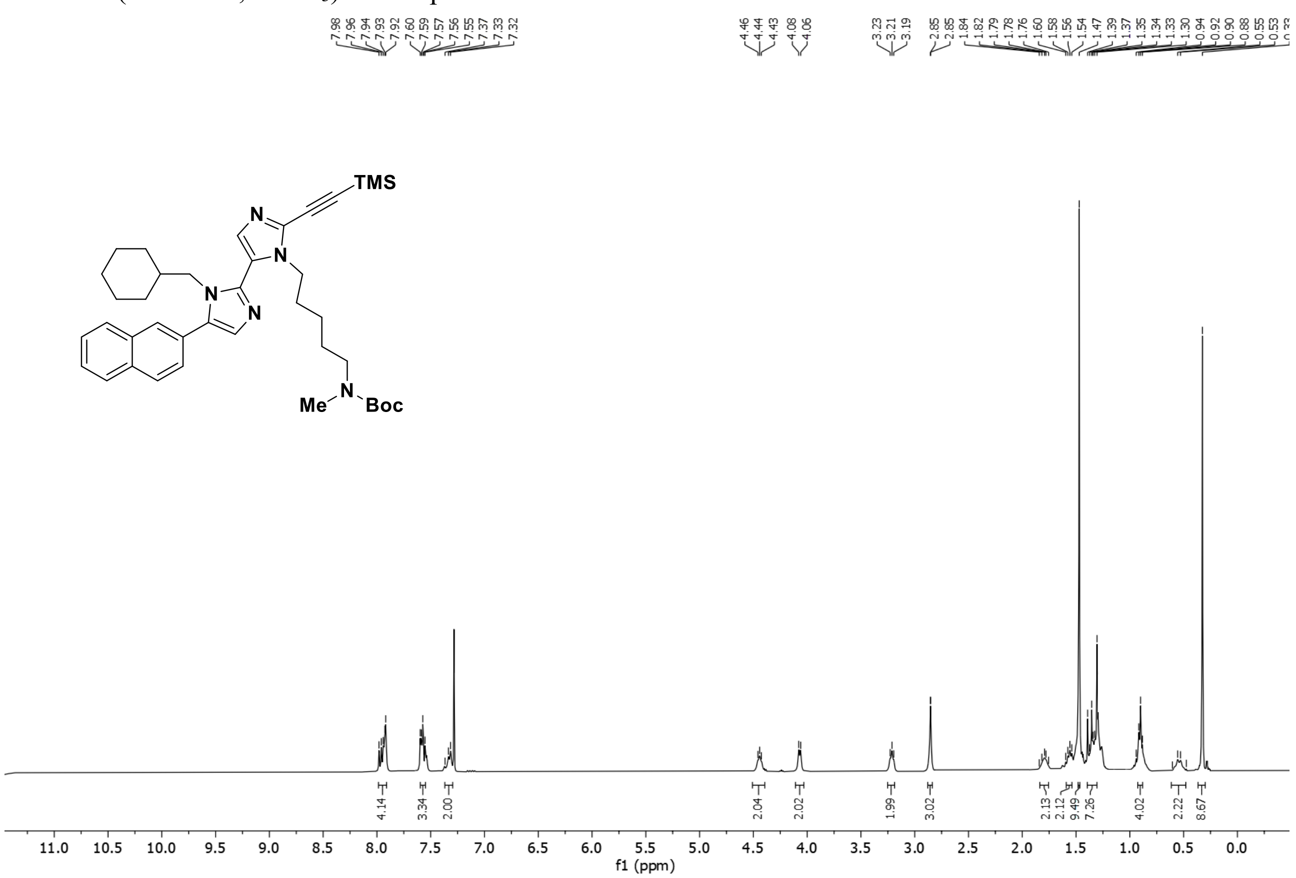

^{13}C NMR (100 MHz, $CDCl_3$) of compound **10**

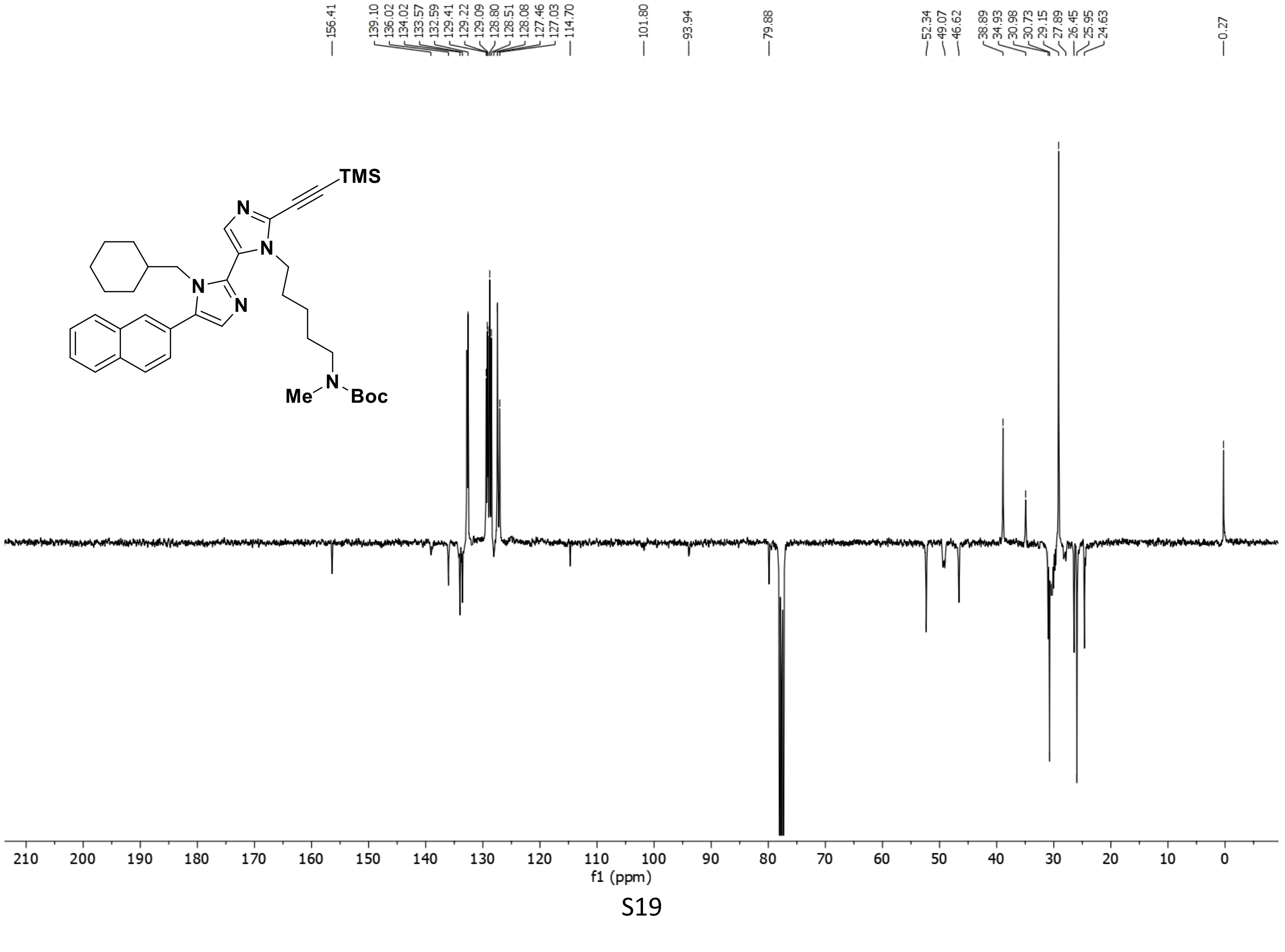

REFERENCE

1. Houston, J.B. Utility of in vitro drug metabolism data in predicting in vivo metabolic clearance. *Biochem Pharmacol* **1994**, *47*, 1469-1479, doi:10.1016/0006-2952(94)90520-7.
2. Riley, R.J.; McGinnity, D.F.; Austin, R.P. A unified model for predicting human hepatic, metabolic clearance from in vitro intrinsic clearance data in hepatocytes and microsomes. *Drug Metab Dispos* **2005**, *33*, 1304-1311, doi:10.1124/dmd.105.004259.
3. Davies, B.; Morris, T. Physiological parameters in laboratory animals and humans. *Pharm Res* **1993**, *10*, 1093-1095, doi:10.1023/a:1018943613122.
